# Supplementary material for: Integrated Molecular Meta-Analysis of 1,000 Pediatric High-Grade and Diffuse Intrinsic Pontine Glioma
Source: Cancer Cell. 2017 Oct 9;32(4):520–537.e5. doi: 10.1016/j.ccell.2017.08.017 (PMC5637314; doi:10.1016/j.ccell.2017.08.017)
Supplement: Document S1. Figures S1–S8 [file mmc1.pdf]

Cancer Cell, Volume 32

Supplemental Information

Integrated Molecular Meta-Analysis

of 1,000 Pediatric High-Grade

and Diffuse Intrinsic Pontine Glioma

Alan Mackay, Anna Burford, Diana Carvalho, Elisa Izquierdo, Janat Fazal-Salom, Kathryn R. Taylor, Lynn Bjerke, Matthew Clarke, Mara Vinci, Meera Nandhabalan, Sara Temelso, Sergey Popov, Valeria Molinari, Pichai Raman, Angela J. Waanders, Harry J. Han, Saumya Gupta, Lynley Marshall, Stergios Zacharoulis, Sucheta Vaidya, Henry C. Mandeville, Leslie R. Bridges, Andrew J. Martin, Safa Al-Sarraj, Christopher Chandler, Ho-Keung Ng, Xingang Li, Kun Mu, Saoussen Trabelsi, Dorra H'mida-Ben Brahim, Alexei N. Kisljakov, Dmitry M. Konovalov, Andrew S. Moore, Angel Montero Carcaboso, Mariona Sunol, Carmen de Torres, Ofelia Cruz, Jaume Mora, Ludmila I. Shats, João N. Stavale, Lucas T. Bidinotto, Rui M. Reis, Natacha Entz-Werle, Michael Farrell, Jane Cryan, Darach Crimmins, John Caird, Jane Pears, Michelle Monje, Marie-Anne Debily, David Castel, Jacques Grill, Cynthia Hawkins, Hamid Nikbakht, Nada Jabado, Suzanne J. Baker, Stefan M. Pfister, David T.W. Jones, Maryam Fouladi, André O. von Bueren, Michael Baudis, Adam Resnick, and Chris Jones

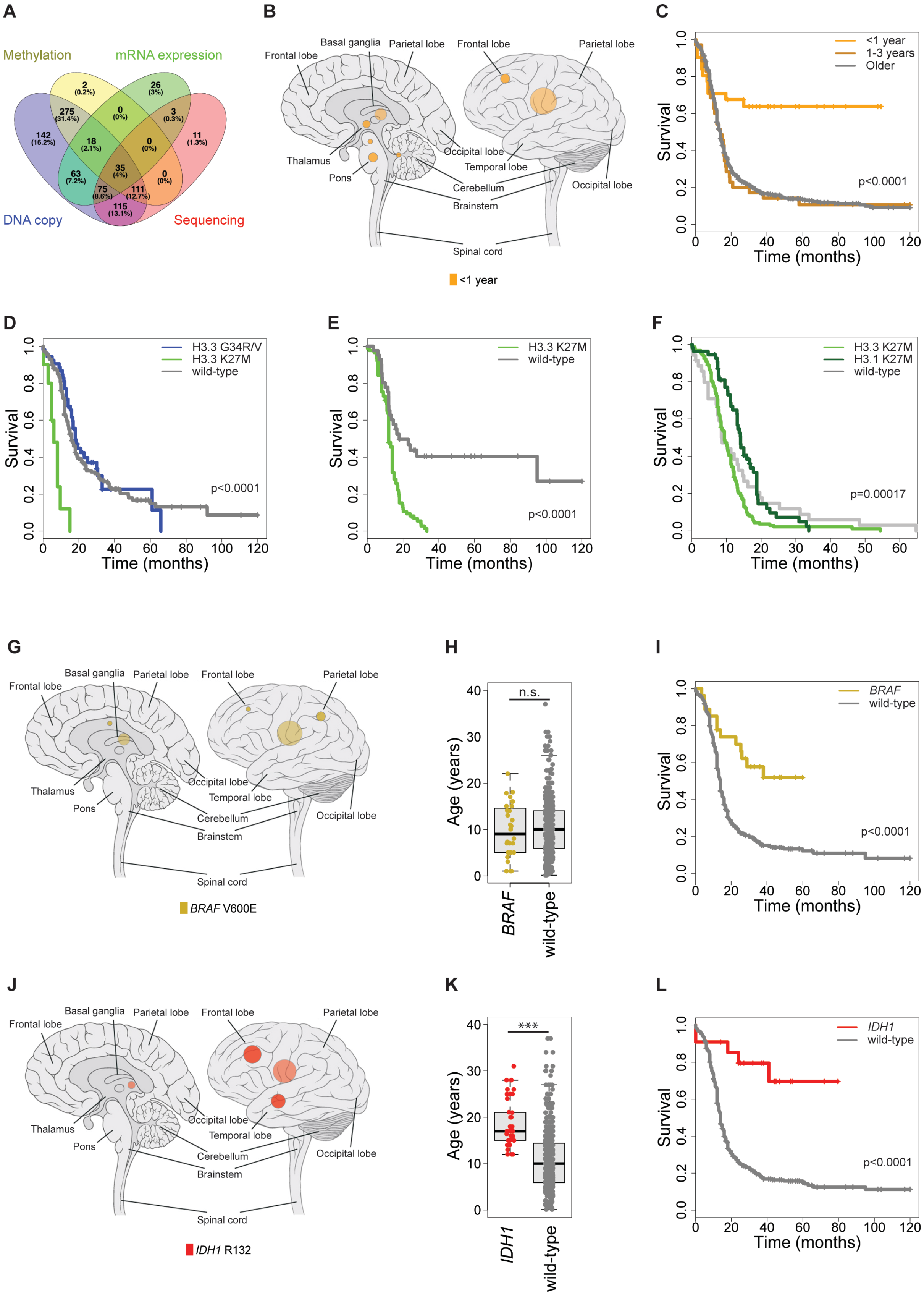
A
Methylation
mRNA expression
DNA copy
Sequencing
2 (0.2%)
26 (3%)
275 (31.4%)
0 (0%)
3 (0.3%)
142 (16.2%)
18 (2.1%)
0 (0%)
11 (1.3%)
35 (4%)
63 (7.2%)
0 (0%)
75 (8.6%)
111 (12.7%)
115 (13.1%)
B
Basal ganglia
Parietal lobe
Frontal lobe
Parietal lobe
Frontal lobe
Thalamus
Pons
Occipital lobe
Temporal lobe
Cerebellum
Occipital lobe
Brainstem
Spinal cord
<1 year
C
<1 year
1-3 years
Older
Survival
Time (months)
p<0.0001
D
H3.3 G34R/V
H3.3 K27M
wild-type
Survival
Time (months)
p<0.0001
E
H3.3 K27M
wild-type
Survival
Time (months)
p<0.0001
F
H3.3 K27M
H3.1 K27M
wild-type
Survival
Time (months)
p=0.00017
G
Basal ganglia
Parietal lobe
Frontal lobe
Parietal lobe
Frontal lobe
Thalamus
Pons
Occipital lobe
Temporal lobe
Cerebellum
Occipital lobe
Brainstem
Spinal cord
BRAF V600E
H
n.s.
Age (years)
BRAF
wild-type
I
BRAF
wild-type
Survival
Time (months)
p<0.0001
J
Basal ganglia
Parietal lobe
Frontal lobe
Parietal lobe
Frontal lobe
Thalamus
Pons
Occipital lobe
Temporal lobe
Cerebellum
Occipital lobe
Brainstem
Spinal cord
IDH1 R132
K
***
Age (years)
IDH1
wild-type
L
IDH1
wild-type
Survival
Time (months)
p<0.0001

Figure S1 (related to Figure 1) – Clinicopathological and molecular subgroups of pHGG/DIPG. (A) Venn diagram showing overlapping molecular data for the cohort. (B) Anatomical location of all cases separated by age at diagnosis <1 year (n=40). Radius of circle is proportional to the number of cases. Left – sagittal section showing internal structures; right – external view highlighting cerebral lobes. Orange = infant. Lighter shaded circles represent a non-specific designation of hemispheric, midline or brainstem. (C) Kaplan-Meier plot of overall survival of cases separated by age at diagnosis <1 year, between 1-3 years, and >3 years of age, p value calculated by the log-rank test (n=818). (D-F) Kaplan-Meier plot of overall survival of cases separated by histone mutation in (D) hemispheric (n=220), (E) midline (n=131), (F) brainstem locations (n=242), p value calculated by the log-rank test. (G) Anatomical location of all cases separated by *BRAF* V600E mutation (n=32, wild-types not shown). Radius of circle is proportional to the number of cases. Left – sagittal section showing internal structures; right – external view highlighting cerebral lobes. Gold = *BRAF* V600E. Lighter shaded circles represent a non-specific designation of hemispheric, midline or brainstem. (H) Boxplot showing age at diagnosis of included cases, separated by *BRAF* V600E mutation (n=553). The lower and upper limits of the boxes represent the first and third quartiles, and the whiskers 1.5x the interquartile range. (I) Kaplan-Meier plot of overall survival of cases separated by *BRAF* V600E, p value calculated by the log-rank test (n=416). (J) Anatomical location of all cases separated by *IDH1* R132 mutations (n=40, wild-types not shown). Radius of circle is proportional to the number of cases. Left – sagittal section showing internal structures; right – external view highlighting cerebral lobes. Red = IDH R132. Lighter shaded circles represent a non-specific designation of hemispheric, midline or brainstem. (K) Boxplot showing age at diagnosis of included cases, separated by *IDH1* R132 mutations (n=672). The thick line within the box is the median, the lower and upper limits of the boxes represent the first and third quartiles, and the whiskers 1.5x the interquartile range. *** p<0.0001, t-test. (L) Kaplan-Meier plot of overall survival of cases separated by *IDH1* R132 mutations, p value calculated by the log-rank test (n=551).

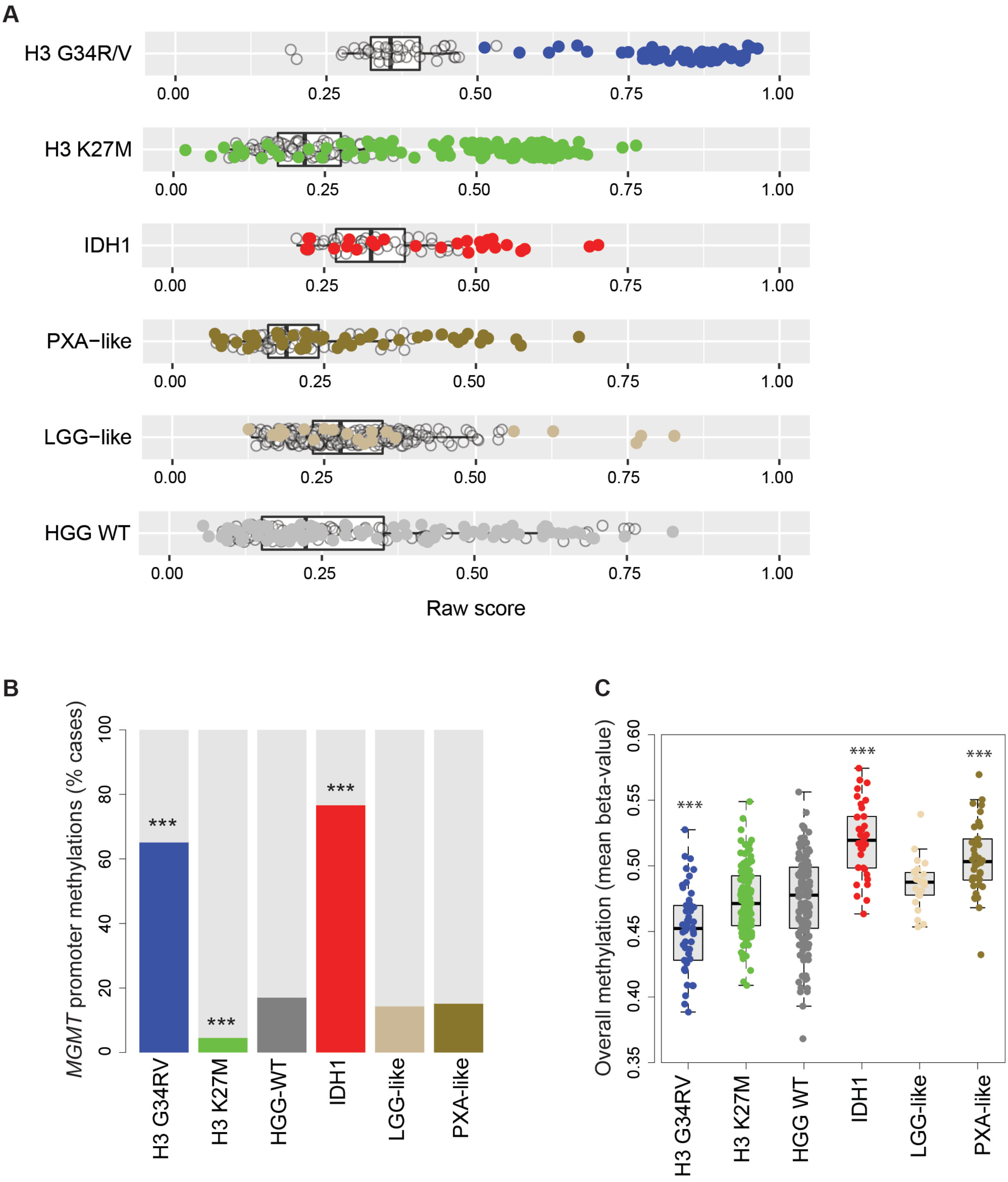

A
H3 G34R/V
H3 K27M
IDH1
PXA−like
LGG−like
HGG WT
0.00
0.25
0.50
0.75
1.00
Raw score
B
MGMT promoter methylations (% cases)
0
20
40
60
80
100
***
***
***
H3 G34RV
H3 K27M
HGG-WT
IDH1
LGG-like
PXA-like
C
Overall methylation (mean beta-value)
0.35
0.40
0.45
0.50
0.55
0.60
***
***
***
H3 G34RV
H3 K27M
HGG WT
IDH1
LGG-like
PXA-like

Figure S2 (related to Figure 2) – Methylation-based subclassification of pHGG/DIPG. (A) Boxplot of Heidelberg brain tumor subclassifier scores for pediatric HGG in the present study (solid circles, n=441) assigned to one of six HGG subgroups defined by a reference set (open circles). The thick line within the box is the median, the lower and upper limits of the boxes represent the first and third quartiles, and the whiskers 1.5x the interquartile range. H3 G34R/V – blue; H3 K27M – green; IDH1 – red; PXA-like – gold; LGG-like – tan; HGG WT – grey. (B) Barplots of number of cases with methylated *MGMT* promoter, subdivided by methylation subgroup (n=366). (C) Boxplots of mean methylation beta-values for cases subdivided by methylation subgroup (n=441). The thick line within the box is the median, the lower and upper limits of the boxes represent the first and third quartiles, and the whiskers 1.5x the interquartile range. *** $p<0.001$, ** $p<0.01$, *$p<0.05$ *versus* rest, t-test.

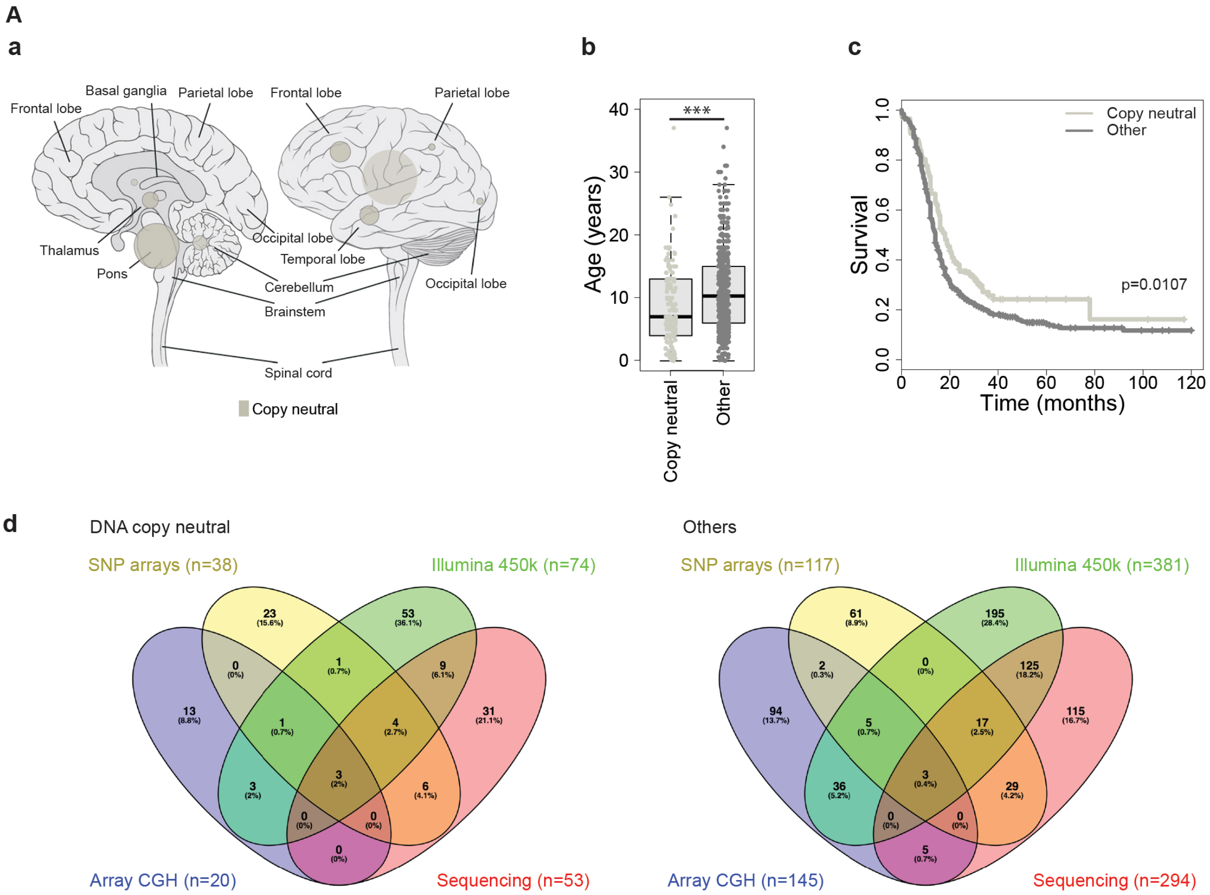

A
a
Frontal lobe
Basal ganglia
Parietal lobe
Frontal lobe
Parietal lobe
Thalamus
Pons
Occipital lobe
Temporal lobe
Cerebellum
Brainstem
Occipital lobe
Spinal cord
Copy neutral
b
***
Age (years)
40
30
20
10
0
Copy neutral
Other
c
Copy neutral
Other
Survival
1.0
0.8
0.6
0.4
0.2
0.0
p=0.0107
0
20
40
60
80
100
120
Time (months)
d
DNA copy neutral
SNP arrays (n=38)
Illumina 450k (n=74)
Array CGH (n=20)
Sequencing (n=53)
Others
SNP arrays (n=117)
Illumina 450k (n=381)
Array CGH (n=145)
Sequencing (n=294)

B

a

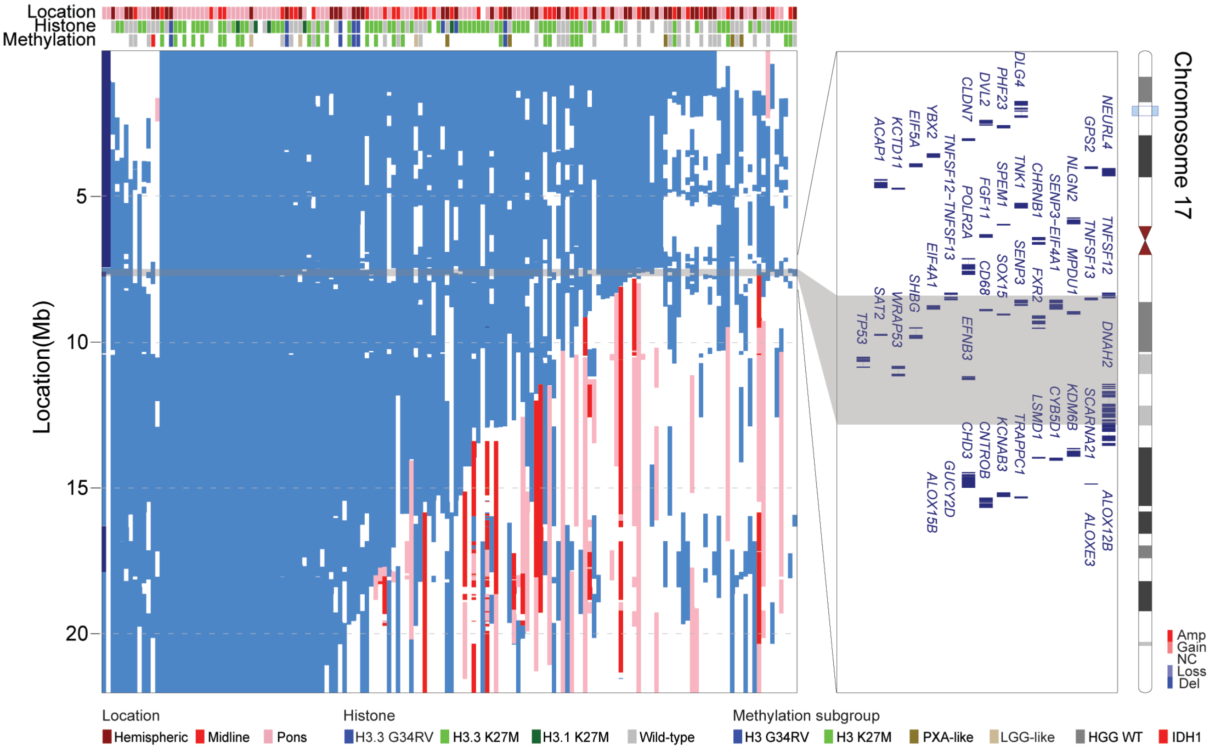

Location
Histone
Methylation
Location(Mb)
5
10
15
20
Chromosome 17
Amp
Gain
NC
Loss
Del
Location
Hemispheric
Midline
Pons
Histone
H3.3 G34RV
H3.3 K27M
H3.1 K27M
Wild-type
Methylation subgroup
H3 G34RV
H3 K27M
PXA-like
LGG-like
HGG WT
IDH1

b

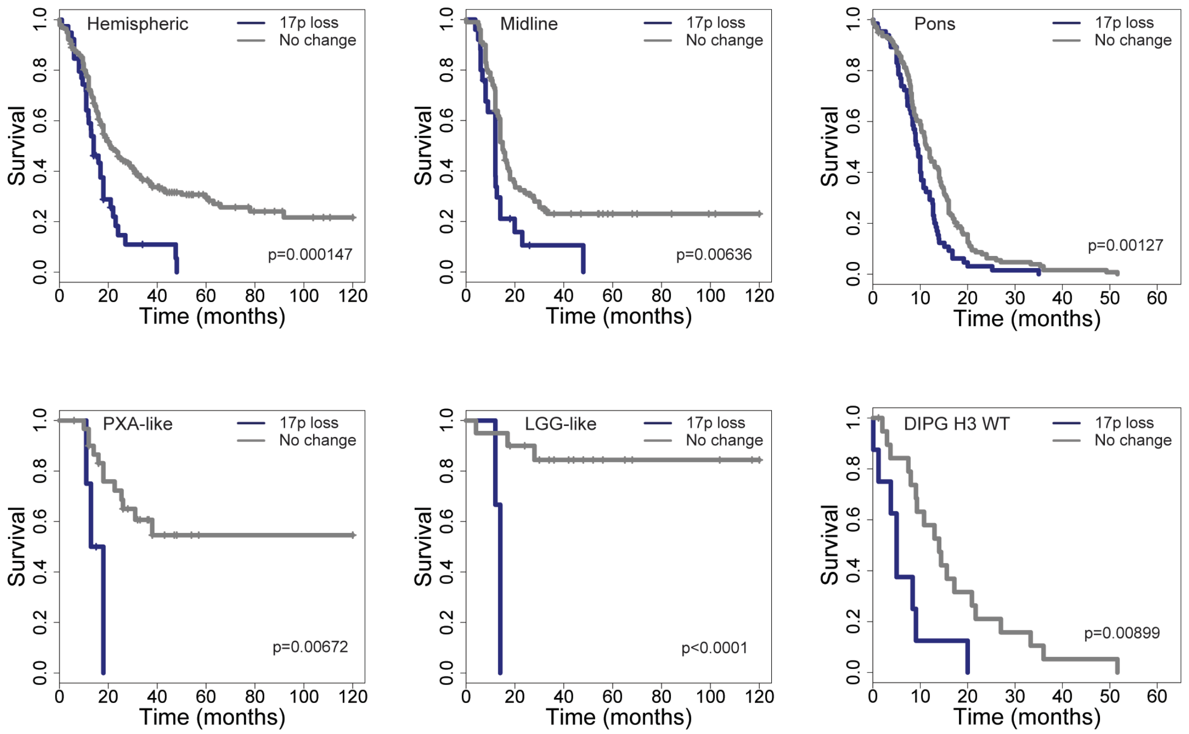

Hemispheric
Midline
Pons
PXA-like
LGG-like
DIPG H3 WT
17p loss
No change
p=0.000147
p=0.00636
p=0.00127
p=0.00672
p<0.0001
p=0.00899
Survival
Time (months)

C

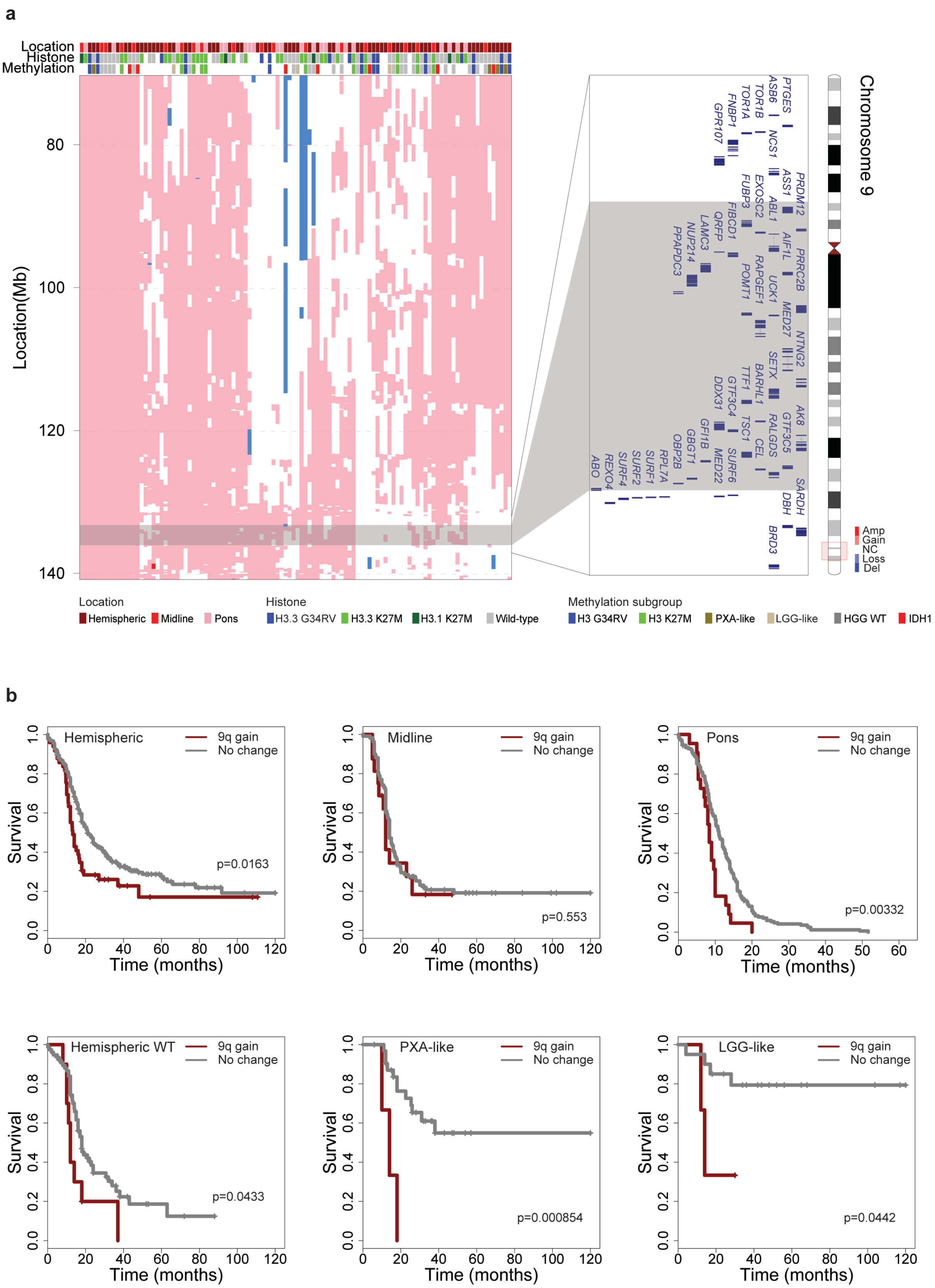
a
Location
Histone
Methylation
Location(Mb)
80
100
120
140
Chromosome 9
Amp
Gain
NC
Loss
Del
Location
Hemispheric
Midline
Pons
Histone
H3.3 G34RV
H3.3 K27M
H3.1 K27M
Wild-type
Methylation subgroup
H3 G34RV
H3 K27M
PXA-like
LGG-like
HGG WT
IDH1
b
Hemispheric
Midline
Pons
Hemispheric WT
PXA-like
LGG-like
9q gain
No change
Survival
Time (months)
p=0.0163
p=0.553
p=0.00332
p=0.0433
p=0.000854
p=0.0442

D

a

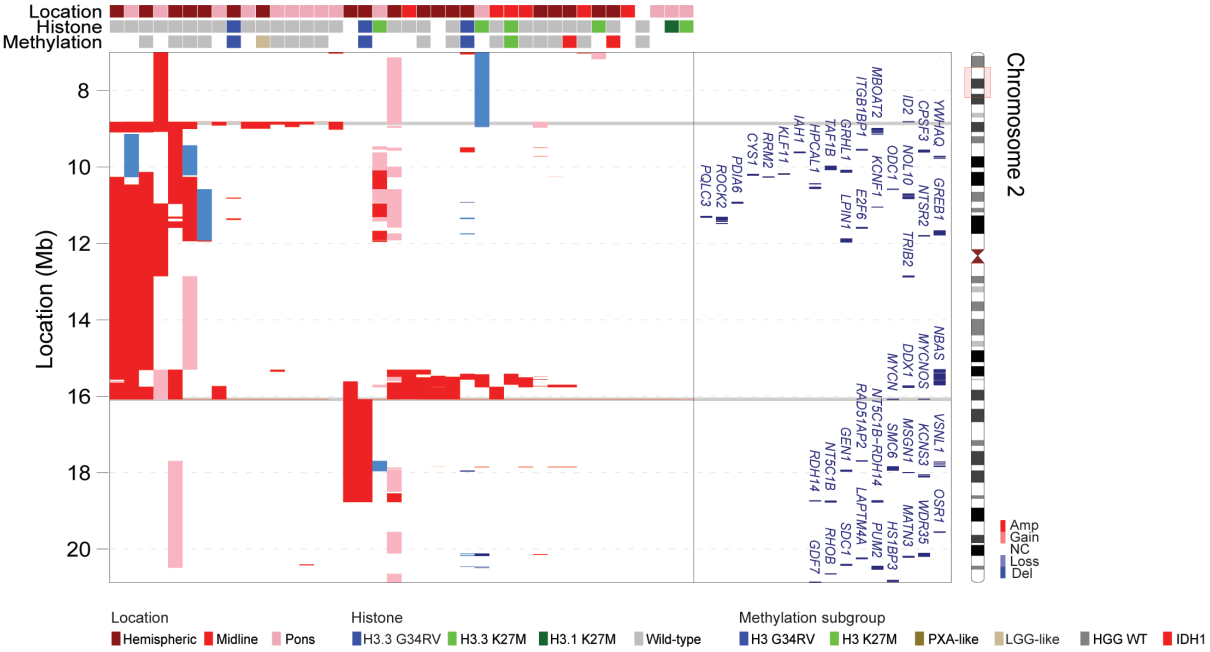
Location
Histone
Methylation
Location (Mb)
8
10
12
14
16
18
20
Chromosome 2
YWHAQ
CPSF3
ID2
MBOAT2
ITGB1BP1
GREB1
NTSR2
NOL10
ODC1
KCNF1
E2F6
LPIN1
TRIB2
GRHL1
TAF1B
HPCAL1
IAH1
KLF11
RRM2
CYS1
PDIA6
ROCK2
PQLC3
NBAS
MYCNOS
DDX1
MYCN
VSNL1
KCNS3
MSGN1
SMC6
NT5C1B-RDH14
RAD51AP2
GEN1
NT5C1B
RDH14
OSR1
WDR35
MATN3
HS1BP3
PUM2
LAPTM4A
SDC1
RHOB
GDF7
Amp
Gain
NC
Loss
Del
Location
Hemispheric
Midline
Pons
Histone
H3.3 G34RV
H3.3 K27M
H3.1 K27M
Wild-type
Methylation subgroup
H3 G34RV
H3 K27M
PXA-like
LGG-like
HGG WT
IDH1

b

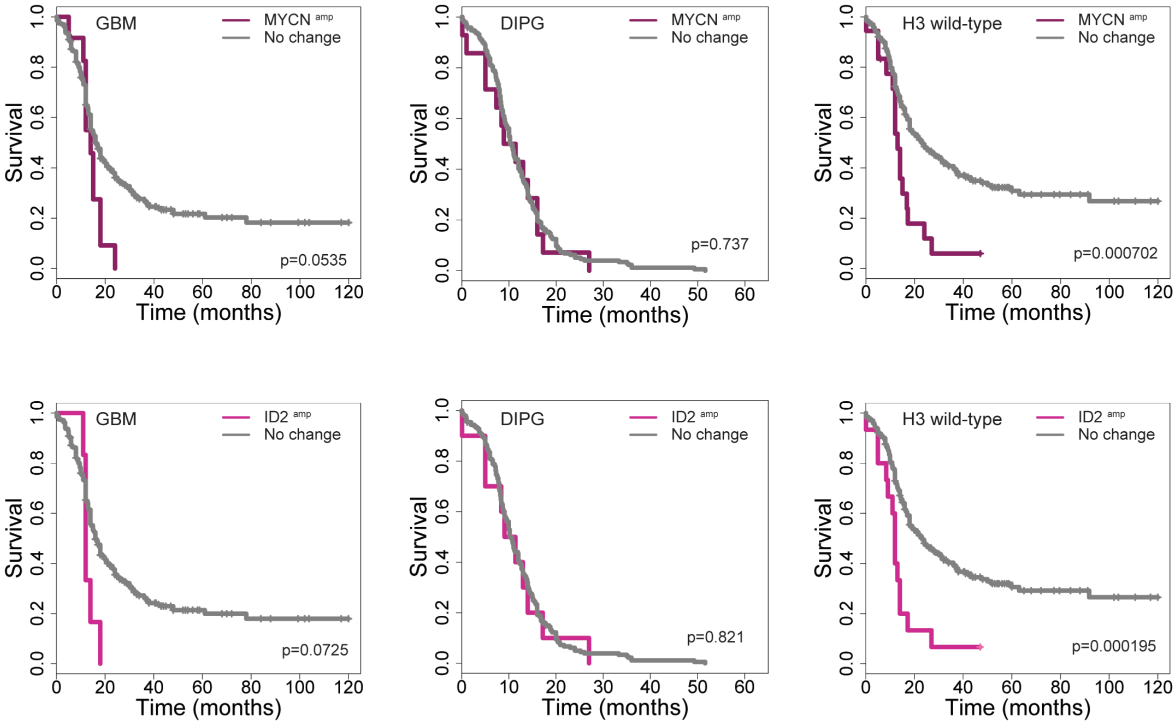
GBM
DIPG
H3 wild-type
MYCN amp
ID2 amp
No change
Survival
Time (months)
p=0.0535
p=0.737
p=0.000702
p=0.0725
p=0.821
p=0.000195

E

a

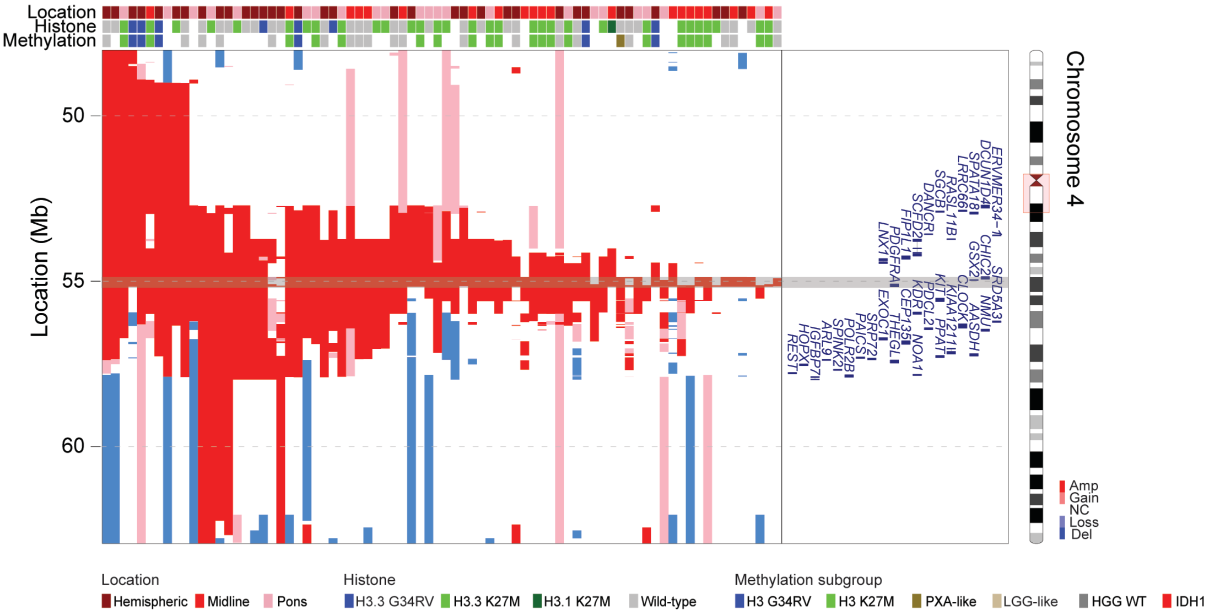

Location
Histone
Methylation
Location (Mb)
50
55
60
Chromosome 4
Amp
Gain
NC
Loss
Del
Location
Hemispheric
Midline
Pons
Histone
H3.3 G34RV
H3.3 K27M
H3.1 K27M
Wild-type
Methylation subgroup
H3 G34RV
H3 K27M
PXA-like
LGG-like
HGG WT
IDH1

b

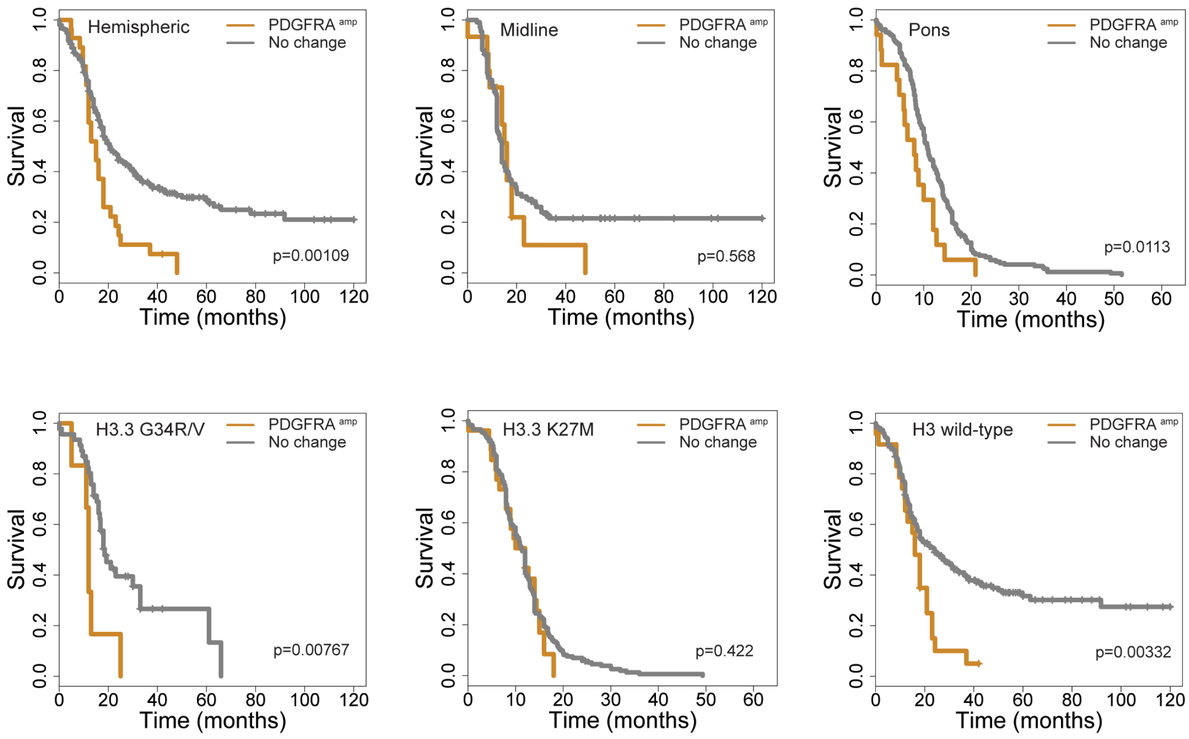

Hemispheric
PDGFRA amp
No change
p=0.00109
Midline
p=0.568
Pons
p=0.0113
H3.3 G34R/V
p=0.00767
H3.3 K27M
p=0.422
H3 wild-type
p=0.00332
Survival
Time (months)

F

a

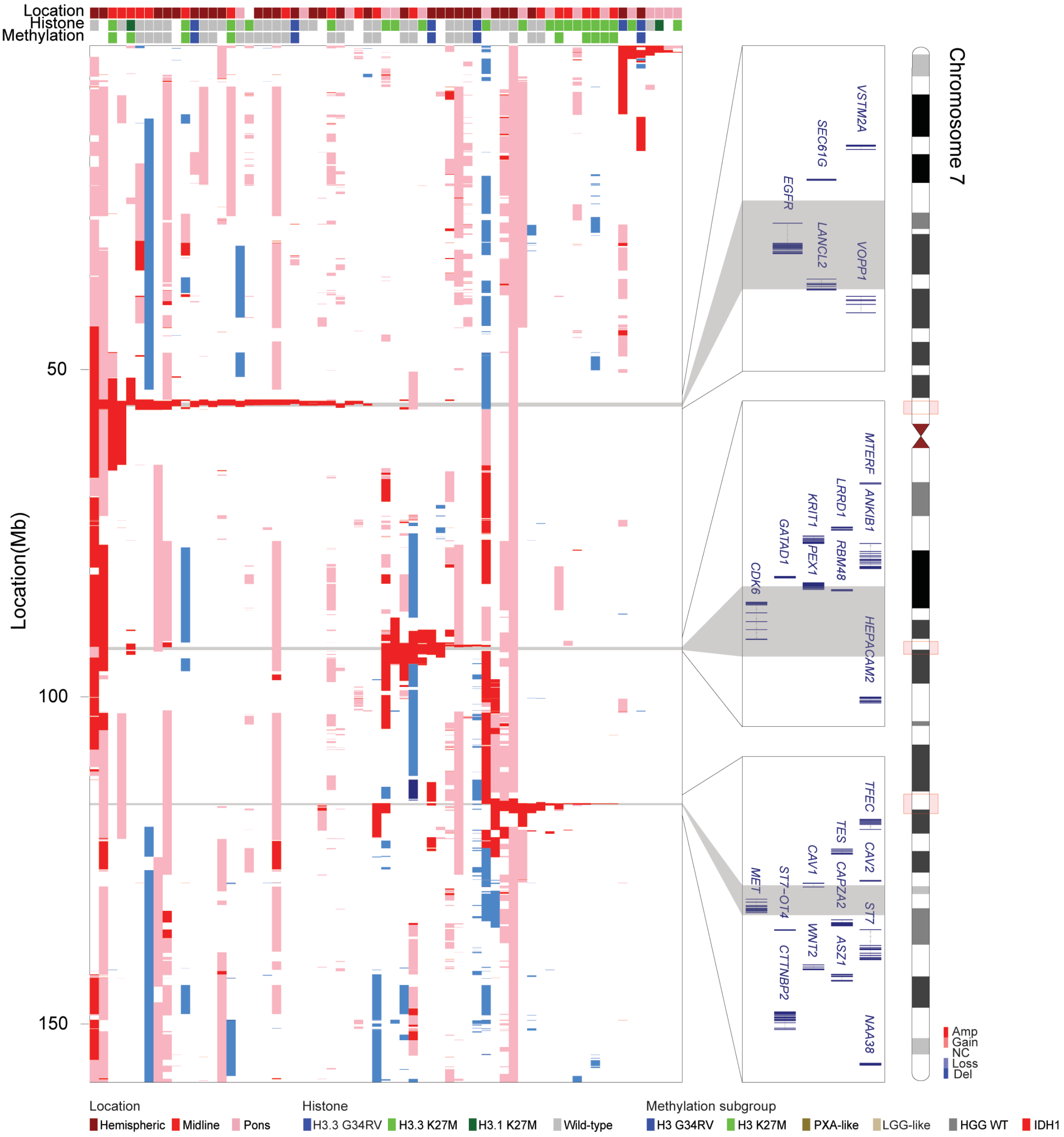

Location
Histone
Methylation
Location(Mb)
50
100
150
Chromosome 7
VSTM2A
SEC61G
EGFR
LANCL2
VOPP1
MTERF
LRRD1
ANKIB1
KRIT1
RBM48
GATAD1
PEX1
CDK6
HEPACAM2
TFEC
TES
CAV2
CAV1
CAPZA2
ST7-OT4
ST7
MET
WNT2
ASZ1
CTTNBP2
NAA38
Amp
Gain
NC
Loss
Del
Location
Hemispheric
Midline
Pons
Histone
H3.3 G34RV
H3.3 K27M
H3.1 K27M
Wild-type
Methylation subgroup
H3 G34RV
H3 K27M
PXA-like
LGG-like
HGG WT
IDH1

b

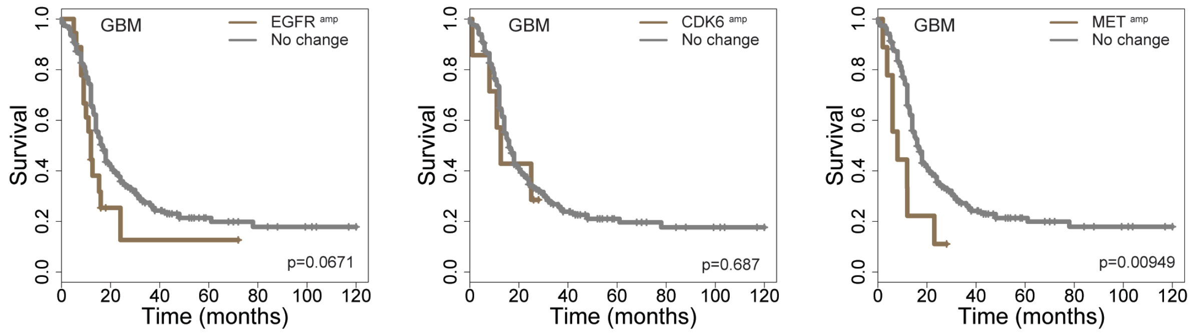

GBM
EGFR amp
No change
p=0.0671
GBM
CDK6 amp
No change
p=0.687
GBM
MET amp
No change
p=0.00949
Survival
Time (months)
0.0
0.2
0.4
0.6
0.8
1.0
0
20
40
60
80
100
120

G

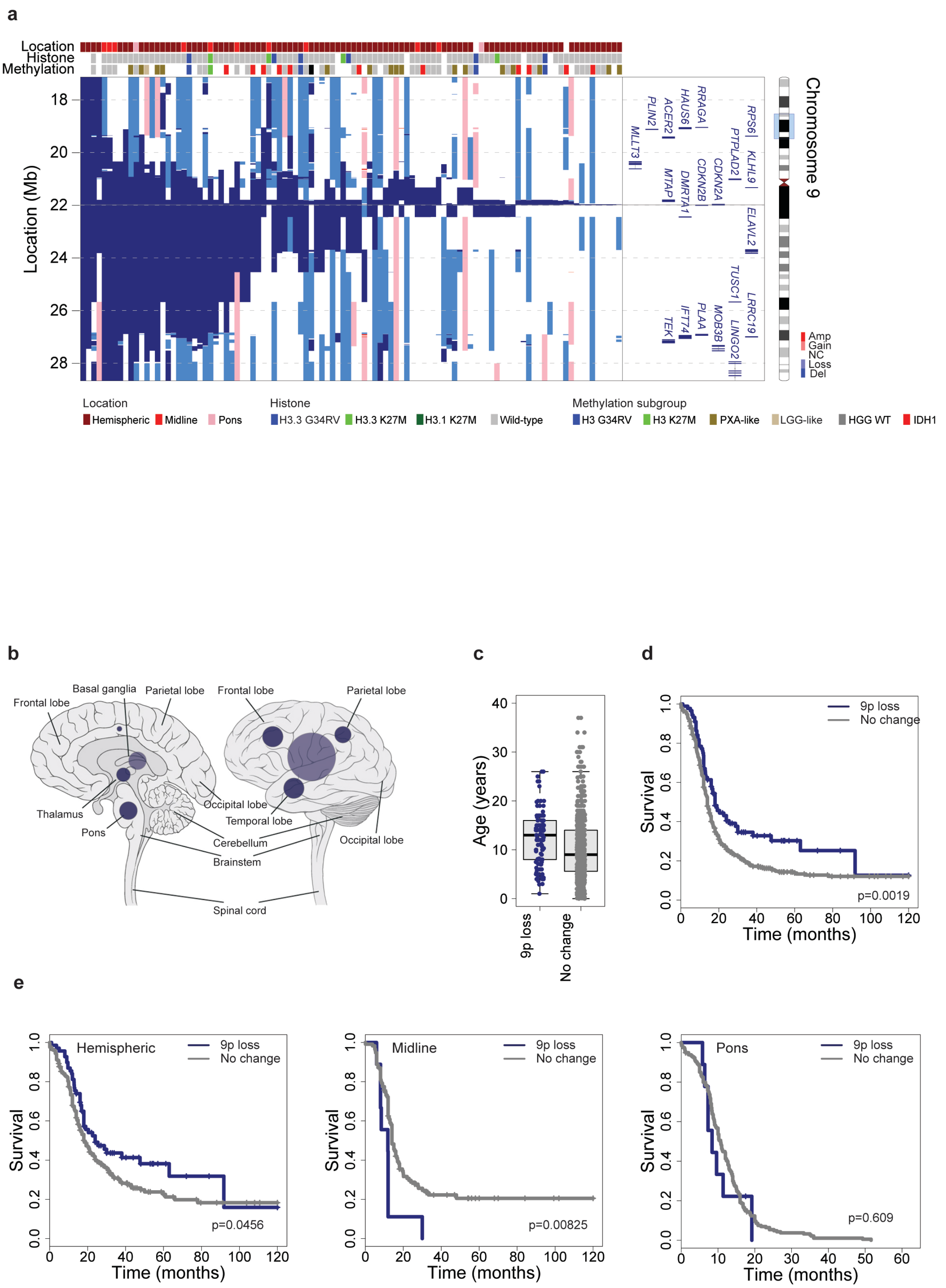
a
Location
Histone
Methylation
Location (Mb)
18
20
22
24
26
28
MLLT3
PLIN2
ACER2
HAUS6
RRAGA
RPS6
PTPLAD2
KLHL9
MTAP
DMRTA1
CDKN2B
CDKN2A
ELAVL2
TUSC1
LRRC19
TEK
IFT74
PLAA
MOB3B
LINGO2
Chromosome 9
Amp
Gain
NC
Loss
Del
Location
Hemispheric
Midline
Pons
Histone
H3.3 G34RV
H3.3 K27M
H3.1 K27M
Wild-type
Methylation subgroup
H3 G34RV
H3 K27M
PXA-like
LGG-like
HGG WT
IDH1
b
Frontal lobe
Basal ganglia
Parietal lobe
Frontal lobe
Parietal lobe
Thalamus
Pons
Occipital lobe
Temporal lobe
Cerebellum
Brainstem
Occipital lobe
Spinal cord
c
Age (years)
9p loss
No change
d
Survival
9p loss
No change
p=0.0019
Time (months)
e
Hemispheric
p=0.0456
Midline
p=0.00825
Pons
p=0.609
Time (months)

Figure S3 (related to Figure 3) – DNA copy number aberrations in pHGG/DIPG. (A) DNA copy neutral cases. (a) Anatomical location (n=147). Radius of circle is proportional to the number of cases. Left – sagittal section showing internal structures; right – external view highlighting cerebral lobes. Pale grey = copy neutral cases. Lighter shaded circles represent a non-specific designation of hemispheric, midline or brainstem. (b) Boxplot showing age at diagnosis of included cases, separated by DNA copy neutral cases (n=798). The thick line within the box is the median, the lower and upper limits of the boxes represent the first and third quartiles, and the whiskers 1.5x the interquartile range. *** $p<0.0001$, t-test. (c) Kaplan-Meier plot of overall survival of cases separated by DNA copy neutral cases, p value calculated by the log-rank test (n=638). (d) Venn diagrams showing platform distribution of copy neutral (left, n=147) and other cases (right, n=687). (B-G) Recurrent copy number changes. (a) Segmented exon-level DNA copy number heatmaps for (B) 17p loss (n=156), (C) 9q gain (n=108), (D) 2p24 amplification (n=42), (E) 4q12 amplification (n=77), (F) chromosome 7 amplifications (n=59), (G) 9p21 deletion (n=102). Dark red, amplification; red, gain; dark blue, deletion; blue, loss. Chromosomal ideograms are provided indicating enlarged genome browser view and genes within common regions targeted across samples (grey). Clinicopathological and molecular annotations are provided as bars according to the included key. (B-F) (b) Kaplan-Meier plot of overall survival of subgroups of cases separated by indicated DNA copy number change, p value calculated by the log-rank test. (G) 9p loss. (b) Anatomical location (n=115). Radius of circle is proportional to the number of cases. Left – sagittal section showing internal structures; right – external view highlighting cerebral lobes. Dark blue = 9p loss cases. Lighter shaded circles represent a non-specific designation of hemispheric, midline or brainstem. (c) Boxplot showing age at diagnosis of included cases, separated by 9p loss cases (n=798). The thick line within the box is the median, the lower and upper limits of the boxes represent the first and third quartiles, and the whiskers 1.5x the interquartile range. *** $p<0.0001$, t-test. (d,e) Kaplan-Meier plot of overall survival of cases separated by 9p loss cases, p value calculated by the log-rank test (n=659).

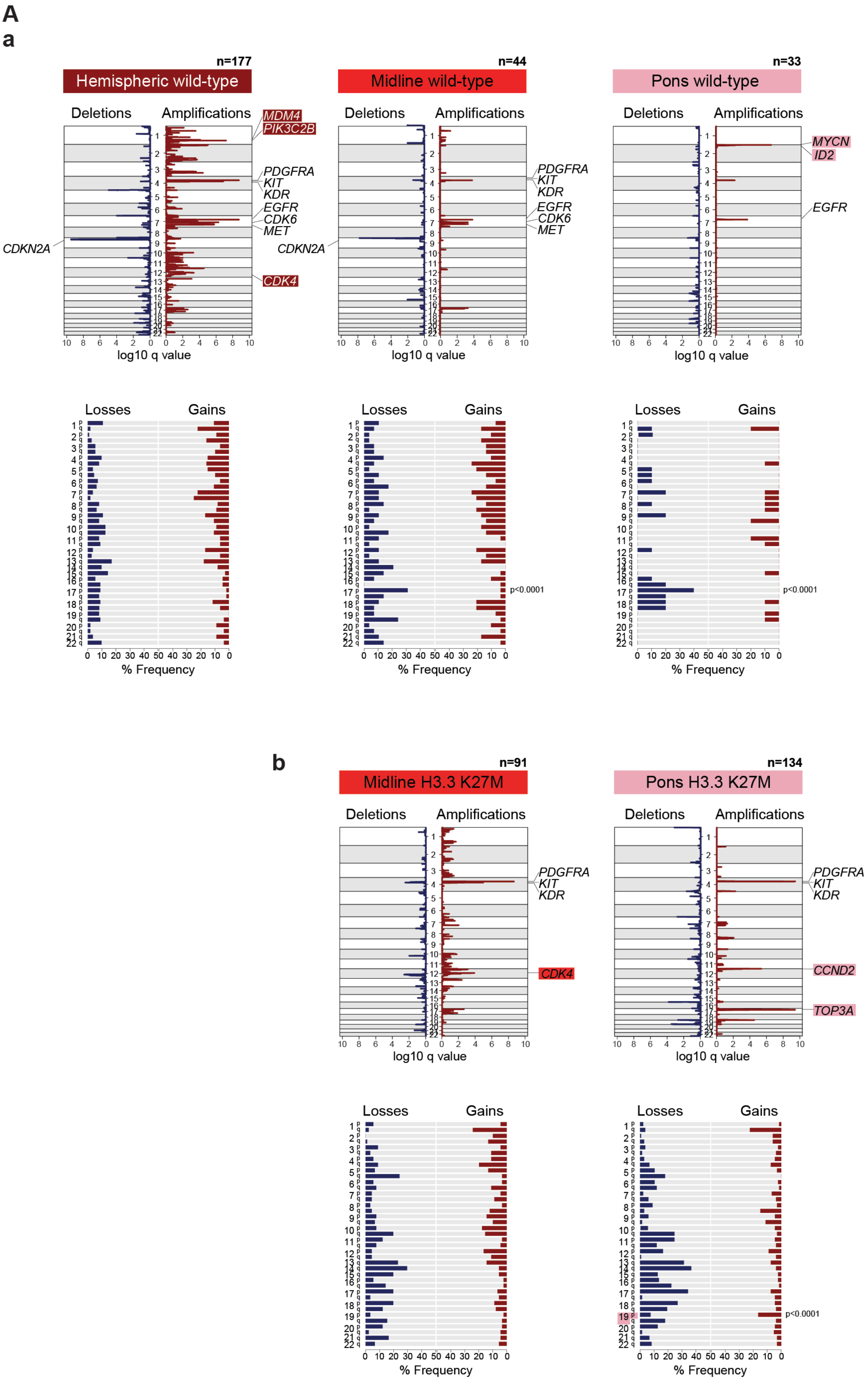

A
a
n=177
Hemispheric wild-type
Deletions
Amplifications
MDM4
PIK3C2B
PDGFRA
KIT
KDR
EGFR
CDK6
MET
CDKN2A
CDK4
log10 q value
Losses
Gains
% Frequency
n=44
Midline wild-type
Deletions
Amplifications
PDGFRA
KIT
KDR
EGFR
CDK6
MET
CDKN2A
log10 q value
Losses
Gains
p<0.0001
% Frequency
n=33
Pons wild-type
Deletions
Amplifications
MYCN
ID2
EGFR
log10 q value
Losses
Gains
p<0.0001
% Frequency
b
n=91
Midline H3.3 K27M
Deletions
Amplifications
PDGFRA
KIT
KDR
CDK4
log10 q value
Losses
Gains
% Frequency
n=134
Pons H3.3 K27M
Deletions
Amplifications
PDGFRA
KIT
KDR
CCND2
TOP3A
log10 q value
Losses
Gains
p<0.0001
% Frequency

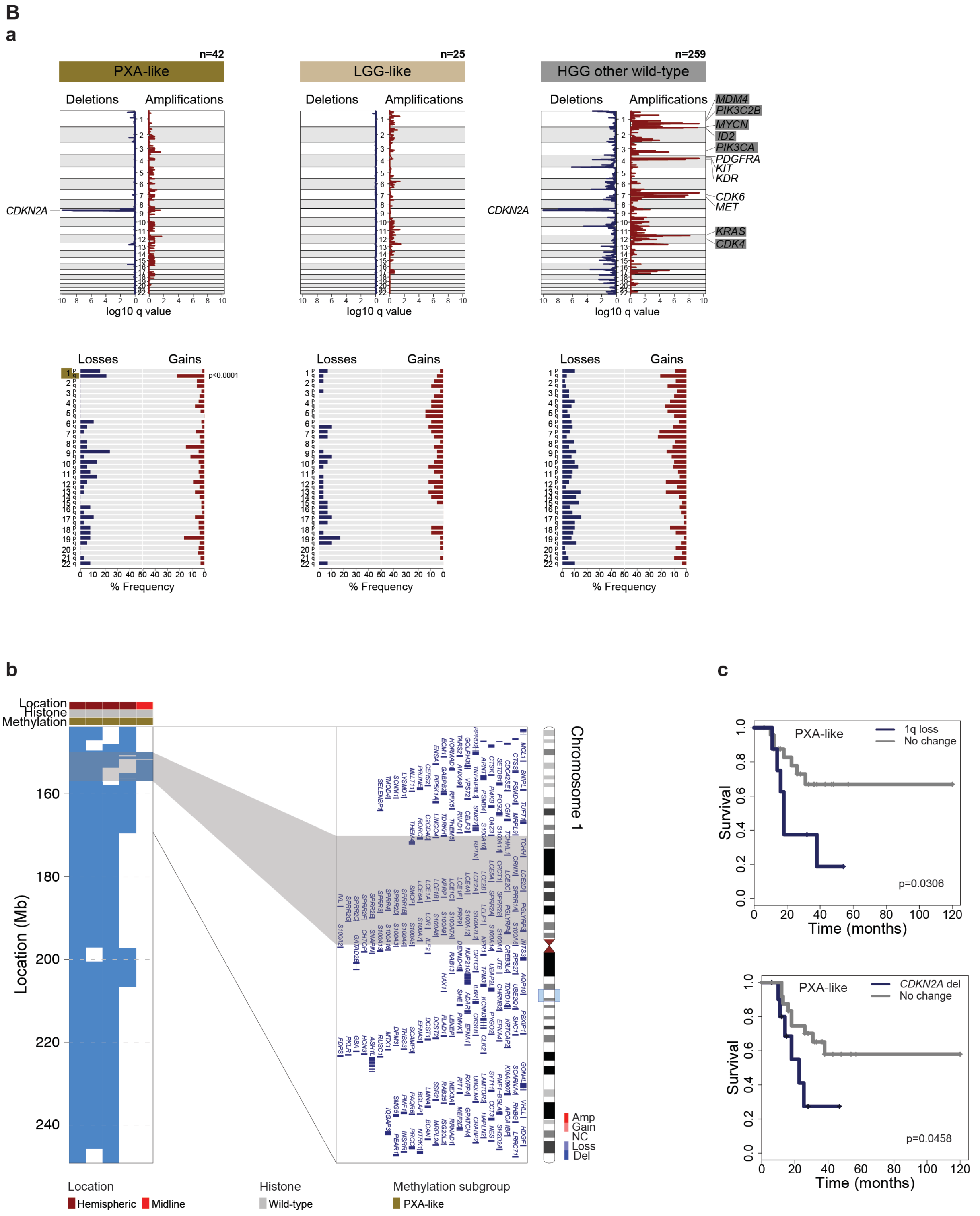

B
a
PXA-like
n=42
LGG-like
n=25
HGG other wild-type
n=259
Deletions
Amplifications
CDKN2A
MDM4
PIK3C2B
MYCN
ID2
PIK3CA
PDGFRA
KIT
KDR
CDK6
MET
KRAS
CDK4
log10 q value
Losses
Gains
p<0.0001
% Frequency
b
Location
Histone
Methylation
Location (Mb)
Chromosome 1
Amp
Gain
NC
Loss
Del
Location
Hemispheric
Midline
Histone
Wild-type
Methylation subgroup
PXA-like
c
PXA-like
1q loss
No change
Survival
Time (months)
p=0.0306
PXA-like
CDKN2A del
No change
Survival
Time (months)
p=0.0458

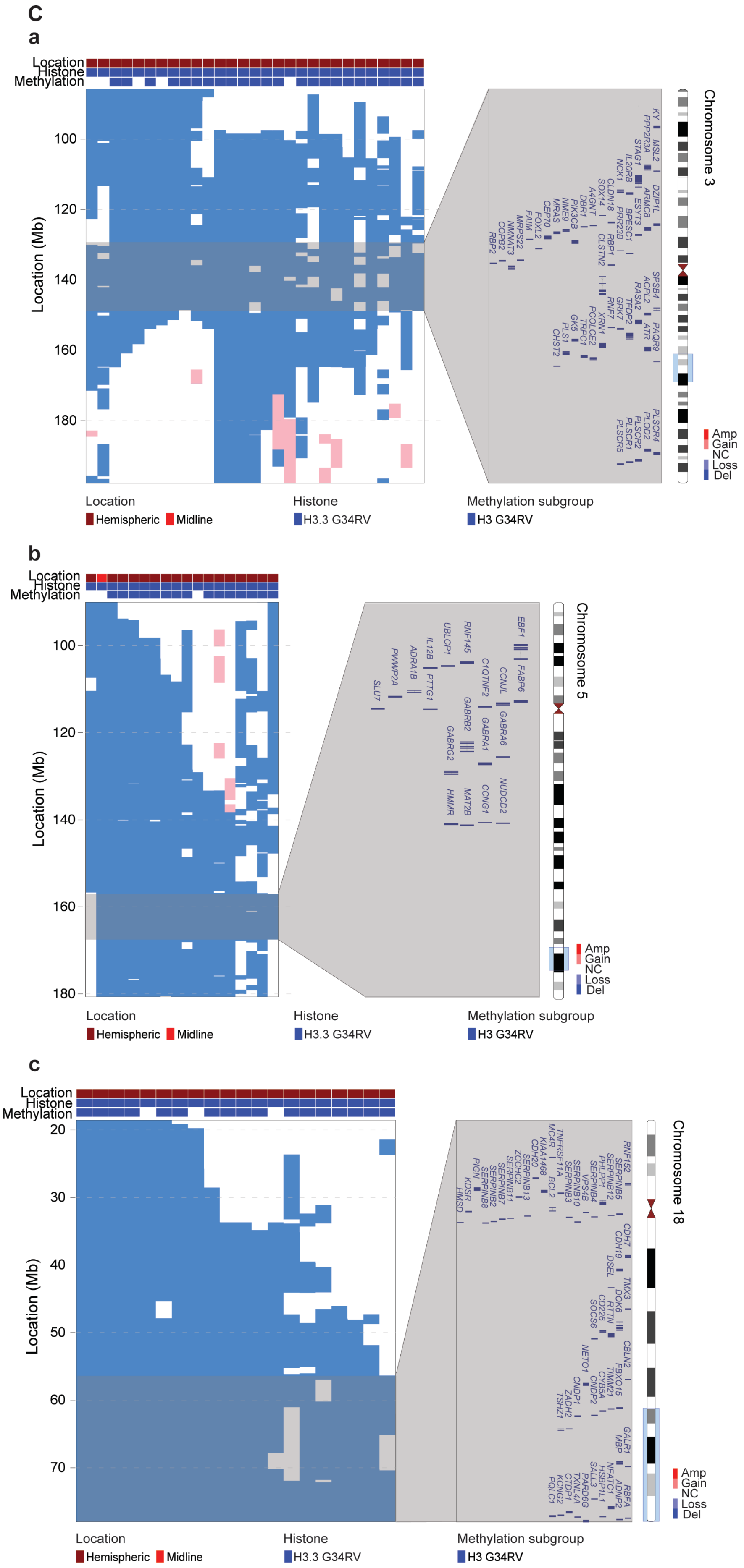
C
a
Location
Histone
Methylation
Location (Mb)
100
120
140
160
180
Chromosome 3
Amp
Gain
NC
Loss
Del
Location
Hemispheric
Midline
Histone
H3.3 G34RV
Methylation subgroup
H3 G34RV
b
Location
Histone
Methylation
Location (Mb)
100
120
140
160
180
Chromosome 5
Amp
Gain
NC
Loss
Del
Location
Hemispheric
Midline
Histone
H3.3 G34RV
Methylation subgroup
H3 G34RV
c
Location
Histone
Methylation
Location (Mb)
20
30
40
50
60
70
Chromosome 18
Amp
Gain
NC
Loss
Del
Location
Hemispheric
Midline
Histone
H3.3 G34RV
Methylation subgroup
H3 G34RV

Figure S4 (related to Figure 4) – Subgroup-specific copy number changes in pHGG/DIPG. (A) GISTIC and frequency barplots for whole chromosomal arm changes for (a) histone wild-type cases and (b) H3.3K27M cases subdivided by anatomical location. Log_{10} values for GISTIC are plotted across the genome for both amplifications (dark red) and deletions (dark blue), with significantly enriched events labelled by likely driver genes. Subgroup-specific genes are highlighted by the appropriate color, as are significantly enriched whole arm alterations ($p<0.0001$, Fishers exact test). (B) Histone wild-type cases. (b) GISTIC and frequency barplots for whole chromosomal arm changes subdivided by simplified methylation subclass. Log_{10} values for GISTIC are plotted across the genome for both amplifications (dark red) and deletions (dark blue), with significantly enriched events labelled by likely driver genes. Subgroup-specific genes are highlighted by the appropriate color, as are significantly enriched whole arm alterations ($p<0.0001$, Fishers exact test). (b) Segmented exon-level DNA copy number heatmaps for 1q loss in PXA-like cases (dark red, amplification; red, gain; dark blue, deletion; blue, loss; n=5). Chromosomal ideograms are provided indicating enlarged genome browser view and genes within common regions targeted across samples (grey). Clinicopathological and molecular annotations are provided as bars according to the included key. (c) Kaplan-Meier plot of overall survival of subgroups of cases separated by indicated DNA copy number change in PXA-like cases (n=35), p value calculated by the log-rank test. (C) Copy number losses in H3.3G34R/V cases. (a) 3q loss (n=29). (b) 5q loss (n=18). (c) 18q loss (n=20). Segmented exon-level DNA copy number heatmaps for losses in H3.3G34R/V cases (dark red, amplification; red, gain; dark blue, deletion; blue, loss). Chromosomal ideograms are provided indicating enlarged genome browser view and genes within common regions targeted across samples (grey). Clinicopathological and molecular annotations are provided as bars according to the included key.

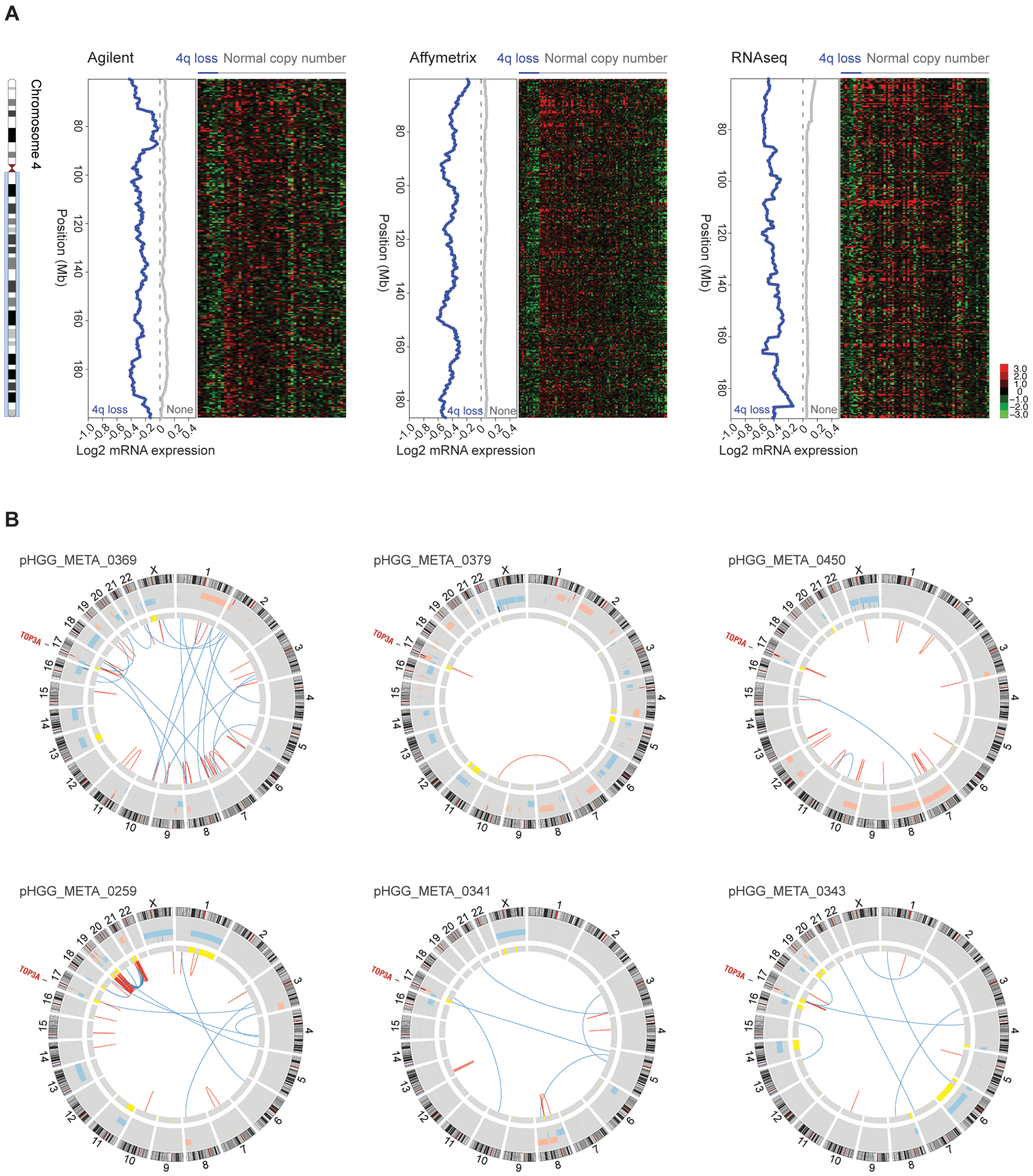
A
Chromosome 4
Agilent
Affymetrix
RNAseq
4q loss
Normal copy number
Position (Mb)
80
100
120
140
160
180
None
-1.0 -0.8 -0.6 -0.4 -0.2 0 0.2 0.4
Log2 mRNA expression
3.0
2.0
1.0
0
-1.0
-2.0
-3.0
B
pHGG_META_0369
pHGG_META_0379
pHGG_META_0450
pHGG_META_0259
pHGG_META_0341
pHGG_META_0343
TOP3A
X
1
2
3
4
5
6
7
8
9
10
11
12
13
14
15
16
17
18
19
20
21
22

Figure S5 (related to Figure 5) – Alterations targeting *FBXW7* in H3.3G34R/V pHGG and *TOP3A* in H3.3K27M DIPG. (A) Gene expression heatmap for all genes on chromosome 4q across three independent platforms, separated by those with 4q loss (blue) and those with normal copy number (grey). Agilent, left (n=67); Affymetrix, middle (n=102); RNAseq, right (n=82). (B) CIRCOS plots for 6 cases (5x H3.3K27M DIPG, 1x midline histone wild-type) with complex rearrangements leading to *TOP3A* amplification. In each case, plots provide DNA copy number changes (dark red, amplification; red, gain; dark blue, deletion; blue, loss) and loss of heterozygosity (yellow) on the inner rings, and intra- (orange) and inter- (blue) chromosomal translocations inside the circle.

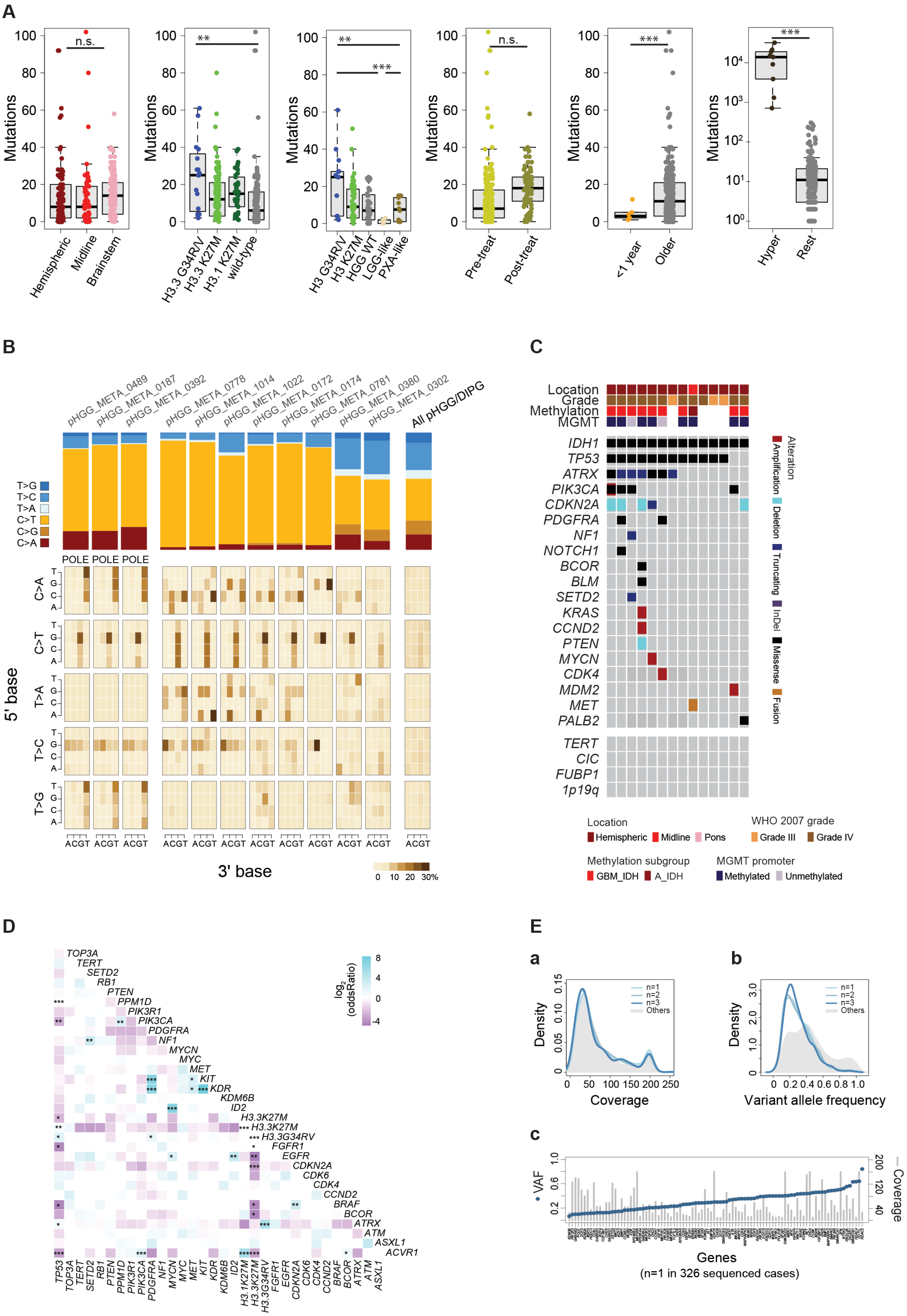

A
Mutations
Hemispheric
Midline
Brainstem
H3.3 G34R/V
H3.3 K27M
H3.1 K27M
wild-type
H3 G34R/V
H3 K27M
HGG WT
LGG-like
PXA-like
Pre-treat
Post-treat
<1 year
Older
Hyper
Rest
n.s.
B
All pHGG/DIPG
POLE
5' base
3' base
C
Location
Grade
Methylation
MGMT
Alteration
Amplification
Deletion
Truncating
InDel
Missense
Fusion
WHO 2007 grade
Grade III
Grade IV
Methylation subgroup
MGMT promoter
Methylated
Unmethylated
D
log2 (oddsRatio)
E
Density
Coverage
Variant allele frequency
VAF
Genes
(n=1 in 326 sequenced cases)

Figure S6 (related to Figure 6) – Somatic mutations in pHGG/DIPG. (A) Mutation rate boxplots across non-hypermutator pHGG/DIPG separated according to location (n=318), histone mutation(n=326), methylation subgroup (n=129), pre/post-treatment sampling (n=326) and age <1 year (n=326), and then compared to 11 hypermutator cases (plotted on a $\log_{10}$ scale). The thick line within the box is the median, the lower and upper limits of the boxes represent the first and third quartiles, and the whiskers 1.5x the interquartile range. *** adjusted p<0.0001, **<0.01, t-test. (B) Mutation signatures for the 11 hypermutator cases. (Top) Simple stacked barplot representation of the proportion of mutation types observed in individual hypermutator cases and the remaining accumulated dataset. Base changes given in the key. (Bottom) Mutation context given for each of the 96 mutated trinucleotides, represented by heatmap. The base located 5′ to each mutated base is shown on the vertical axis, and the 3′ base is on the horizontal axis. (C) Oncoprint representation of an integrated annotation of somatic mutations and DNA copy number changes in *IDH1* mutant cases (n=14). Clinicopathological and molecular annotations are provided as bars according to the included key. (D) Mutual exclusivity analysis. Pairwise Fishers exact tests are calculated for each of the 30 most commonly altered genes in pHGG/DIPG. Log_2-transformed odds ratios are plotted in a purple (negative correlation) to cyan (positive correlation) color scheme. White boxes represent no co-occuring events. *** adjusted p<0.001, ** adjusted p<0.01, *adjusted p<0.05. (E) Coverage and variant allele frequency (VAF) statistics for rare variants. (a) Coverage and (b) VAF are provided as a density plot for those variants detected in 1-3 cases (blue lines) overlaid with the remaining variants (shaded grey). (c) Co-plot of VAF (blue points) and coverage (grey bars) for variants detected in a single case, ordered by VAF.

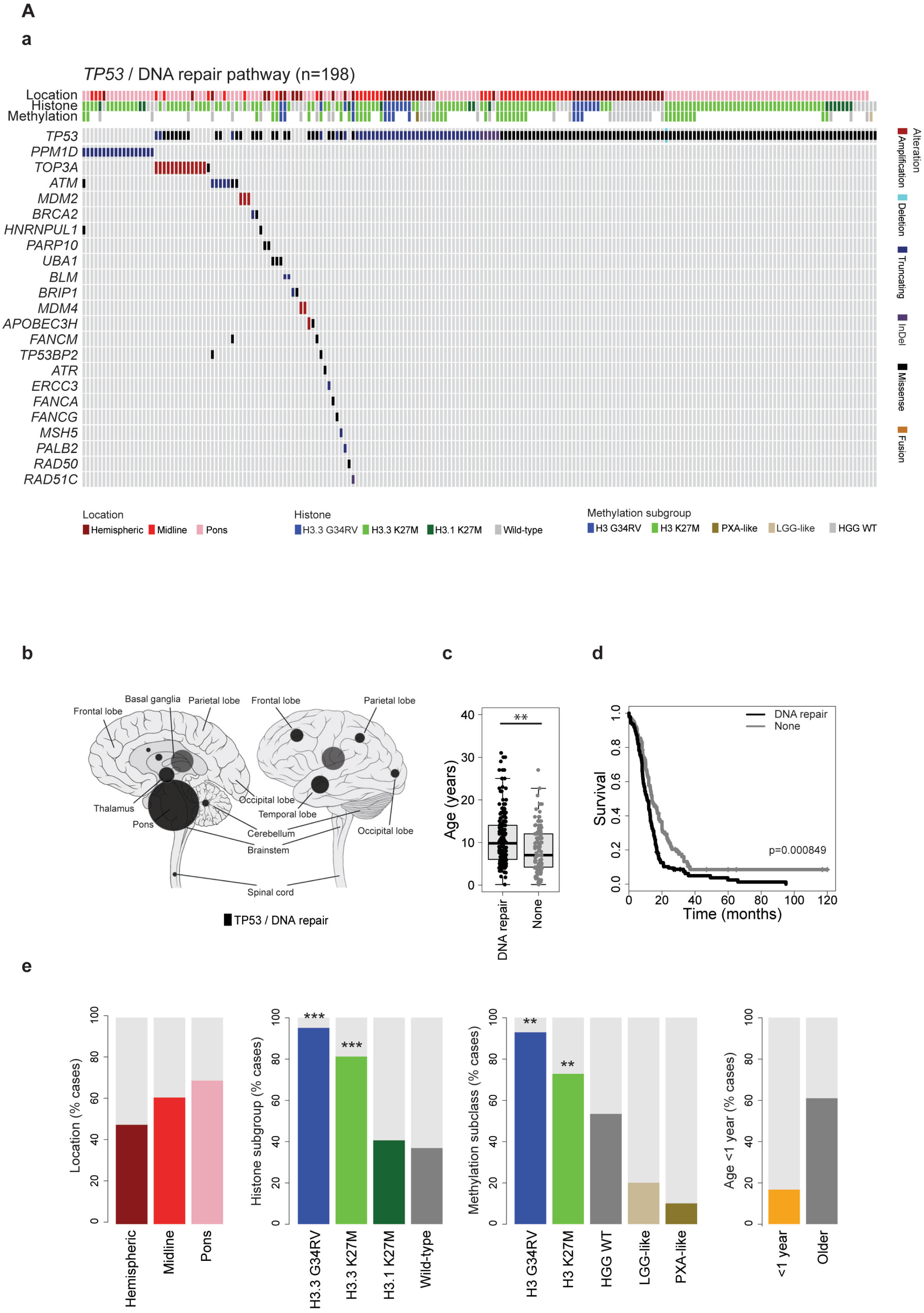
A
a
TP53 / DNA repair pathway (n=198)
Location
Histone
Methylation
TP53
PPM1D
TOP3A
ATM
MDM2
BRCA2
HNRNPUL1
PARP10
UBA1
BLM
BRIP1
MDM4
APOBEC3H
FANCM
TP53BP2
ATR
ERCC3
FANCA
FANCG
MSH5
PALB2
RAD50
RAD51C
Alteration
Amplification
Deletion
Truncating
InDel
Missense
Fusion
Location
Hemispheric
Midline
Pons
Histone
H3.3 G34RV
H3.3 K27M
H3.1 K27M
Wild-type
Methylation subgroup
H3 G34RV
H3 K27M
PXA-like
LGG-like
HGG WT
b
Frontal lobe
Basal ganglia
Parietal lobe
Frontal lobe
Parietal lobe
Thalamus
Pons
Occipital lobe
Temporal lobe
Cerebellum
Brainstem
Occipital lobe
Spinal cord
TP53 / DNA repair
c
Age (years)
**
DNA repair
None
d
Survival
DNA repair
None
p=0.000849
Time (months)
e
Location (% cases)
Hemispheric
Midline
Pons
Histone subgroup (% cases)
***
***
H3.3 G34RV
H3.3 K27M
H3.1 K27M
Wild-type
Methylation subclass (% cases)
**
**
H3 G34RV
H3 K27M
HGG WT
LGG-like
PXA-like
Age <1 year (% cases)
<1 year
Older

B

a

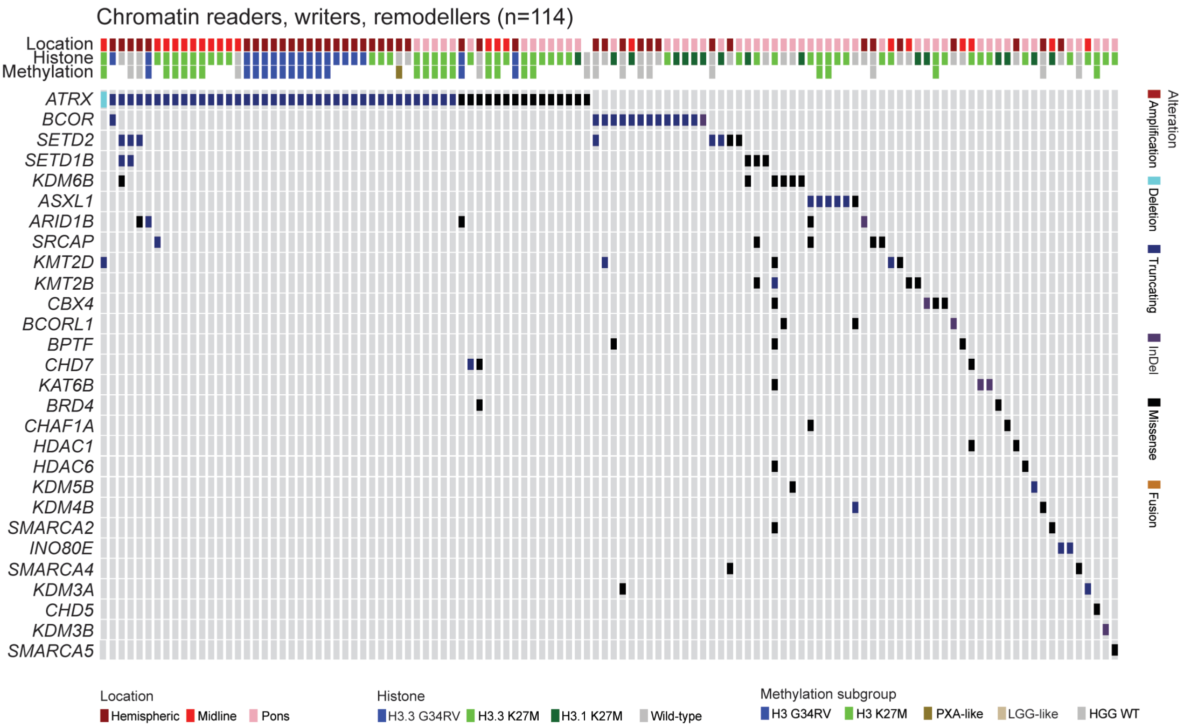
Chromatin readers, writers, remodellers (n=114)
Location
Histone
Methylation
ATRX
BCOR
SETD2
SETD1B
KDM6B
ASXL1
ARID1B
SRCAP
KMT2D
KMT2B
CBX4
BCORL1
BPTF
CHD7
KAT6B
BRD4
CHAF1A
HDAC1
HDAC6
KDM5B
KDM4B
SMARCA2
INO80E
SMARCA4
KDM3A
CHD5
KDM3B
SMARCA5
Alteration
Amplification
Deletion
Truncating
InDel
Missense
Fusion
Location
Hemispheric
Midline
Pons
Histone
H3.3 G34RV
H3.3 K27M
H3.1 K27M
Wild-type
Methylation subgroup
H3 G34RV
H3 K27M
PXA-like
LGG-like
HGG WT

b

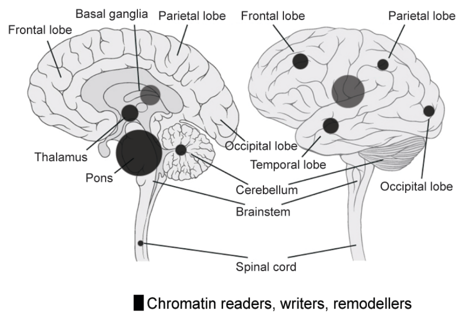
Frontal lobe
Basal ganglia
Parietal lobe
Frontal lobe
Parietal lobe
Thalamus
Pons
Occipital lobe
Temporal lobe
Cerebellum
Brainstem
Occipital lobe
Spinal cord
Chromatin readers, writers, remodellers

c

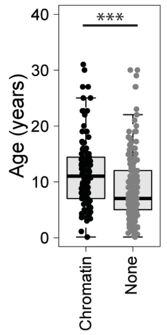
Age (years)
***
0
10
20
30
40
Chromatin
None

d

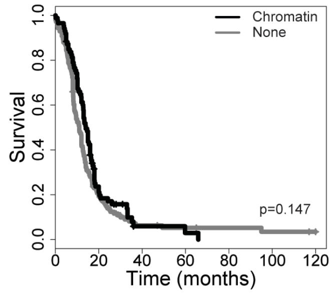
Survival
0.0
0.2
0.4
0.6
0.8
1.0
Chromatin
None
p=0.147
0
20
40
60
80
100
120
Time (months)

e

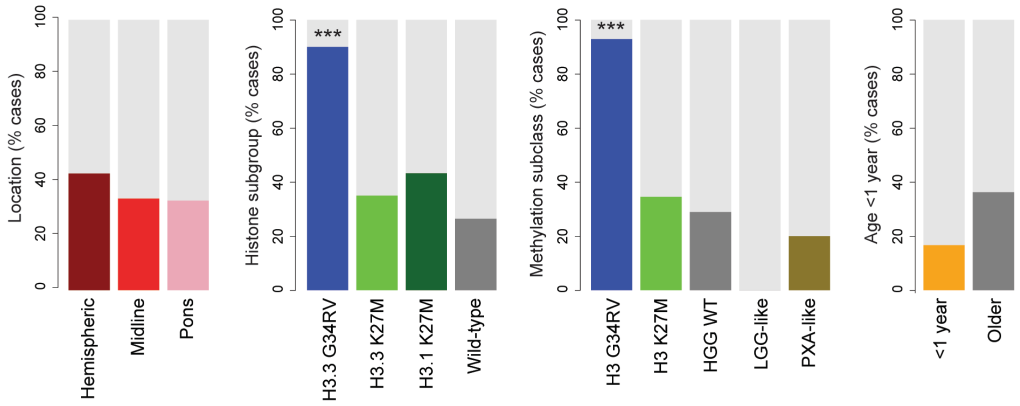
Location (% cases)
Hemispheric
Midline
Pons
Histone subgroup (% cases)
***
H3.3 G34RV
H3.3 K27M
H3.1 K27M
Wild-type
Methylation subclass (% cases)
***
H3 G34RV
H3 K27M
HGG WT
LGG-like
PXA-like
Age <1 year (% cases)
<1 year
Older
0
20
40
60
80
100

C

a

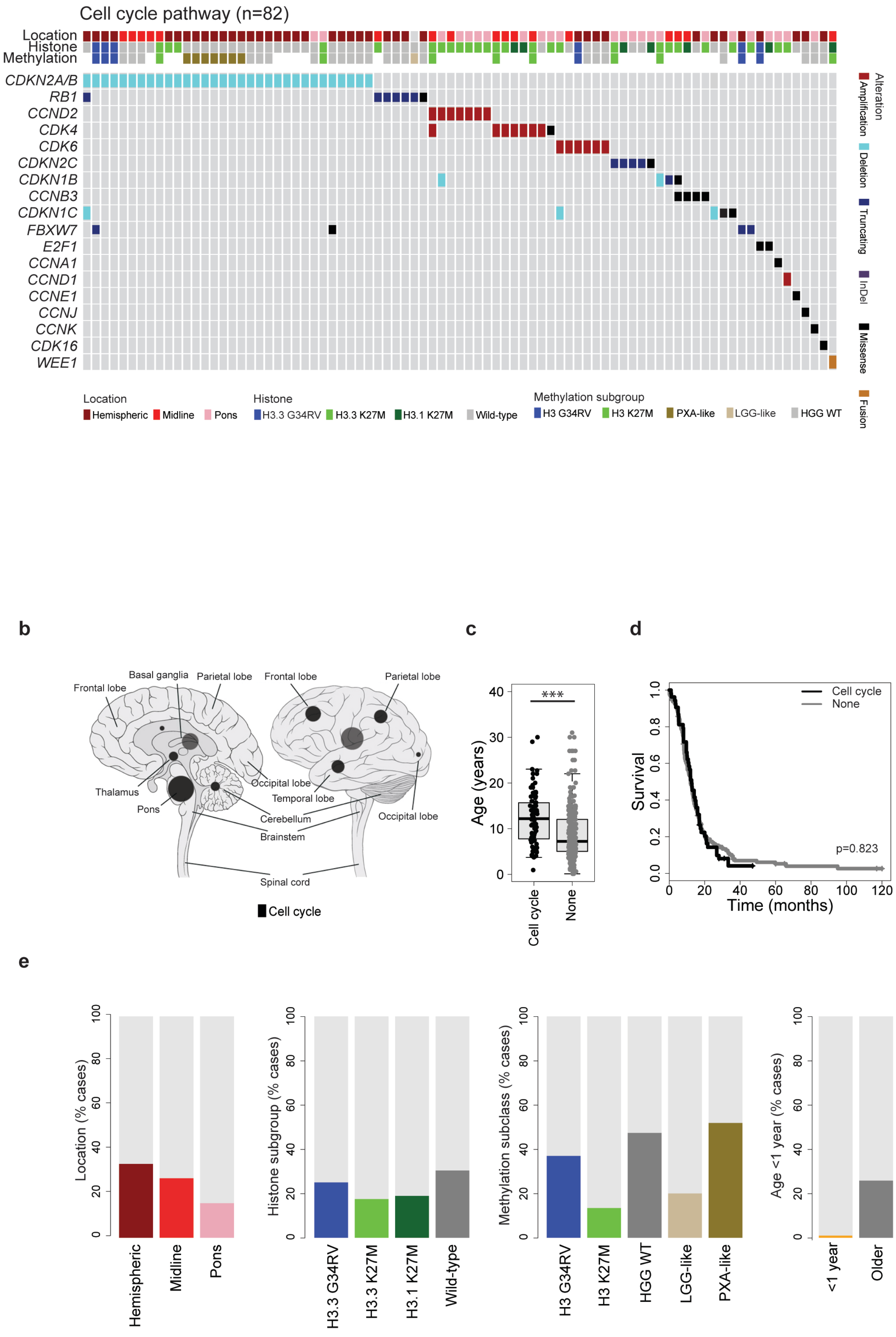

Cell cycle pathway (n=82)
Location
Histone
Methylation
CDKN2A/B
RB1
CCND2
CDK4
CDK6
CDKN2C
CDKN1B
CCNB3
CDKN1C
FBXW7
E2F1
CCNA1
CCND1
CCNE1
CCNJ
CCNK
CDK16
WEE1
Alteration
Amplification
Deletion
Truncating
InDel
Missense
Fusion
Location
Hemispheric
Midline
Pons
Histone
H3.3 G34RV
H3.3 K27M
H3.1 K27M
Wild-type
Methylation subgroup
H3 G34RV
H3 K27M
PXA-like
LGG-like
HGG WT
b
Frontal lobe
Basal ganglia
Parietal lobe
Frontal lobe
Parietal lobe
Thalamus
Pons
Occipital lobe
Temporal lobe
Cerebellum
Occipital lobe
Brainstem
Spinal cord
Cell cycle
c
Age (years)
***
Cell cycle
None
d
Survival
Cell cycle
None
p=0.823
Time (months)
e
Location (% cases)
Hemispheric
Midline
Pons
Histone subgroup (% cases)
H3.3 G34RV
H3.3 K27M
H3.1 K27M
Wild-type
Methylation subclass (% cases)
H3 G34RV
H3 K27M
HGG WT
LGG-like
PXA-like
Age <1 year (% cases)
<1 year
Older

D

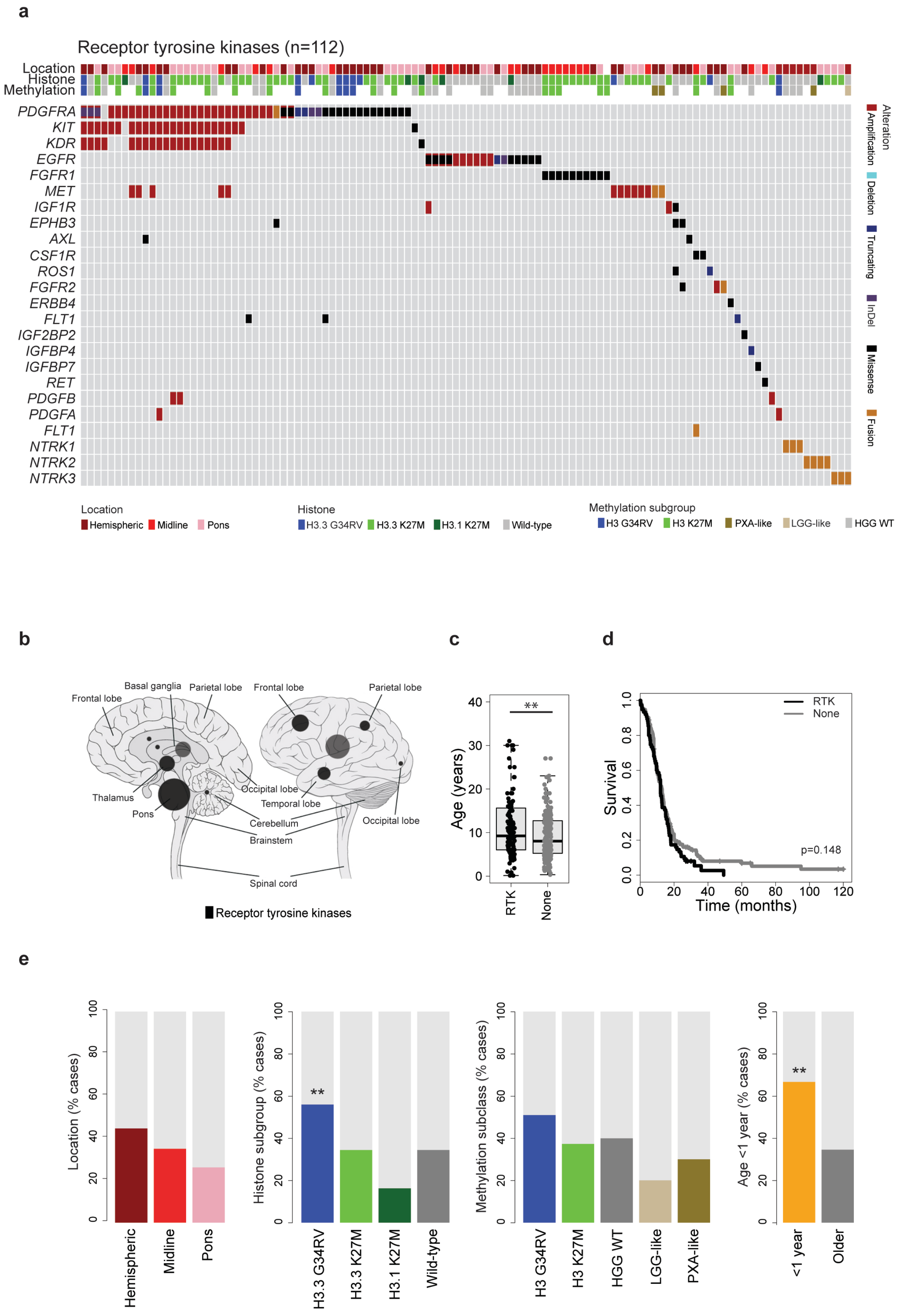
a
Receptor tyrosine kinases (n=112)
Location
Histone
Methylation
PDGFRA
KIT
KDR
EGFR
FGFR1
MET
IGF1R
EPHB3
AXL
CSF1R
ROS1
FGFR2
ERBB4
FLT1
IGF2BP2
IGFBP4
IGFBP7
RET
PDGFB
PDGFA
FLT1
NTRK1
NTRK2
NTRK3
Alteration
Amplification
Deletion
Truncating
InDel
Missense
Fusion
Location
Hemispheric
Midline
Pons
Histone
H3.3 G34RV
H3.3 K27M
H3.1 K27M
Wild-type
Methylation subgroup
H3 G34RV
H3 K27M
PXA-like
LGG-like
HGG WT
b
Frontal lobe
Basal ganglia
Parietal lobe
Frontal lobe
Parietal lobe
Thalamus
Pons
Occipital lobe
Temporal lobe
Cerebellum
Brainstem
Occipital lobe
Spinal cord
Receptor tyrosine kinases
c
Age (years)
**
RTK
None
d
Survival
RTK
None
p=0.148
Time (months)
e
Location (% cases)
Hemispheric
Midline
Pons
Histone subgroup (% cases)
**
H3.3 G34RV
H3.3 K27M
H3.1 K27M
Wild-type
Methylation subclass (% cases)
H3 G34RV
H3 K27M
HGG WT
LGG-like
PXA-like
Age <1 year (% cases)
**
<1 year
Older

E

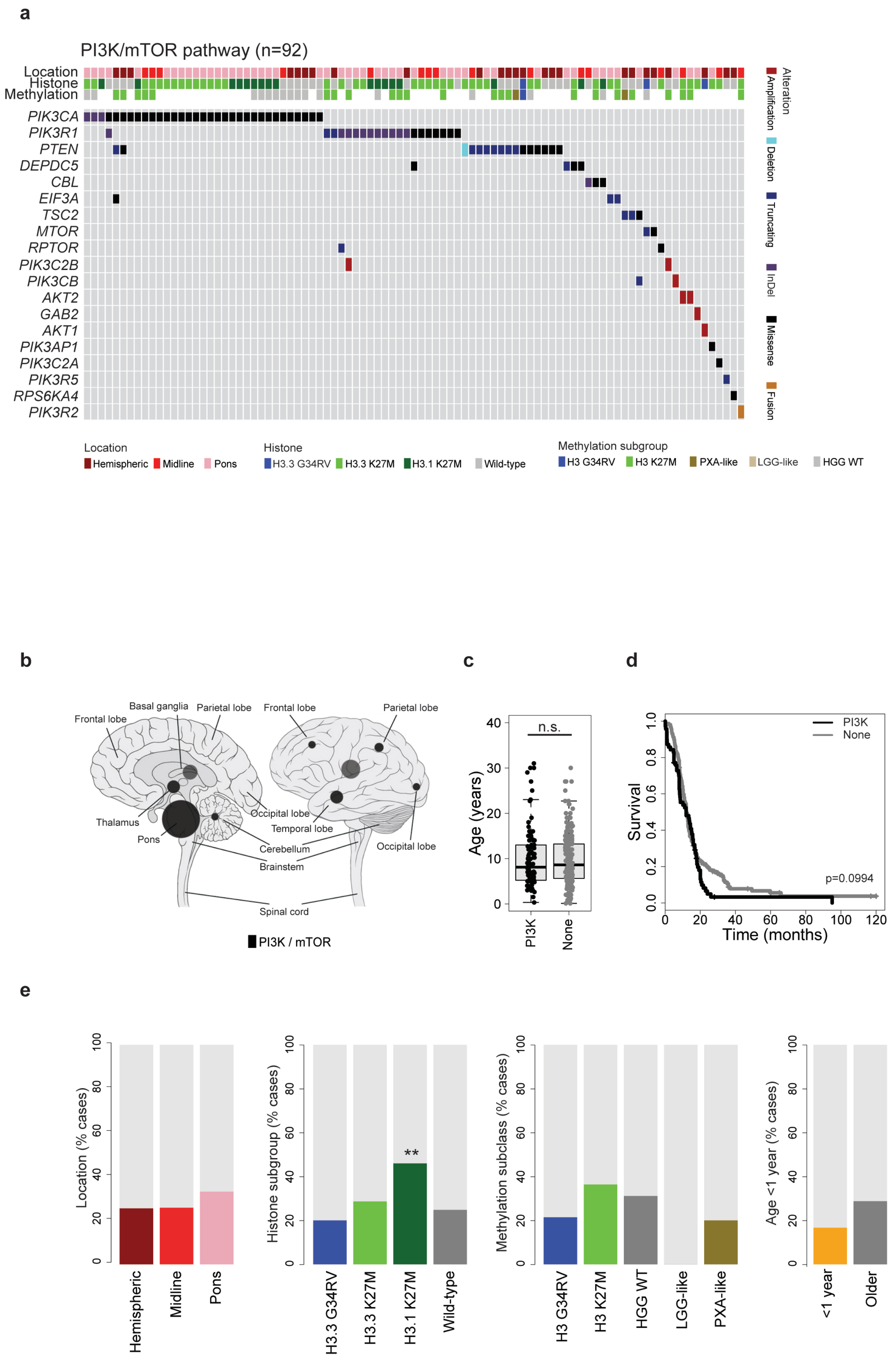

a
PI3K/mTOR pathway (n=92)
Location
Histone
Methylation
PIK3CA
PIK3R1
PTEN
DEPDC5
CBL
EIF3A
TSC2
MTOR
RPTOR
PIK3C2B
PIK3CB
AKT2
GAB2
AKT1
PIK3AP1
PIK3C2A
PIK3R5
RPS6KA4
PIK3R2
Alteration
Amplification
Deletion
Truncating
InDel
Missense
Fusion
Location
Hemispheric
Midline
Pons
Histone
H3.3 G34RV
H3.3 K27M
H3.1 K27M
Wild-type
Methylation subgroup
H3 G34RV
H3 K27M
PXA-like
LGG-like
HGG WT
b
Frontal lobe
Basal ganglia
Parietal lobe
Frontal lobe
Parietal lobe
Thalamus
Pons
Occipital lobe
Temporal lobe
Cerebellum
Brainstem
Occipital lobe
Spinal cord
PI3K / mTOR
c
n.s.
Age (years)
0
10
20
30
40
PI3K
None
d
PI3K
None
Survival
0.0
0.2
0.4
0.6
0.8
1.0
p=0.0994
0
20
40
60
80
100
120
Time (months)
e
Location (% cases)
Hemispheric
Midline
Pons
Histone subgroup (% cases)
**
H3.3 G34RV
H3.3 K27M
H3.1 K27M
Wild-type
Methylation subclass (% cases)
H3 G34RV
H3 K27M
HGG WT
LGG-like
PXA-like
Age <1 year (% cases)
<1 year
Older
0
20
40
60
80
100

F

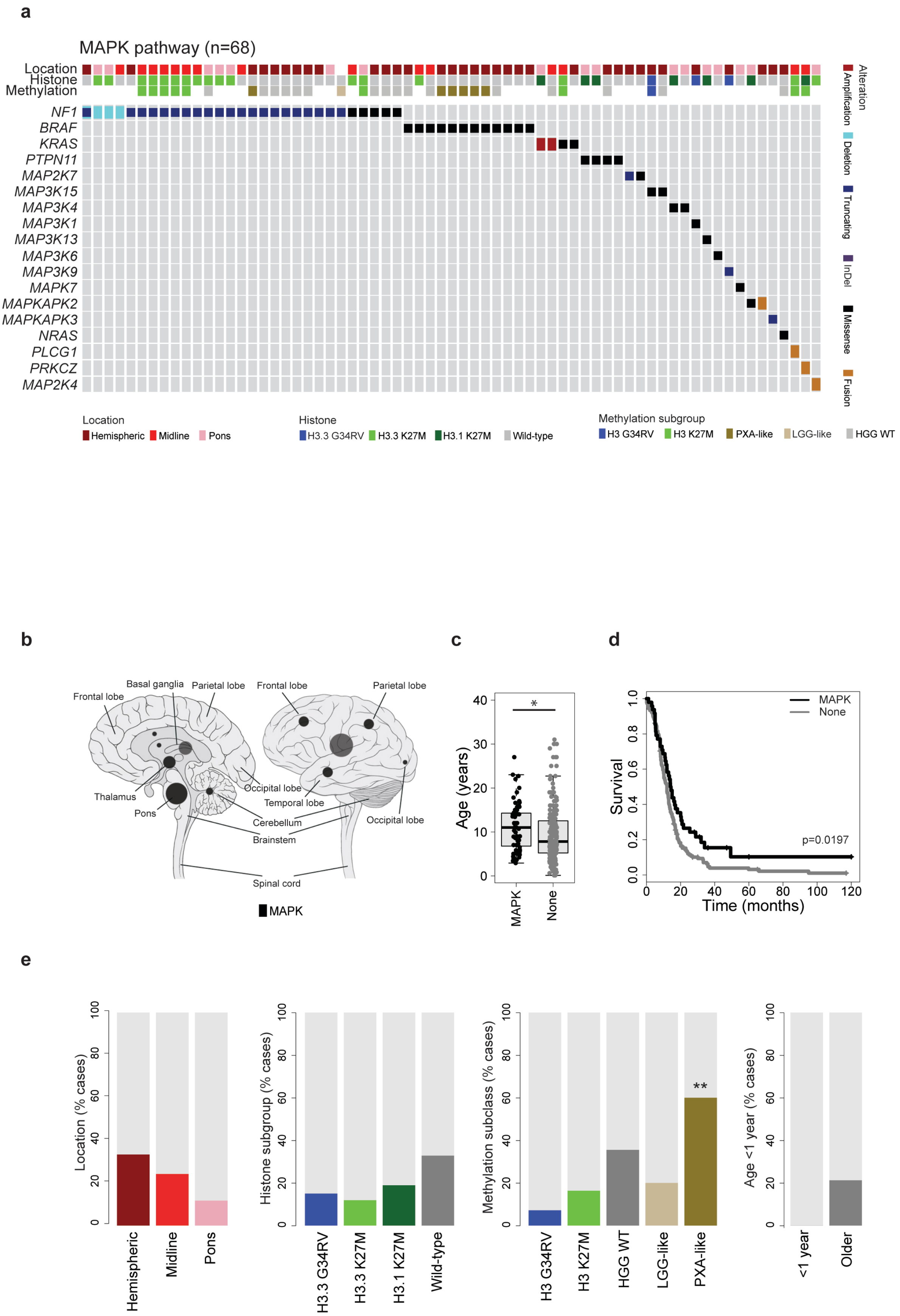
a
MAPK pathway (n=68)
Location
Histone
Methylation
NF1
BRAF
KRAS
PTPN11
MAP2K7
MAP3K15
MAP3K4
MAP3K1
MAP3K13
MAP3K6
MAP3K9
MAPK7
MAPKAPK2
MAPKAPK3
NRAS
PLCG1
PRKCZ
MAP2K4
Alteration
Amplification
Deletion
Truncating
InDel
Missense
Fusion
Location
Hemispheric
Midline
Pons
Histone
H3.3 G34RV
H3.3 K27M
H3.1 K27M
Wild-type
Methylation subgroup
H3 G34RV
H3 K27M
PXA-like
LGG-like
HGG WT
b
Basal ganglia
Parietal lobe
Frontal lobe
Thalamus
Pons
Occipital lobe
Temporal lobe
Cerebellum
Brainstem
Spinal cord
MAPK
c
Age (years)
MAPK
None
d
Survival
Time (months)
p=0.0197
e
Location (% cases)
Hemispheric
Midline
Pons
Histone subgroup (% cases)
H3.3 G34RV
H3.3 K27M
H3.1 K27M
Wild-type
Methylation subclass (% cases)
H3 G34RV
H3 K27M
HGG WT
LGG-like
PXA-like
Age <1 year (% cases)
<1 year
Older

G

a

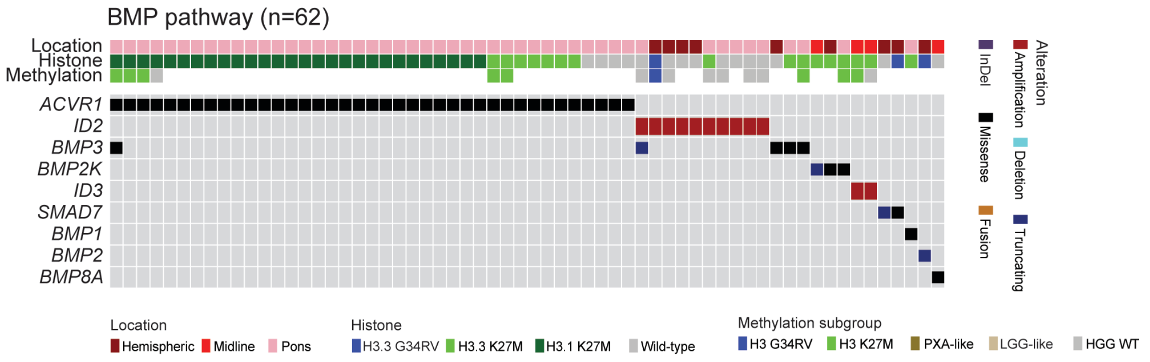

BMP pathway (n=62)
Location
Histone
Methylation
ACVR1
ID2
BMP3
BMP2K
ID3
SMAD7
BMP1
BMP2
BMP8A
Alteration
Amplification
Deletion
Truncating
InDel
Missense
Fusion
Location
Hemispheric
Midline
Pons
Histone
H3.3 G34RV
H3.3 K27M
H3.1 K27M
Wild-type
Methylation subgroup
H3 G34RV
H3 K27M
PXA-like
LGG-like
HGG WT

b

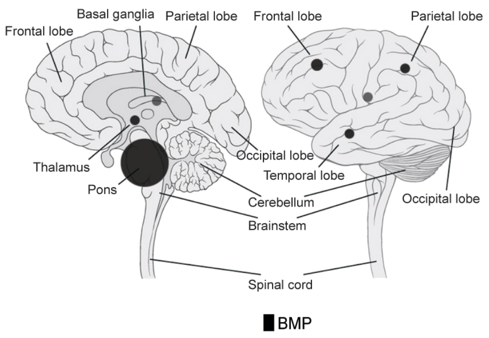

Basal ganglia
Parietal lobe
Frontal lobe
Frontal lobe
Parietal lobe
Thalamus
Pons
Occipital lobe
Temporal lobe
Cerebellum
Brainstem
Occipital lobe
Spinal cord
BMP

c

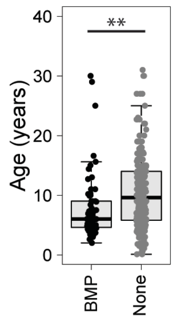

Age (years)
**
BMP
None

d

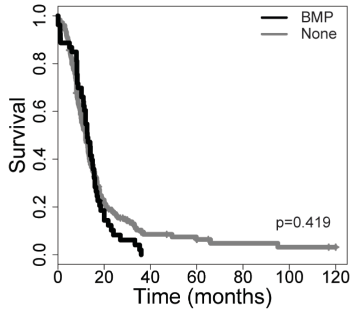

BMP
None
Survival
p=0.419
Time (months)

e

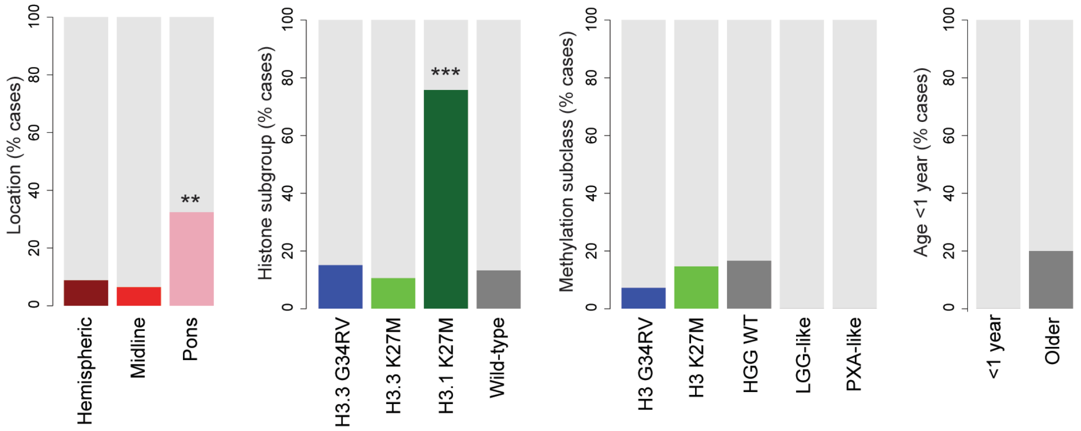

Location (% cases)
**
Hemispheric
Midline
Pons
Histone subgroup (% cases)
***
H3.3 G34RV
H3.3 K27M
H3.1 K27M
Wild-type
Methylation subclass (% cases)
H3 G34RV
H3 K27M
HGG WT
LGG-like
PXA-like
Age <1 year (% cases)
<1 year
Older

H

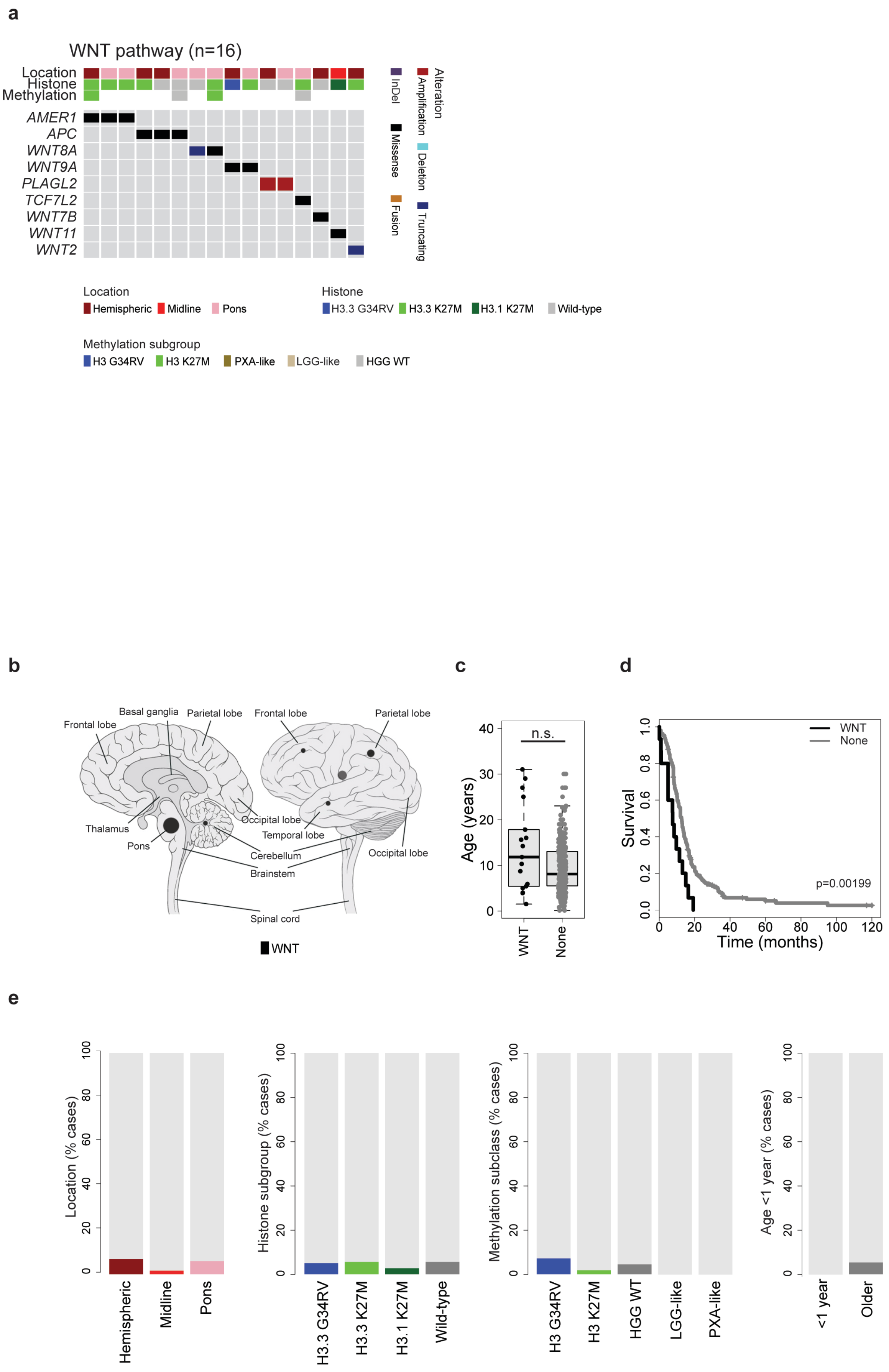
a
WNT pathway (n=16)
Location
Histone
Methylation
AMER1
APC
WNT8A
WNT9A
PLAGL2
TCF7L2
WNT7B
WNT11
WNT2
Alteration
InDel
Amplification
Missense
Deletion
Fusion
Truncating
Location
Hemispheric
Midline
Pons
Histone
H3.3 G34RV
H3.3 K27M
H3.1 K27M
Wild-type
Methylation subgroup
H3 G34RV
H3 K27M
PXA-like
LGG-like
HGG WT
b
Frontal lobe
Basal ganglia
Parietal lobe
Frontal lobe
Parietal lobe
Thalamus
Pons
Occipital lobe
Temporal lobe
Cerebellum
Brainstem
Occipital lobe
Spinal cord
WNT
c
n.s.
Age (years)
WNT
None
d
WNT
None
Survival
p=0.00199
Time (months)
e
Location (% cases)
Hemispheric
Midline
Pons
Histone subgroup (% cases)
H3.3 G34RV
H3.3 K27M
H3.1 K27M
Wild-type
Methylation subclass (% cases)
H3 G34RV
H3 K27M
HGG WT
LGG-like
PXA-like
Age <1 year (% cases)
<1 year
Older

l

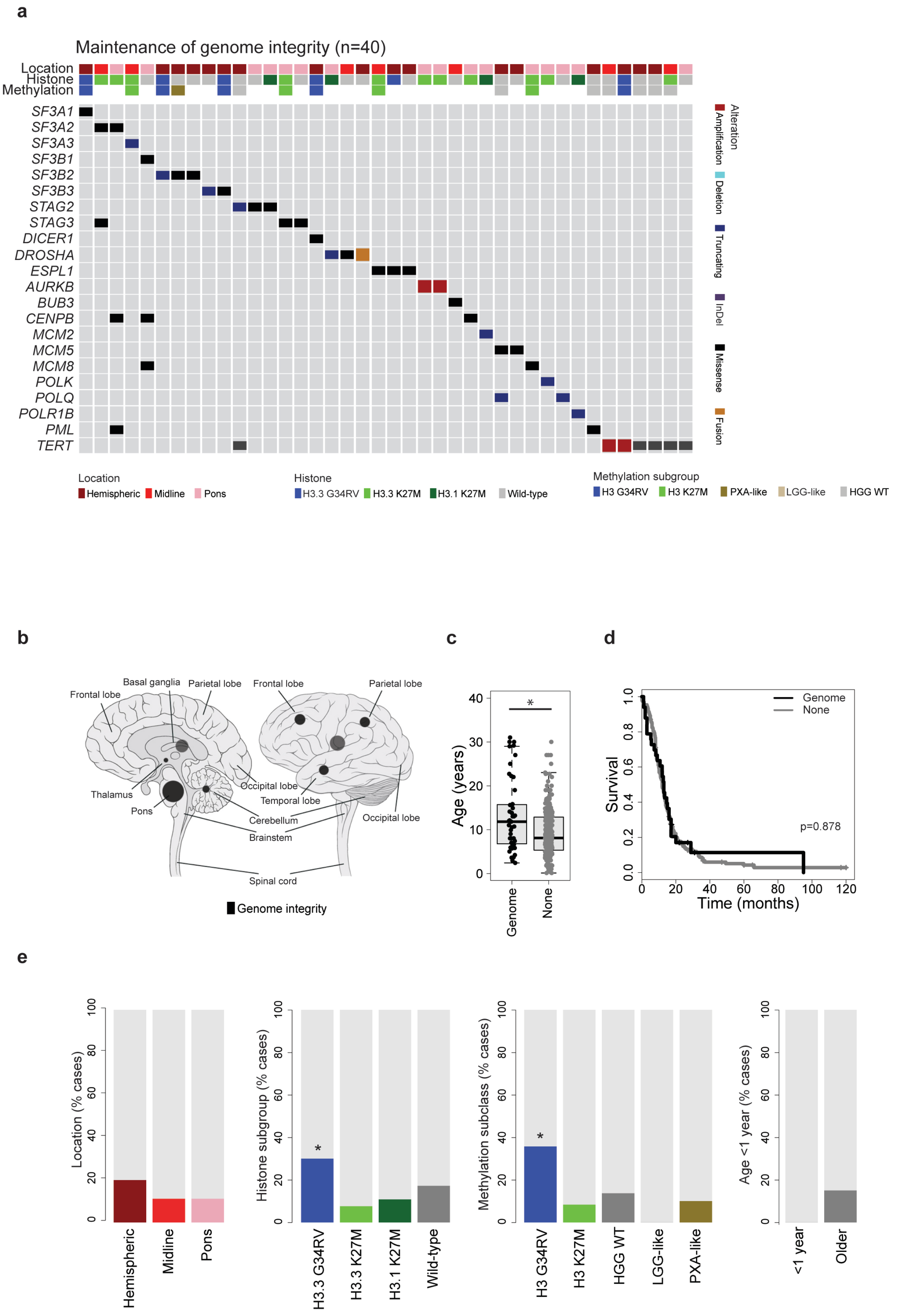
a
Maintenance of genome integrity (n=40)
Location
Histone
Methylation
SF3A1
SF3A2
SF3A3
SF3B1
SF3B2
SF3B3
STAG2
STAG3
DICER1
DROSHA
ESPL1
AURKB
BUB3
CENPB
MCM2
MCM5
MCM8
POLK
POLQ
POLR1B
PML
TERT
Alteration
Amplification
Deletion
Truncating
InDel
Missense
Fusion
Location
Hemispheric
Midline
Pons
Histone
H3.3 G34RV
H3.3 K27M
H3.1 K27M
Wild-type
Methylation subgroup
H3 G34RV
H3 K27M
PXA-like
LGG-like
HGG WT
b
Frontal lobe
Basal ganglia
Parietal lobe
Frontal lobe
Parietal lobe
Thalamus
Pons
Occipital lobe
Temporal lobe
Cerebellum
Occipital lobe
Brainstem
Spinal cord
Genome integrity
c
Age (years)
Genome
None
d
Survival
Time (months)
Genome
None
p=0.878
e
Location (% cases)
Hemispheric
Midline
Pons
Histone subgroup (% cases)
H3.3 G34RV
H3.3 K27M
H3.1 K27M
Wild-type
Methylation subclass (% cases)
H3 G34RV
H3 K27M
HGG WT
LGG-like
PXA-like
Age <1 year (% cases)
<1 year
Older

Figure S7 (related to Figure 7) – Integrated pathway analysis of pHGG/DIPG. (A) TP53 / DNA repair pathway, (B) chromatin readers, writers, remodellers, (C) cell cycle pathway, (D) receptor tyrosine kinases, (E) PI3K/mTOR pathway, (F) MAPK pathway, (G) BMP pathway, (H) WNT pathway and (I) maintenance of genome integrity. (a) Oncoprint representation of an integrated annotation of somatic mutations and DNA copy number changes in cases targeting one or more members of a specific pathway. Clinicopathological and molecular annotations are provided as bars according to the included key. (b) Anatomical location of pathway-targeted cases. Radius of circle is proportional to the number of cases. Left – sagittal section showing internal structures; right – external view highlighting cerebral lobes. Black = given pathway alteration. Lighter shaded circles represent a non-specific designation of hemispheric, midline or brainstem. (c) Boxplot showing age at diagnosis of included cases, separated by pathway-targeted cases. The thick line within the box is the median, the lower and upper limits of the boxes represent the first and third quartiles, and the whiskers 1.5x the interquartile range. (d) Kaplan-Meier plot of overall survival of cases separated by pathway-targeted cases, p value calculated by the log-rank test. (e) Barplots showing distribution of pathway-targeted cases by anatomical location, histone subgroup, methylation subgroup and age. *** $p<0.001$, ** $p<0.01$, *$p<0.05$, n.s., not significant, t-test.

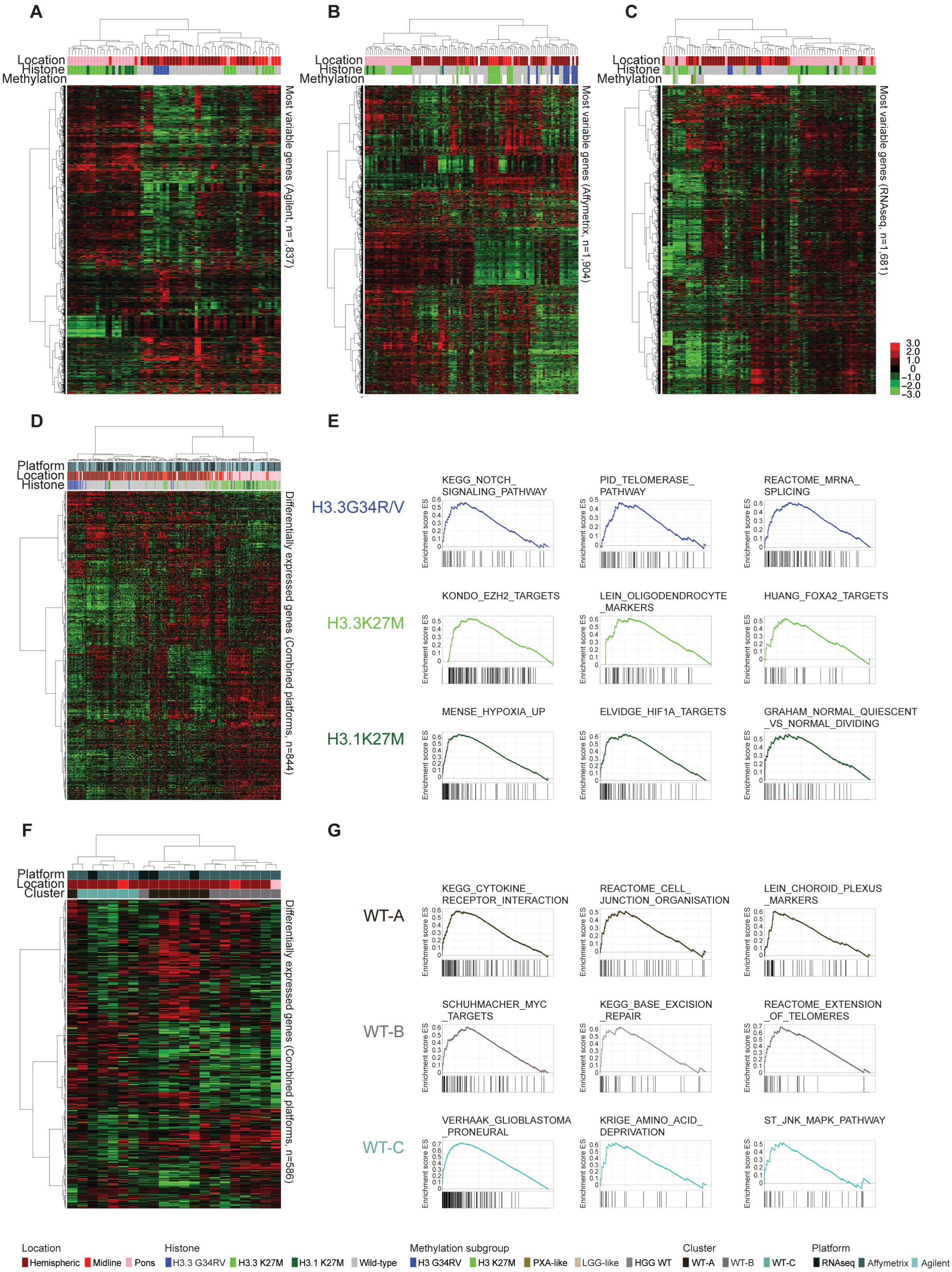

A
B
C
Location
Histone
Methylation
Most variable genes (Agilent, n=1,837)
Most variable genes (Affymetrix, n=1,904)
Most variable genes (RNAseq, n=1,681)
3.0
2.0
1.0
0
−1.0
−2.0
−3.0
D
E
Platform
Location
Histone
Differentially expressed genes (Combined platforms, n=844)
H3.3G34R/V
KEGG_NOTCH_SIGNALING_PATHWAY
PID_TELOMERASE_PATHWAY
REACTOME_MRNA_SPLICING
H3.3K27M
KONDO_EZH2_TARGETS
LEIN_OLIGODENDROCYTE_MARKERS
HUANG_FOXA2_TARGETS
H3.1K27M
MENSE_HYPOXIA_UP
ELVIDGE_HIF1A_TARGETS
GRAHAM_NORMAL_QUIESCENT_VS_NORMAL_DIVIDING
Enrichment score ES
F
G
Platform
Location
Cluster
Differentially expressed genes (Combined platforms, n=586)
WT-A
KEGG_CYTOKINE_RECEPTOR_INTERACTION
REACTOME_CELL_JUNCTION_ORGANISATION
LEIN_CHOROID_PLEXUS_MARKERS
WT-B
SCHUHMACHER_MYC_TARGETS
KEGG_BASE_EXCISION_REPAIR
REACTOME_EXTENSION_OF_TELOMERES
WT-C
VERHAAK_GLIOBLASTOMA_PRONEURAL
KRIGE_AMINO_ACID_DEPRIVATION
ST_JNK_MAPK_PATHWAY
Location
Hemispheric
Midline
Pons
Histone
H3.3 G34RV
H3.3 K27M
H3.1 K27M
Wild-type
Methylation subgroup
H3 G34RV
H3 K27M
PXA-like
LGG-like
HGG WT
Cluster
WT-A
WT-B
WT-C
Platform
RNAseq
Affymetrix
Agilent

Figure S8 (related to Figure 8) – Integrated analysis of H3/IDH1 wild-type pHGG/DIPG. (A-C) Unsupervised hierarchical clustering of the most variable genes in samples profiled by (A) Agilent arrays (n=67), (B) Affymetrix arrays (n=102) and/or (C) RNA sequencing (n=82). (D) A combined dataset across all platforms (n=220) is subjected to differential expression analysis for H3.3G34R/V, H3.3K27M and H3.1K27M mutations, with significant genes plotted in the heatmap. (E) Gene set enrichment analysis for selected significant gene sets for each comparison. (F) Combined dataset for H3/IDH1 wild-type clusters WT-A, WT-B and WT-C (n=21), with significantly differentially expressed genes plotted in the heatmap. (G) Gene set enrichment analysis for selected significant gene sets for each comparison. Clinicopathological and molecular annotations are provided as bars according to the included key.
