# Supplementary material for: Integrated Molecular Meta-Analysis of 1,000 Pediatric High-Grade and Diffuse Intrinsic Pontine Glioma
Source: Cancer Cell. 2017 Oct 9;32(4):520–537.e5. doi: 10.1016/j.ccell.2017.08.017 (PMC5637314; doi:10.1016/j.ccell.2017.08.017)
Supplement: Document S2. Article plus Supplemental Information [file mmc10.pdf]

Article

Cancer Cell

Integrated Molecular Meta-Analysis of 1,000 Pediatric High-Grade and Diffuse Intrinsic Pontine Glioma

Graphical Abstract

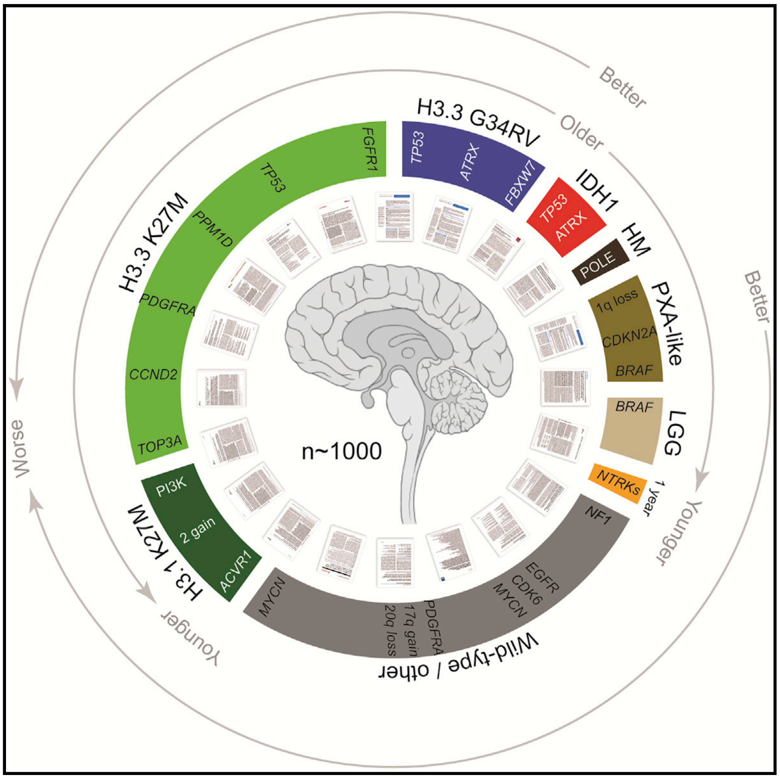

Authors

Alan Mackay, Anna Burford, Diana Carvalho, ..., Michael Baudis, Adam Resnick, Chris Jones

Correspondence

chris.jones@icr.ac.uk

In Brief

Mackay et al. perform an integrated analysis of >1,000 cases of pediatric high-grade glioma and diffuse intrinsic pontine glioma. They identify co-segregating mutations in histone-mutant subgroups and show that histone wild-type subgroups are molecularly more similar to lower-grade tumors.

Highlights

- Pediatric HGG and DIPG comprise a diverse set of clinical and biological subgroups
- Somatic coding mutations per tumor range from none to among the highest seen in human cancer
- Histone mutations co-segregate with distinct alterations and downstream pathways
- H3/IDH1 WT tumors may resemble low-grade lesions and have targetable alterations

Mackay et al., 2017, Cancer Cell *32*, 520–537
October 9, 2017 © 2017 The Author(s). Published by Elsevier Inc.
http://dx.doi.org/10.1016/j.ccell.2017.08.017

Cancer Cell
Article

Integrated Molecular Meta-Analysis of 1,000 Pediatric High-Grade and Diffuse Intrinsic Pontine Glioma

Alan Mackay,[1,2] Anna Burford,[1,2] Diana Carvalho,[1,2] Elisa Izquierdo,[1,2] Janat Fazal-Salom,[1,2] Kathryn R. Taylor,[1,2,3] Lynn Bjerke,[1,2] Matthew Clarke,[1,2] Mara Vinci,[1,2] Meera Nandhabalan,[1,2] Sara Temelso,[1,2] Sergey Popov,[1,2,4] Valeria Molinari,[1,2] Pichai Raman,[5,6] Angela J. Waanders,[5,7] Harry J. Han,[5,7] Saumya Gupta,[8] Lynley Marshall,[9] Stergios Zacharoulis,[9] Sucheta Vaidya,[9] Henry C. Mandeville,[10] Leslie R. Bridges,[11] Andrew J. Martin,[12]

(Author list continued on next page)

[1]Division of Molecular Pathology
[2]Division of Cancer Therapeutics
The Institute of Cancer Research, London, UK
[3]Department of Neurology, Stanford University School of Medicine, Stanford, CA, USA
[4]Department of Cellular Pathology, University Hospital of Wales, Cardiff, UK
[5]The Center for Data Driven Discovery in Biomedicine (D^{3}b)
[6]Division of Neurosurgery
[7]Division of Oncology
Children's Hospital of Philadelphia, Philadelphia, PA, USA
[8]Institute of Molecular Life Sciences, Swiss Institute of Bioinformatics, University of Zürich, Zürich, Switzerland
[9]Pediatric Oncology Drug Development Team, Children and Young People's Unit, Royal Marsden Hospital, Sutton, UK
[10]Department of Radiotherapy, Royal Marsden Hospital, Sutton, UK
[11]Department of Cellular Pathology
[12]Department of Neurosurgery
St George's Hospital NHS Trust, London, UK

(Affiliations continued on next page)

SUMMARY

We collated data from 157 unpublished cases of pediatric high-grade glioma and diffuse intrinsic pontine glioma and 20 publicly available datasets in an integrated analysis of >1,000 cases. We identified co-segregating mutations in histone-mutant subgroups including loss of *FBXW7* in H3.3G34R/V, *TOP3A* rearrangements in H3.3K27M, and *BCOR* mutations in H3.1K27M. Histone wild-type subgroups are refined by the presence of key oncogenic events or methylation profiles more closely resembling lower-grade tumors. Genomic aberrations increase with age, highlighting the infant population as biologically and clinically distinct. Uncommon pathway dysregulation is seen in small subsets of tumors, further defining the molecular diversity of the disease, opening up avenues for biological study and providing a basis for functionally defined future treatment stratification.

INTRODUCTION

Pediatric glioblastoma (pGBM) and diffuse intrinsic pontine glioma (DIPG) are high-grade glial tumors of children with a median overall survival of 9–15 months, a figure that has remained unmoved for decades (Jones et al., 2012). Although relatively rare in this age group (1.78 per 100,000 population), taken together, gliomas are nonetheless the most common malignant

Significance

High-grade and diffuse intrinsic pontine glioma in children are rare, incurable brain tumors with differing biology to adult cancers. An integrated genomic, epigenomic and transcriptomic analysis of >1,000 cases across all anatomical compartments of the CNS defines robust clinicopathological and molecular subgroups with distinct biological drivers. As modern classification schemes begin to recognize the diversity of this disease in the pediatric population, we provide a framework for meaningful further subcategorization and identify subgroup-restricted therapeutic targets.

 © 2017 The Author(s). Published by Elsevier Inc.
This is an open access article under the CC BY license (http://creativecommons.org/licenses/by/4.0/).

Safa Al-Sarraj,[13] Christopher Chandler,[14] Ho-Keung Ng,[15] Xingang Li,[16] Kun Mu,[17] Saoussen Trabelsi,[18] Dorra H'mida-Ben Brahim,[18] Alexei N. Kisljakov,[19] Dmitry M. Konovalov,[20] Andrew S. Moore,[21,22,23] Angel Montero Carcaboso,[24] Mariona Sunol,[24] Carmen de Torres,[24] Ofelia Cruz,[24] Jaume Mora,[24] Ludmila I. Shats,[25] João N. Stavale,[26] Lucas T. Bidinotto,[27] Rui M. Reis,[27,28] Natacha Entz-Werle,[29] Michael Farrell,[30] Jane Cryan,[30] Darach Crimmins,[31] John Caird,[31] Jane Pears,[32] Michelle Monje,[3] Marie-Anne Debily,[33] David Castel,[33] Jacques Grill,[33] Cynthia Hawkins,[34] Hamid Nikbakht,[35] Nada Jabado,[5] Suzanne J. Baker,[36] Stefan M. Pfister,[37,38,43] David T.W. Jones,[37,43] Maryam Fouladi,[39] André O. von Bueren,[40,41,42] Michael Baudis,[8] Adam Resnick,[5,6,7] and Chris Jones[1,2,44,*]

[13]Department of Neuropathology
[14]Department of Neurosurgery
Kings College Hospital, London, UK
[15]Department of Anatomical and Cellular Pathology, The Chinese University of Hong Kong, Hong Kong, China
[16]Department of Neurosurgery, Qilu Hospital of Shandong University and Brain Science Research Institute, Shandong University, Jinan, China
[17]Department of Pathology, Shandong University School of Medicine, Jinan, China
[18]Department of Cytogenetics and Reproductive Biology, Farhat Hached Hospital, Sousse, Tunisia
[19]Department of Pathology, Morozov Children's Hospital, Moscow, Russian Federation
[20]Department of Pathology, Dmitrii Rogachev Research and Clinical Centre of Pediatric Hematology, Oncology and Immunology, Moscow, Russian Federation
[21]UQ Child Health Research Centre, The University of Queensland, Brisbane, Australia
[22]Oncology Services Group, Children's Health Queensland Hospital and Health Service, Brisbane, Australia
[23]The University of Queensland Diamantina Institute, Translational Research Institute, Brisbane, Australia
[24]Institut de Recerca Sant Joan de Deu, Barcelona, Spain
[25]Division of Oncology, Pediatric Oncology and Radiotherapy, St Petersburg State Pediatric Medical University, St Petersburg, Russian Federation
[26]Department of Pathology, Federal University of São Paulo, São Paulo, São Paulo, Brazil
[27]Molecular Oncology Research Centre, Barretos Cancer Hospital, Barretos, São Paulo, Brazil
[28]Life and Health Sciences Research Institute (ICVS), Medical School, University of Minho, Braga, Portugal and ICVS/3B's-PT Government Associate Laboratory, Braga/Guimarães, Portugal
[29]Pédiatrie Onco-Hématologie - Pédiatrie III, Centre Hospitalier Régional et Universitaire Hautepierre, Strasbourg, France
[30]Histopathology Department, Beaumont Hospital, Dublin, Ireland
[31]Department of Neurosurgery, Temple Street Children's University Hospital, Dublin, Ireland
[32]Department of Paediatric Oncology, Our Lady's Children's Hospital, Dublin, Ireland
[33]Département de Cancerologie de l'Enfant et de l'Adolescent, Institut Gustav Roussy, Villejuif, France
[34]Pediatric Laboratory Medicine, Hospital for Sick Children, Toronto, Canada
[35]Department of Pediatrics, McGill University, Montreal, Canada
[36]Department of Developmental Neurobiology, St Jude Children's Research Hospital, Memphis, TN, USA
[37]Division of Pediatric Neuro-oncology, German Cancer Research Center (DKFZ), Heidelberg, Germany
[38]Department of Pediatric Hematology and Oncology, Heidelberg University Hospital, Heidelberg, Germany
[39]Department of Pediatrics, Cancer and Blood Diseases Institute, Cincinnati Children's Hospital, Cincinnati, OH, USA
[40]Department of Pediatrics, Division of Pediatric Hematology and Oncology, University Medical Center Goettingen, Goettingen, Germany
[41]Department of Pediatrics and Adolescent Medicine, Division of Pediatric Hematology and Oncology, University Hospital of Geneva, Geneva, Switzerland
[42]Department of Pediatrics, CANSEARCH Research Laboratory, Faculty of Medicine, University of Geneva, Geneva, Switzerland
[43]Hopp-Children's Cancer Center at the NCT Heidelberg (KiTZ), Heidelberg, Germany
[44]Lead Contact
*Correspondence: chris.jones@icr.ac.uk
http://dx.doi.org/10.1016/j.ccell.2017.08.017

brain tumors in children, and represent the greatest cause of cancer-related deaths under the age of 19 years (Ostrom et al., 2015). Unlike histologically similar lesions in adults, which tend to be restricted to the cerebral hemispheres, diffuse high-grade gliomas in childhood (pHGG) can occur throughout the CNS, with around half occurring in midline locations, in particular the thalamus and the pons (Jones and Baker, 2014), where the lack of available surgical options confers an especially poor prognosis (Kramm et al., 2011). Numerous clinical trials of chemotherapeutics and targeted agents extrapolated from adult GBM studies have failed to show a survival benefit, and more rationally derived approaches based upon an understanding of the childhood diseases are urgently needed (Jones et al., 2016).

It has become increasingly apparent that pHGG differ from their adult counterparts, with molecular profiling studies carried out over the last 6–7 years having incrementally identified key genetic and epigenetic differences in pHGG associated with distinct ages of onset, anatomical distribution, clinical outcome, and histopathological and radiological features (Jones and Baker, 2014; Sturm et al., 2014). In particular, the identification of unique recurrent mutations in genes encoding histones H3.3 and H3.1 (Schwartzentruber et al., 2012; Wu et al., 2012) have demonstrated the distinctiveness of the pediatric disease, with the G34R/V and K27M variants appearing to represent different clinicopathological and biological subgroups. This has been recognized by the World Health Organization (WHO) classification of CNS tumors, with the latest version including the novel entity, *diffuse midline glioma with H3K27 mutation* (Louis et al., 2016). Further refinements incorporating other clearly delineated subsets of the disease in future iterations appear likely and might prove clinically useful.

In addition to these uniquely defining histone mutations, detailed molecular profiling has served to identify numerous targets for therapeutic interventions. These include known oncogenes in adult glioma and other tumors with an elevated frequency in the childhood setting (e.g., *PDGFRA*) (Paugh et al., 2013; Puget et al., 2012) or certain rare histological variants (e.g., *BRAF* V600E) (Nicolaides et al., 2011; Schiffman et al., 2010), as well as others seemingly unique to DIPG (e.g., *ACVR1*) (Buczkowicz et al., 2014; Fontebasso et al., 2014; Taylor et al., 2014a; Wu et al., 2014). Future trials will need to exploit these targets, but also incorporate innovative designs that allow for selection of the patient populations within the wide spectrum of disease who are most likely to benefit from any novel agent (Jones et al., 2016).

Despite these advances, driven by the efforts of several international collaborative groups to collect and profile these rare tumors, individual publications remain necessarily modestly sized, involving a range of different platforms and analytical techniques. This leaves certain subgroups poorly represented across studies, widely differing individual gene frequencies in different cohorts, and an inability to draw robust conclusions across the whole spectrum of the disease. We have gathered together publicly available data, supplemented with 157 new cases, in order to provide a statistically robust, manually annotated resource cohort of >1,000 such tumors for interrogation.

RESULTS

Sample Cohort

In total, we obtained data from clinically annotated high-grade glioma (WHO, 2007, grade III or IV) or DIPG (radiologically diagnosed, grades II–IV) in 1,067 unique cases (Figure S1A). These were predominantly from children but also included young adults, in order to capture more *H3F3A* G34R/V mutations, as well as to explore an otherwise under-studied population. There was a median age at diagnosis of 9.8 years, and 982 cases aged 21 years or younger (Table S1). These included 910 taken from 20 published series (Barrow et al., 2011; Bax et al., 2010; Buczkowicz et al., 2014; Carvalho et al., 2014; Castel et al., 2015; Fontebasso et al., 2013, 2014; Grasso et al., 2015; International Cancer Genome Consortium PedBrain Tumor Project, 2016; Khuong-Quang et al., 2012; Korshunov et al., 2015; Paugh et al., 2010, 2011; Puget et al., 2012; Schwartzentruber et al., 2012; Sturm et al., 2012; Taylor et al., 2014a; Wu et al., 2012; Wu et al., 2014; Zarghooni et al., 2010) and 157 unpublished cases. The vast majority of samples were obtained pre-treatment (biopsy or resection, n = 913), as opposed to post-therapy (relapse or autopsy, n = 146). Samples were classified as occurring within the cerebral hemispheres (n = 482), brainstem (n = 323 in pons, of which 322 were DIPG; three additional cases were in the midbrain and one in the medulla) or other non-brainstem midline locations (n = 224, predominantly thalamus, but also cerebellum, spinal cord, ventricles, and others; referred to as "midline" for simplicity throughout) (Figure 1A). There was a significant association of anatomical location with age of diagnosis, with medians of 13.0 years for hemispheric, 10.0 years for midline, and 6.5 years for DIPG, respectively ($p < 0.0001$, ANOVA; all pairwise comparisons adjusted $p < 0.0001$, t test) (Figure 1B), in addition to clinical outcome, with a median overall survival of 18.0 months for hemispheric tumors (2 year overall survival 32%), 13.5 months for midline (2 year overall survival 21.4%), and 10.8 months for DIPG (2 year overall survival 5.2%; $p < 0.0001$ for all pairwise comparisons, log rank test) (Figure 1C). Children 3 years of age and younger had a markedly improved clinical outcome ($p = 0.0028$, log rank test), although this benefit was largely restricted to children 1 year and under (n = 40, 2 year survival 61%, $p < 0.0001$, log rank test), with this association significant in all anatomical locations ($p = 0.0402$, hemispheric; $p < 0.0001$, midline; $p = 0.00286$, pons, log rank test). There were, however, proportionally fewer midline and pontine tumors in <1-year-olds compared with 1- to 3-year-olds (12/40, 30.0% versus 46/85, 45.9%, $p = 0.0131$ Fisher's exact test) (Figures S1B and S1C).

Molecular Subgrouping

Hotspot mutation data for the genes encoding histone H3 were available or newly generated for 903 cases. At minimum, this included Sanger sequencing for *H3F3A* (H3.3) and *HIST1H3B* (H3.1); however, the absence of wider screening or next-generation sequencing data for 310 cases annotated as H3 wild-type (WT) means we cannot rule out rare variants in other H3.1 or H3.2 genes in those cases. In total, the cohort comprised 67 H3.3G34R/V (n = 63 G34R, n = 4 G34V), 316 H3.3K27M, 66 H3.1/3.2K27M (n = 62 *HIST1H3B*, n = 2 *HIST1H3C*, n = 2 *HIST2H3C*), and 454 WT. There were profound distinctions in anatomical location ($p < 0.0001$, Fisher's exact test) (Figure 1D), age at diagnosis ($p < 0.0001$, ANOVA; all pairwise comparisons adjusted $p < 0.0001$, t test) (Figure 1E), and overall survival ($p < 0.0001$, log rank test) (Figure 1F). H3.3G34R/V tumors were almost entirely restricted to the cerebral hemispheres (accounting for 16.2% total in this location, particularly parietal and temporal lobes), were found predominantly in adolescents and young adults (median 15.0 years), and had a longer overall survival compared with other H3 mutant groups (median 18.0 months, 2 year overall survival 27.3%, $p < 0.0001$ versus H3.3K27M, $p = 0.00209$ versus H3.1H27M, log rank test). H3.3K27M were spread throughout the midline and pons, where they account for 63.0% DIPG and 59.7% non-brainstem midline tumors. In all locations (including ten cases reported to present in the cortex), these mutations conferred a significantly shorter time to death from disease (overall median 11 months, 2 year overall survival 4.7%) (Figures S1D–S1F). H3.1/3.2K27M were highly specific to the pons (21.4% total) where they represent a younger age group (median 5.0 years) with a significantly longer overall survival (median 15.0 months) than H3.3K27M ($p = 0.00017$, log rank test) (Figure S1F). In multivariate analysis incorporating the histone mutations alongside age, WHO grade, and gender, K27M mutations in both H3.3 and H3.1 are independently associated with shorter survival ($p < 0.0001$, Cox proportional hazards model).

BRAF V600E status was available for 535 cases, with mutant cases (n = 32, 6.0%) present only in midline and hemispheric locations, and conferring a significantly improved prognosis (2 year survival 67%, $p < 0.0001$, log rank test) (Figures S1G–S1I). There was additional annotation for *IDH1* R132 mutation status in 640 cases (n = 40, 6.25%), representing a forebrain-restricted, significantly older group of patients (median 17.0

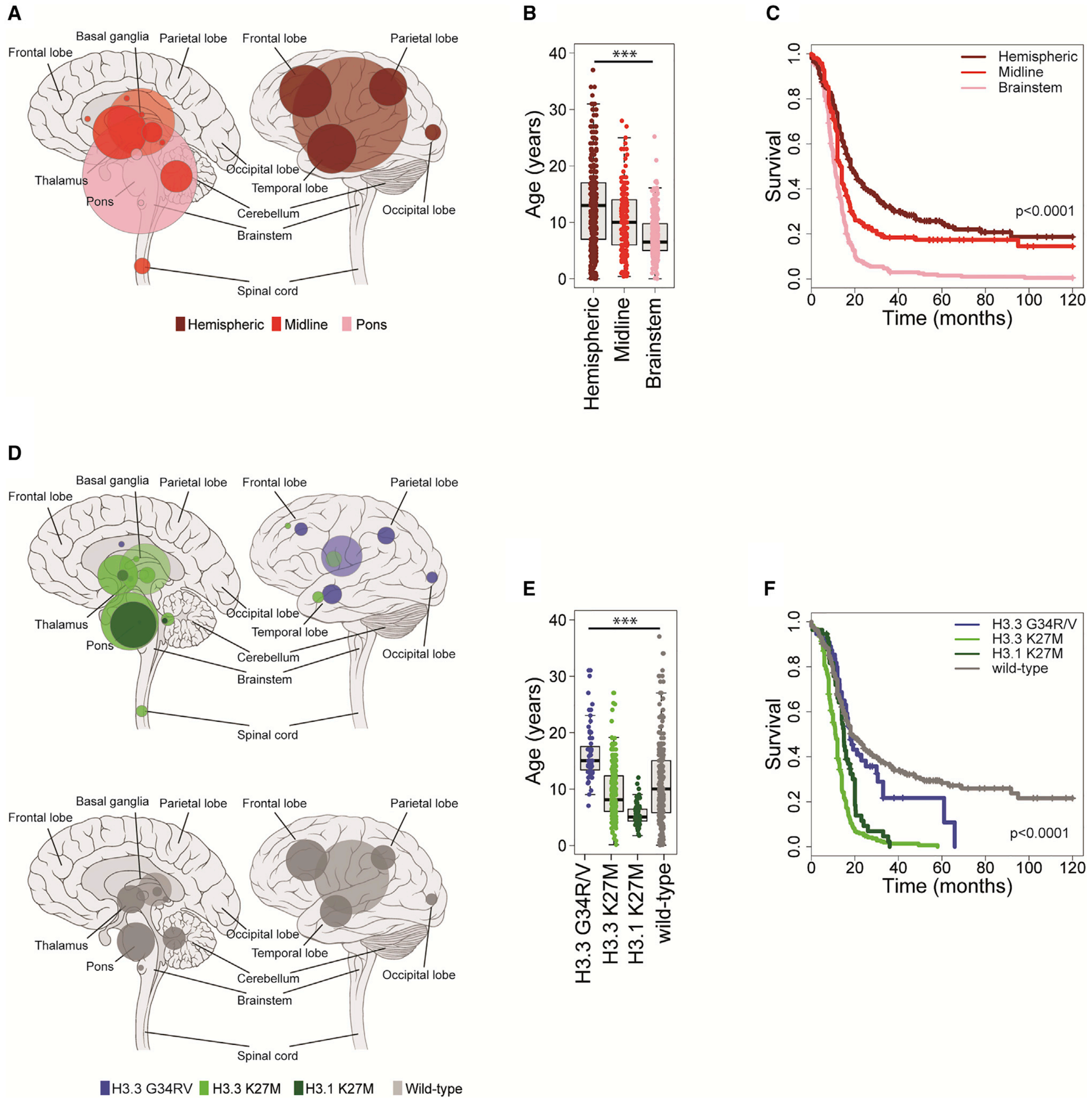

Figure 1. Clinicopathological and Molecular Subgroups of pHGG/DIPG

(A) Anatomical location of all high-grade glioma cases included in this study, taken from original publications (n = 1,033). Left, sagittal section showing internal structures; right, external view highlighting cerebral lobes. Hemispheric, dark red; non-brainstem midline structures, red; pons, pink. Radius of circle is proportional to the number of cases. Lighter shaded circles represent a non-specific designation of hemispheric, midline, or brainstem.

(B) Boxplot showing age at diagnosis of included cases, separated by anatomical location (n = 1,011). The thick line within the box is the median, the lower and upper limits of the boxes represent the first and third quartiles, and the whiskers 1.5× the interquartile range. ***Adjusted $p < 0.0001$ for all pairwise comparisons, t test.

(C) Kaplan-Meier plot of overall survival of cases separated by anatomical location, p value calculated by the log rank test (n = 811).

(D) Anatomical location of all cases separated by histone mutation (top, n = 441) and histone WT (bottom, n = 314). Left, sagittal section showing internal structures; right, external view highlighting cerebral lobes. Blue, H3.3G34R/V; green, H3.3K27M; dark green, H3.1K27M. Radius of circle is proportional to the number of cases. Lighter shaded circles represent a non-specific designation of hemispheric, midline, or brainstem.

(E) Boxplot showing age at diagnosis of included cases, separated by histone mutation (n = 753). The thick line within the box is the median, the lower and upper limits of the boxes represent the first and third quartiles, and the whiskers 1.5× the interquartile range. ***Adjusted $p < 0.0001$ for all pairwise comparisons, t test.

(F) Kaplan-Meier plot of overall survival of cases separated by histone mutation, p value calculated by the log rank test (n = 693). See also Figure S1 and Table S1.

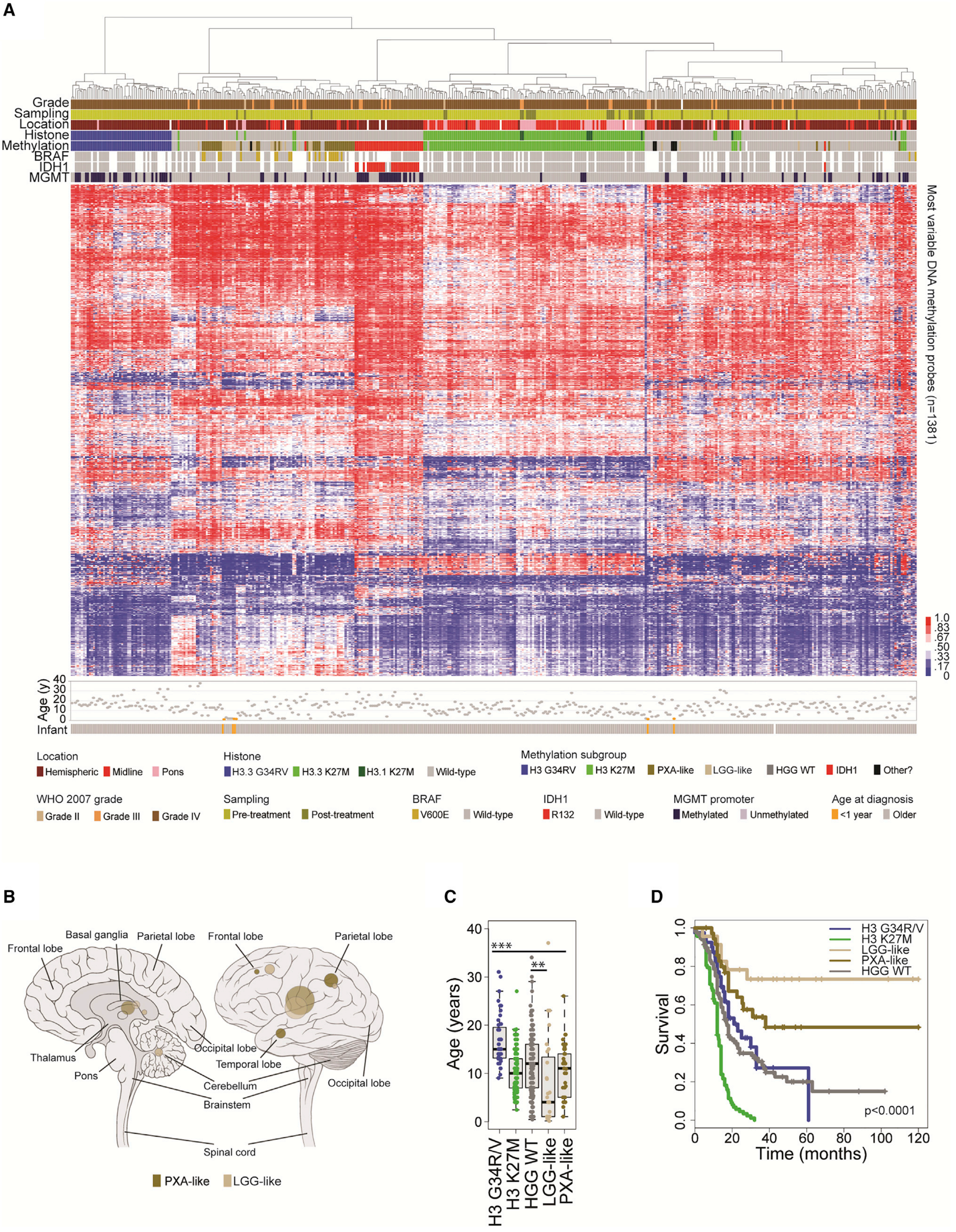

(legend on next page)

years, $p < 0.0001$, t test) with longer overall survival (2 year survival 59%, $p < 0.0001$, log rank test) (Figures S1J–S1L).

For 441 cases, Illumina 450k methylation BeadArray data was available, which provides robust classification into clinically meaningful epigenetic subgroups marked by recurrent genetic alterations (Korshunov et al., 2015, 2017). We used the Heidelberg brain tumor classifier to assign tumors into following subgroups: H3G34R/V (n = 51), H3K27M (n = 119), HGG WT (n = 156), IDH1 (n = 36), low-grade glioma (LGG)-like (n = 27), pleomorphic xanthoastrocytoma (PXA)-like (n = 43), and "other" (n = 9) (Figure S2A), visualized by hierarchical clustering (Figure 2A) (Table S2). As reported previously, these subgroups have profound differences in anatomical location (Figure 2B), age at diagnosis ($p < 0.00001$ ANOVA) (Figure 2C), and overall survival ($p < 0.0001$, log rank test) (Figure 2D), with LGG-like group representing a younger cohort (median 4.0 years, 10/16 infant cases under 1 year, $p < 0.0001$ Fisher's exact test) with excellent prognosis (2 year survival 74%, $p < 0.0001$ versus WT, log rank test), while the PXA-like group are enriched for *BRAF* V600E mutations (19/34, 56%) and carry an intermediate risk (median 38 months, 2 year survival 56%, p = 0.00423 versus WT, log rank test). After removing the PXA- and LGG-like groups, the remaining histone H3/IDH1 WT tumors had a 2 year survival of 23.5% (median overall survival 17.2 months). *MGMT* promoter methylation was significantly enriched in the H3G34R/V (65.1%, globally hypomethylated) and IDH1 (78.1%, globally hypermethylated) groups, and largely absent from H3K27M tumors (4.5%, all tests versus rest, $p < 0.0001$ Fisher's exact test) (Figure S2B). Total methylation was lowest in H3G34R/V (median beta value 0.452), and highest in the IDH subgroup (median beta value 0.520), as reported previously (Sturm et al., 2012); however it was also found to be significantly elevated in PXA-like tumors (median beta value 0.501, $p < 0.0001$ t test) (Figure S2C).

DNA Copy Number

High-quality DNA copy-number profiles were obtained from 834 unique cases of pHGG/DIPG, taken from BAC and oligonucleotide arrays (n = 112), SNP arrays (n = 128), 450k methylation arrays (n = 428), and whole-genome or exome sequencing (n = 325) (Table S3). Clustering on the basis of segmented $\log_2$ ratios highlighted some of the defining chromosomal features of the pediatric disease, including recurrent gains of chromosome 1q, and losses of chromosomes 13q and 14q (Figure 3A). There are also a significant proportion of tumors (n = 147, 17.6%) with few if any DNA copy-number changes, with no bias toward lower-resolution platforms (p = 0.134, Fisher's exact test), and the presence of other molecular markers obviating concerns of a substantial normal tissue contamination. These cases were found throughout the CNS, were younger at diagnosis (7.0 versus 10.3 years, $p < 0.0001$, t test) and had a longer overall survival (median 18.0 versus 14.0 months, p = 0.0107 log rank test) (Figure S3A). Common large-scale chromosomal alterations with prognostic significance included loss of 17p (n = 156), which targets *TP53* at 17p13.1 and confers a shorter overall survival in tumors of all locations and all subgroups (Figure S3B), and gains of 9q (n = 108), more broadly encompassing a region of structural rearrangement on 9q34 in medulloblastoma (Northcott et al., 2014), and correlating with shorter overall survival in multiple pHGG/DIPG subgroups (Figure S3C).

We used GISTIC (genomic identification of significant targets in cancer) in order to determine subgroup-specific copy-number drivers based on focality, amplitude, and recurrence of alterations. The most common focal events were the previously described high-level amplifications (Figure 3B) at 4q12 (*PDGFRA/KIT/KDR*, n = 77), 2p24.3 (*MYCN/ID2,* n = 42), chromosome 7 (7p11.2 (*EGFR,* n = 32), 7q21.2 (*CDK6,* n = 14), and 7q31.2 (*MET,* n = 19)) (Figures S3D–S3F), as well as focal deletions (Figure 3C) at 9p21.3 (*CDKN2A/CDKN2B,* n = 102) (Figure S3G). Amplifications conferred a shorter overall survival, and *CDKN2A/CDKN2B* deletion a better prognosis, either across the whole cohort or selected subgroups (Figures S3B–S3G). In addition, the aggregated data identified less-frequent alterations, recurrent across multiple studies, identifying pHGG/DIPG candidates including *NFIB* (nuclear factor I B, 9p23-p22.3, n = 4), *GAB2* (GRB2-associated binding protein 2, 11q14.1, n = 4), *SMARCE1* (SWI/SNF related, matrix associated, actin dependent regulator of chromatin, subfamily e, member 1, 17q21.2, n = 4), and others (Figures 3B and 3C).

Subgroup-Specific Alterations

When *IDH1*-mutant tumors were removed and the cohort restricted to those cases for which histone H3 status was available, we were able to investigate subgroup-specific DNA copy-number changes in 705 pHGG/DIPG (Figure 4A). Applying GISTIC within these case sets revealed specific focal events enriched within individual subgroups, including *AKT1* amplifications in H3.3G34R/V, *MYC* and *CCND2* amplification in H3.3K27M, and *MYCN/ID2*, *MDM4/PIK3C2B*, and *KRAS* amplification in H3 WT (Figure 4B) (Table S4). These latter events were generally restricted to hemispheric tumors, while *MYCN/ID2* were enriched in H3 WT DIPG (Figure S4A). H3.1K27M tumors generally lacked amplifications/deletions, but were instead characterized by frequent gains of 1q and the whole of chromosome

Figure 2. Methylation-based Subclassification of pHGG/DIPG

(A) Unsupervised hierarchical clustering and heatmap representation of β values for 441 samples profiled on the Illumina 450k BeadArray platform (red, high; blue, low). Samples are arranged in columns clustered by most variable 1,381 classifier probes. Age at diagnosis is provided below. Clinicopathological and molecular annotations are provided as bars according to the included key.

(B) Anatomical location of methylation-defined PXA-like (n = 43) and LGG-like (n = 27) cases. Left, sagittal section showing internal structures; right, external view highlighting cerebral lobes. Dark gold, PXA-like; tan, LGG-like. Radius of circle is proportional to the number of cases. Lighter shaded circles represent a non-specific designation of hemispheric, midline, or brainstem.

(C) Boxplot showing age at diagnosis of included cases, separated by simplified methylation subclass (n = 440). The thick line within the box is the median, the lower and upper limits of the boxes represent the first and third quartiles, and the whiskers 1.5× the interquartile range. ***Adjusted $p < 0.0001$ for all H3 G34R/V pairwise comparisons, t test; **adjusted $p < 0.01$ for LGG-like versus WT, t test.

(D) Kaplan-Meier plot of overall survival of cases separated by simplified methylation subclass, p value calculated by the log rank test (n = 307). See also Figure S2 and Table S2.

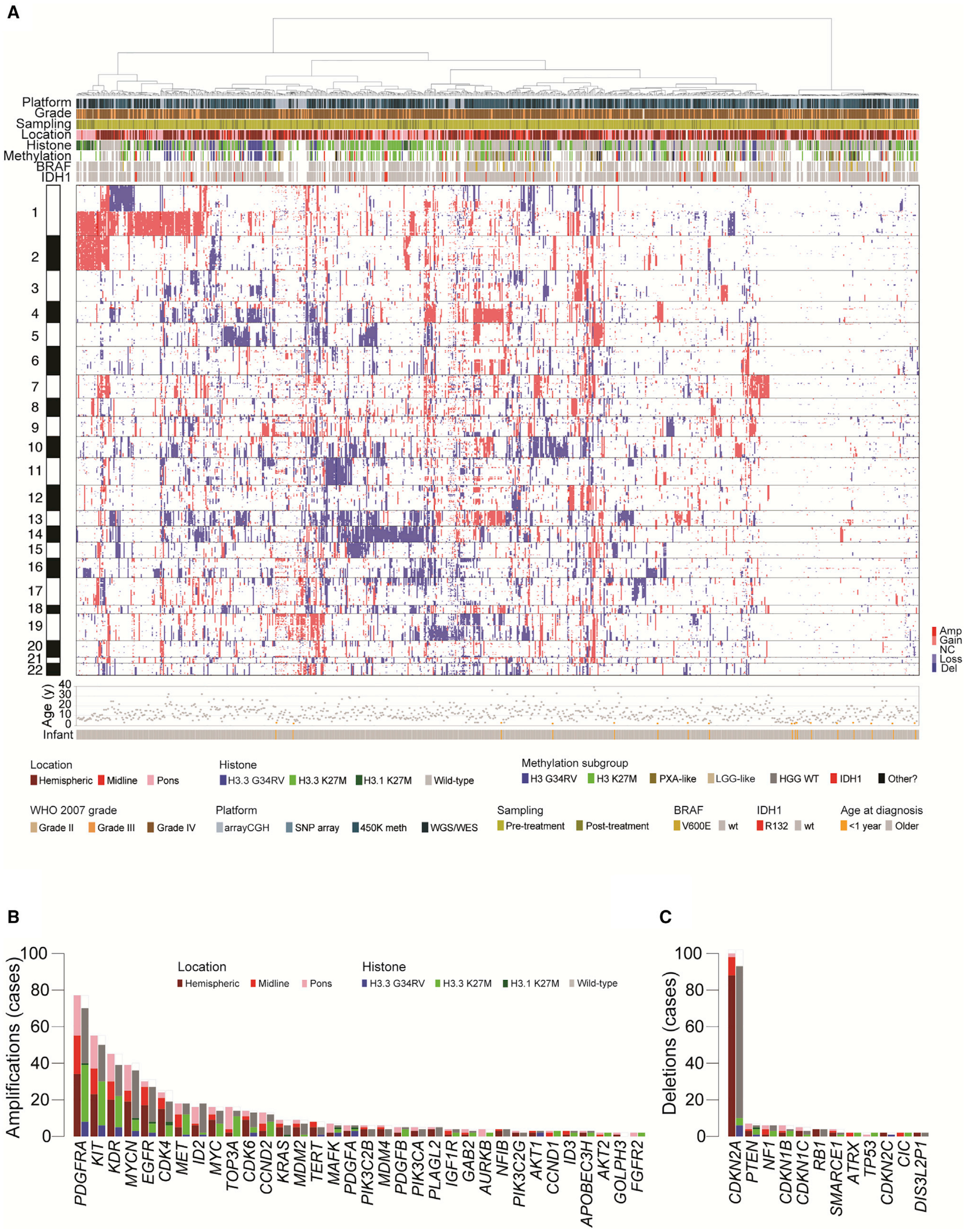

(legend on next page)

2, and the loss of 16q (Figure 4C). PXA-like tumors had frequent *CDKN2A/B* deletions and a unique loss at 1q, associated with shorter overall survival within this group (Figure S4B).

Whole-arm losses were also enriched in H3.3G34R/V tumors, specifically 3q, 4q, 5q, and 18q, where smallest regions of overlap were in some instances able to narrow the common region to a handful of candidate genes (Figure S4C). On chromosome 4q this appeared to target *FBXW7* at 4q31.3, also aligning with the GISTIC data (Figure 5A). Across three independent platforms, gene expression over the whole arm was significantly lower when 4q was lost (Agilent, p = 0.00231; Affymetrix, p = 0.000102; RNA sequencing (RNA-seq), p = 0.0398; Wilcoxon signed-rank test) (Figure S5A). (Table S5). There were also four patients with three different somatic coding mutations identified (below), two truncating and one missense, three of which were in hemispheric tumors, and two with *H3F3A* G34R (Figure 5B). In cases with 4q loss, median *FBXW7* gene expression was reduced compared with those with normal copy number (Agilent, p = 0.029; Affymetrix, p = 0.015; RNA-seq, p = 0.4; Mann-Whitney U test) (Figure 5C).

Within H3.3K27M tumors, we identified a recurrent amplification at 17p11.2 (n = 17; 170 kb to 11.96 Mb), across multiple platforms and significantly enriched in DIPGs, which appears to target *TOP3A* within these tumors (Figure 5D). Where available (n = 6) (Figure S5B), whole-genome sequencing data reveals this occurs via complex intra- and inter-chromosomal rearrangements (Figure 5E) leading to increased mRNA expression of *TOP3A* in amplified versus non-amplified cases in Agilent (n = 1), and Affymetrix and RNA-seq (p = 0.011 and p = 0.016, respectively, Mann-Whitney U test) data (Figure 5F) (Table S5). In an integrated dataset, *TOP3A* was the most differentially expressed gene in the region in amplified cases (adjusted p = 0.00856 Mann-Whitney U test). We further identified a single somatic missense mutation (C658Y) in an additional case of DIPG, and, taken together, *TOP3A* alterations were mutually exclusive with *ATRX* deletion/mutations found in H3.3K27M DIPG (0/13).

Whole-Genome and Exome Sequencing

Out of 372 sequenced cases (n = 118 whole genome, n = 247 exome, 7 both), we were able to retrieve raw data from 351 for integration of somatic variant calling (Table S6) and DNA copy-number changes. Of these, RNA-seq data was available for 47, allowing for candidate fusion gene nomination in 150 cases (RNA-seq or whole-genome sequencing restricted to high-confidence nominations in relevant pathway-associated genes, Table S6). Taking a conservative approach to variant calling given the disparate sequencing coverage (median 88×, range 16–295×), capture platforms, and availability of germline data, we report a median number of somatic single nucleotide variants (SNVs) and insertion/deletions (InDels) of 12, with 97% cases in the range 0–305. DNA copy neutral cases had significantly fewer somatic mutations (median 8.37 versus 21.32 SNVs/InDels per case), with those copy neutral cases also having no detectable mutations falling into the youngest age group (median = 3.9 years). There was only a modestly elevated mutation rate between samples taken post- compared with pre-treatment (1.5-fold, p = 0.056, t test) (Figure S6A). However, 11 cases had a vastly increased mutational burden, described as a hypermutator phenotype (median 13,735 SNVs/InDels, range 852–38,250), with distinctive mutational spectra from non-hypermutated pHGG/DIPG (Shlien et al., 2015), including three cases with plausible somatic activating *POLE* mutations (Figure S6B). *IDH1*-mutant cases were again excluded (n = 14), with genetic profiles identical to that described in adults for astrocytic tumors (13/14 *TP53*, 7/14 *ATRX* mutations), and oligodendroglial tumors (1p19q co-deletion, *TERT* promoter, *CIC*, *FUBP1* mutations) entirely absent (Figure S6C). We were thus left with an integrated dataset of 326 pHGG/DIPG, providing robust annotation of the most frequently altered genes across histone H3 subgroups and anatomical locations (Figure 6A). As well as known associations such as hemispheric H3.3G34R/V and *TP53/ATRX* (18/20, 90%; p = 0.0001), midline H3.3K27M and *FGFR1* (8/39, 20.5%; p = 0.212, not significant), pontine H3.1K27M and *ACVR1* (28/33, 84.8%; p < 0.0001), and PXA-like GBM with *BRAF* V600E (17/28, 60.7%; p < 0.0001), we also identified previously unrecognized co-segregating mutations including H3.3G34R/V and *ARID1B* (2/20, 10%; p = 0.0720), H3.3K27M DIPG and *ATM* and *ASXL1* (5/93, 10.7%; p = 0.0473), and H3.1K27M and *BCOR* (6/37, 16.2%; p = 0.0022, all Fisher's exact test) (Figure S6D). We also identified recurrent events in genes such as *PTPN23* (protein tyrosine phosphatase, non-receptor type 23, n = 5), *SOX10* (SRY-box 10, n = 5), *SRCAP* (Snf2-related CREBBP activator protein, n = 5), *DEPDC5* (DEP domain-contain 5, member of GATOR complex, n = 4), *SGK223* (PEAK1-related kinase activating pseudokinase, n = 4), and others (Figure 6B).

Integrated Pathway Analysis

Many of the rare variants we identified (Figure S6E) were found in genes associated with intracellular signaling pathways and processes more commonly targeted by high-frequency events, often in a mutually exclusive manner. In total, 297/326 (91.1%) of cases were found to harbor genetic alterations in one or more of nine key biological processes (Figure 7A). These included well-recognized pathways such as DNA repair (198/326, 60.7%), largely driven by *TP53* mutations (n = 160), but also by common mutually exclusive (p < 0.0001, Fisher's exact test) activating truncating alterations in *PPM1D* (n = 18), as well as heterozygous mutations in a diverse set of genes including those involved in homologous recombination (*ATM, BRCA2, BLM, ATR, PALB2, RAD50,* and *RAD51C*) and numerous Fanconi anemia genes (*BRIP1, FANCM, FANCA,* and *FANCG*), among others (Figure S7A). Although *TP53* is almost always found in concert with H3.3G34R/V in the cerebral hemispheres, these additional DNA repair pathway mutations were enriched in

Figure 3. DNA Copy-Number Aberrations in pHGG/DIPG

(A) Heatmap representation of segmented DNA copy number for 834 pHGG/DIPG profiled across one or more of seven different platforms (dark red, amplification; red, gain; dark blue, deletion; blue, loss). Samples are arranged in columns clustered by gene-level data across the whole genome. Age at diagnosis is provided below. Clinicopathological and molecular annotations are provided as bars according to the included key.

(B and C) Barplot of all recurrent focal amplifications (B) and deletions (C) across all 834 cases, in order of frequency, and colored independently by both anatomical location and histone mutation. See also Figure S3 and Table S3.

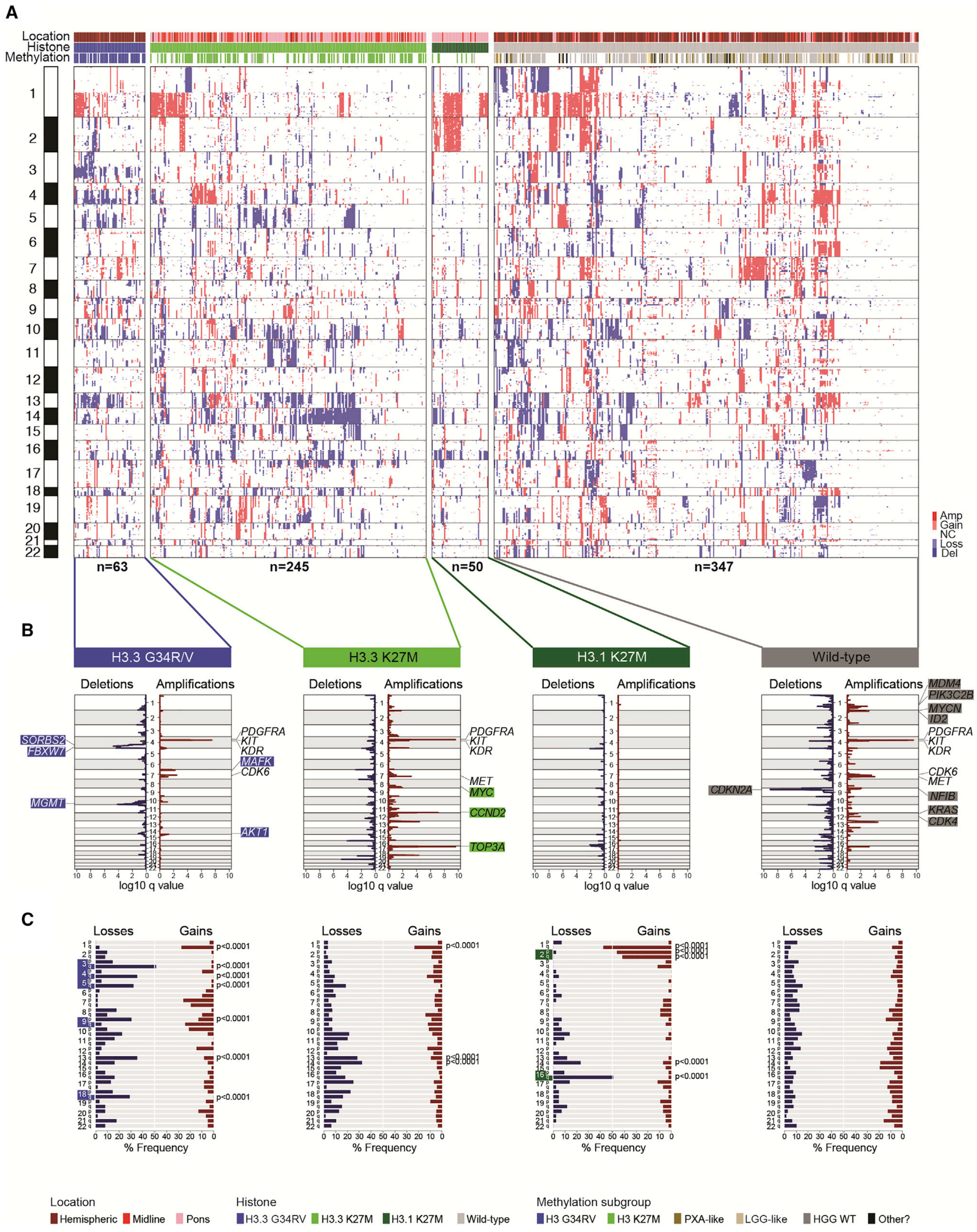

(legend on next page)

H3.3K27M DIPG (36/68, 52.9%). Also co-segregating with H3.3G34R/V and *TP53* is *ATRX*, although mutations/deletions of the latter gene are also frequently found in conjunction with H3.3K27M (28/54, 51.8%). *ATRX* accounts for a large proportion of the cases harboring mutations in genes coding for chromatin modifiers (54/118, 45.8%); however, there is a diverse set of readers, writers, and erasers also targeted at lower frequency, especially in DIPG, including the previously mentioned *BCOR* (n = 14) and *ASXL1* (n = 6) in addition to *SETD2* (n = 8), *KDM6B* (n = 6), *SETD1B* (n = 5), and *ARID1B* (n = 5) among many others (Figure S7B).

While *CDKN2A/CDKN2B* deletions were almost entirely absent from DIPG (1/154, 0.65%), dysregulation of the G_1/S cell-cycle checkpoint was common throughout anatomical locations and subgroups (82/326, 25.2%), with amplifications of *CCND2* and deletions of *CDKN2C* predominating in the pons (n = 5/7 and 5/5 DIPG, respectively), in contrast to recurrent homozygous *RB1* deletions and *CDK6* amplifications (n = 6/7 and 4/6 hemispheric) (Figure S4A) (Figure S7C).

Subgroup-specific dysregulation was also observed when considering discrete components of the RTK-PI3K-MAPK pathway. In total, 201/326 (61.7%) cases harbored alterations in any given node; however, for H3.3G34R/V this was predominantly at the RTK level (11/20, n = 9 *PDGFRA*) (Figure S7D), whereas H3.1K27M cases were enriched for PI3K/mTOR alterations (17/37, n = 9 *PIK3CA,* n = 5 *PIK3R1*) (Figure S7E), and H3 WT cases harbored the highest frequency of MAPK alterations (mainly *BRAF* V600E in PXA-like, n = 5/10 plus one *NF1*) (Figure S7F). *NRTK1-NRTK3* fusions were enriched in the infant group (4/6 fusions under 1 year old, median age 3.25 versus 8.5 years, p = 0.00033, t test) (Figure S7D). We further identified mutations in genes regulating mTOR signaling, including *TSC2* (n = 3), *RPTOR,* and *MTOR* itself (both n = 2), as well as a diverse series of SNVs and fusion candidates in MAPKs across all subgroups and locations (*MAP2K7*, *MAP3K15*, *MAP3K4* and others) (Figure S7F).

BMP signaling was significantly enriched in H3.1K27M DIPG due to the strong correlation with *ACVR1* mutations; however, alterations in other pathway members such as amplification of *ID2* (n = 10) or *ID3* (n = 3) and mutations in *BMP3* (n = 5), *BMP2K* (n = 3), and others across locations and subgroups, extends the proportion of tumors for which this pathway may be relevant (62/326, 18.7%) (Figure S7G). There was also a subset of cases harboring alterations in members of the WNT signaling pathway (16/326, 4.9%), including *AMER1, APC* (both n = 3), and *WNT8A*, *WNT9A, PLAGL2*, and *TCF7L2* (all n = 2) (Figure S7H).

Uniquely, the accumulated data uncovered a series of additional processes involved in maintenance of DNA replication, genome integrity, or transcriptional fidelity, targeted by infrequent but mutually exclusive alterations in pHGG and DIPG. These included mutations in splicing factors (*SF3A1, SF3A2, SF3A3, SF3B1, SF3B2,* and *SF3B3,* total n = 10), sister chromatid segregation (*STAG2, STAG3*, and *ESPL*1, total n = 9), pre-miRNA processing (*DICER* and *DROSHA*, total n = 4), DNA polymerases (*POLK, POLQ,* and *POLR1B,* total n = 4), as well as genes involved in centromere (*CENPB*, n = 3) and telomere maintenance (*PML*, n = 2; *TERT*, n = 7) (Figure S7I). *TERT* promoter mutations were found in 5/326 (1.5%) cases; however, alternative lengthening of telomeres (ALT) status was only available for 26 cases, although the 5 ALT-positive samples (19.2%) were mutually exclusive with *TERT* alterations.

We incorporated the integrated dataset into a pathway enrichment analysis (significant gene sets, false discovery rate [FDR] < 0.05, visualized as interaction networks by Cytoscape Enrichment Map) in order to gain additional insight into dysregulated biological processes. In addition to the subgroup-specific differential targeting of distinct nodes within common signaling pathways already described (e.g., RTK, PI3K/mTOR, and MAPK), additional dysregulated processes across the diversity of the disease were identified (Figure 7B). This revealed the perhaps not unexpected dysregulation of numerous developmental and CNS-associated gene sets (various immature organ systems, neuronal communication), but also previously unrecognized areas such as nuclear transport, cell migration, and the immune response (Table S7), which may provide further insight into disease biology as well as represent potential therapeutic strategies targeting key regulators of tumor phenotype. Indeed, neuronal communication with pGBM and DIPG cells is a recently demonstrated microenvironmental driver of pediatric glioma growth (Qin et al., 2017; Venkatesh et al., 2015).

Histone H3/IDH1 WT Subgroups

Finally, we wanted to explore those cases absent of any histone H3 or *IDH1* mutations in more depth. Using a t statistic-based stochastic neighbor embedding projection of the 450k methylation data, we identified three distinct clusters of tumors separate from the G34, K27, and IDH1 groups (Figure 8A). Consensus clustering of the H3/IDH1 WT cases alone confirmed the presence of three robust subgroups (Figure 8B), which were also recapitulated by unsupervised hierarchical clustering of the 10,000 probe classifier subset (Figure 8C). These groups included a largely hemispheric set of tumors containing, but not restricted to, the PXA- and LGG-like subgroups (WT-A). These tumors were driven by *BRAF* V600E, *NF1* mutations, or fusions in RTKs including *MET*, *FGFR2*, and *NTRK2,3* (Figure 8D). Although including many younger patients, the ages varied widely (Figure 8E). Regardless, this group had the best overall survival (median = 63 months, p < 0.0001 versus rest, log rank test) (Figure 8F), with the non-PXA/LGG-like tumors within this group themselves having an extended median survival time of

Figure 4. Subgroup-specific Copy-Number Changes in pHGG/DIPG

(A) Heatmap representation of segmented DNA copy number for 705 pHGG/DIPG separated for known histone mutation subgroup (dark red, amplification; red, gain; dark blue, deletion; blue, loss). Samples are arranged in columns clustered by gene-level data across the whole genome. Clinicopathological and molecular annotations are provided as bars according to the included key.

(B) GISTIC analysis of focal amplifications and deletions for histone mutation subgroups. Log_{10} values are plotted across the genome for both amplifications (dark red) and deletions (dark blue), with significantly enriched events labeled by likely driver genes. Subgroup-specific genes are highlighted by the appropriate color.

(C) Barplot of frequency of whole chromosomal arm gains (red) and losses (blue) for each subgroup. Significantly enriched alterations (p < 0.0001, Fisher's exact test) are labeled, with subgroup-specific arm changes highlighted by the appropriate color. See also Figure S4 and Table S4.

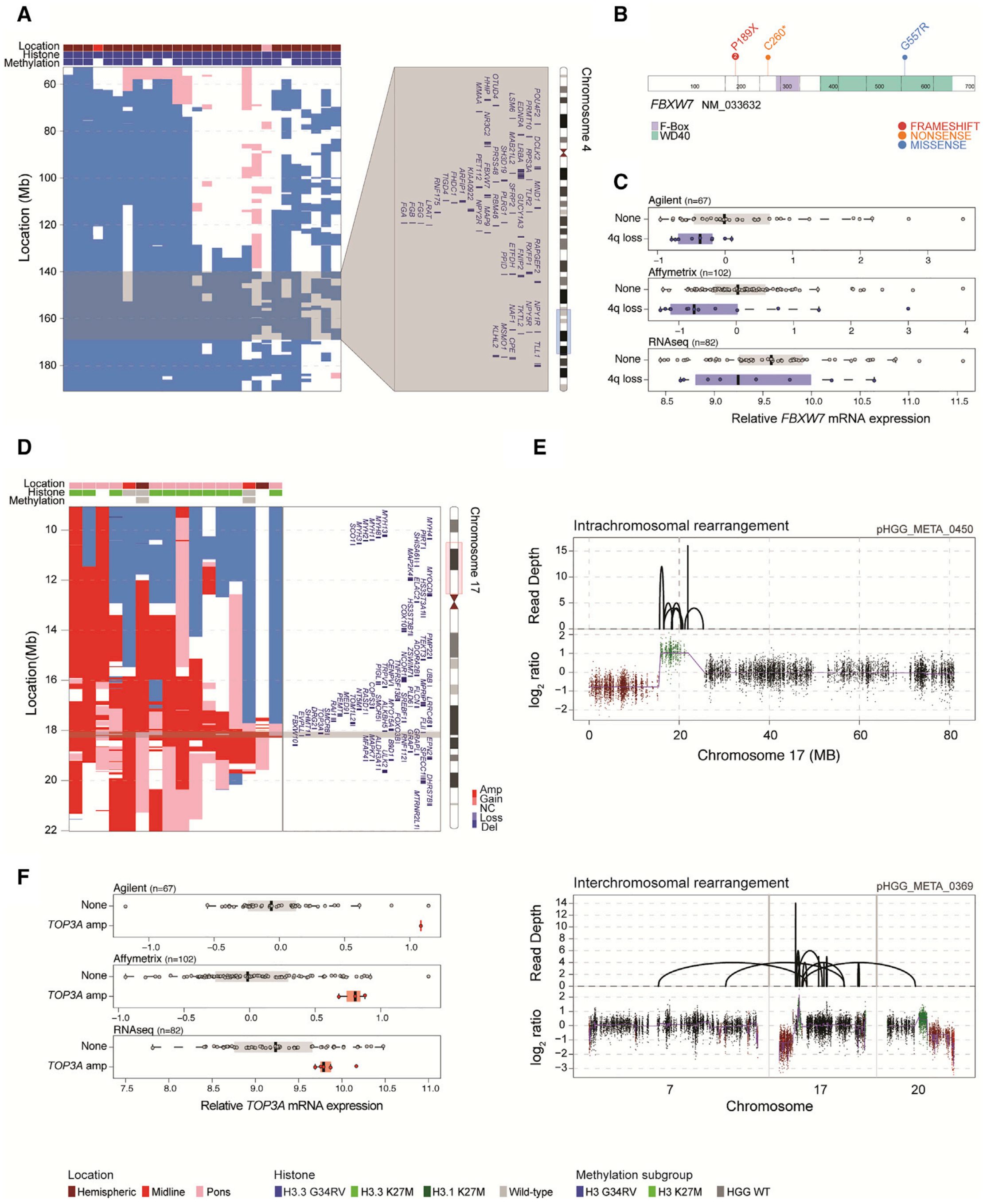

Figure 5. Alterations Targeting *FBXW7* in H3.3G34R/V pHGG and *TOP3A* in H3.3K27M DIPG

(A) Segmented exon-level DNA copy-number heatmaps for 4q loss in H3.3G34R/V tumors (dark red, amplification; red, gain; dark blue, deletion; blue, loss; n = 28). An ideogram of chromosome 4 is provided indicating enlarged genome browser view and genes within common regions targeted across samples (gray). Clinicopathological and molecular annotations are provided as bars according to the included key.

(B) Cartoon representation of amino acid position for four somatic mutations found in *FBXW7*, colored by annotated functional domains and numbers provided for recurrent variants.

(C) Boxplots representing gene expression differences between *FBXW7* lost/mutated cases (blue) and those with normal copy/WT (gray) in three independent gene expression platform datasets. The thick line within the box is the median, the lower and upper limits of the boxes represent the first and third quartiles, and the whiskers 1.5× the interquartile range.

(D) Segmented exon-level DNA copy-number heatmaps for 17p11.2 amplification in predominantly H3.3K27M DIPG (dark red, amplification; red, gain; dark blue, deletion; blue, loss; n = 17). Chromosome 17 ideogram is provided indicating enlarged genome browser view and genes within common regions targeted across samples (gray). Clinicopathological and molecular annotations are provided as bars according to the included key.

(legend continued on next page)

38 months ($p = 0.00928$ versus other H3/IDH1 WTs, log rank test). Taking an integrated gene expression profiling dataset (Figures S8A–S8E), these tumors were found to have upregulation of gene signatures associated with cytokine signaling and cell junction organization (Figures S8F and S8G). A second group of tumors (WT-B) were found in all anatomical compartments, and were distinguished by chromosome 2 gains (Figure 8C) and, most notably, by high-level amplifications in *EGFR*, *CDK6*, and *MYCN* ($p = 0.00033$, $p = 0.0299$, $p = 0.00037$, respectively, Fisher's exact test), with an imperfect overlay to the classifier "GBM_pedRTK" and "GBM_MYCN" groups (Figure 8D). This group had strong upregulation of MYC target genes, and had the poorest overall survival (median = 14 months) (Figure 8F). The remaining cases encompassed a methylation classifier group described as "HGG_MID," although in fact were split 80:20 hemispheric:midline (WT-C) (Figure 8C). This group was enriched for chromosome 1p and 20q loss, 17q gain ($p = 0.00595$, $p = 0.0286$, $p = 0.0478$, respectively, Fisher's exact test) (Figure 8C), harbored *PDGFRA* and *MET* amplifications ($p = 0.0159$, Fisher's exact test) (Figure 8D), and was strongly associated with the adult GBM-defined "Proneural" gene signature. These patients had a median survival of 18 months.

Although there remain tumors without detectable genetic alterations, we are nonetheless able to assign clinically meaningful subgroups with plausible driver alterations to the vast majority of pediatric HGG/DIPG.

DISCUSSION

Integrated molecular profiling has revolutionized the study of diffusely infiltrating high-grade glial tumors in children, providing evidence for unique mechanisms of molecular pathogenesis reflecting their distinct developmental origins (Baker et al., 2015; Jones and Baker, 2014). Although they are relatively rare, the present study accumulates 1,067 unique cases, a number similar to the aggregated analysis of the The Cancer Genome Atlas adult LGG/GBM cohorts (n = 1122, with grade III included in the "lower-grade" series) (Ceccarelli et al., 2016). Although there are clearly the usual caveats with such retrospective analyses of inconsistently annotated and treated cases, the cohort appears to represent a clinically useful approximation of the diversity of the pHGG/DIPG population.

In adults, the key distinction is between *IDH1* mutant (G-CIMP/*ATRX*/*TP53* or 1p19q co-deleted/*TERT* promoter mutated) and WT (classical, mesenchymal, PA-like) (Ceccarelli et al., 2016), whereas in the childhood setting *IDH1* mutations were restricted to a small proportion (6.25%) of tumors mostly in adolescents (representing the tail end of an overwhelmingly adult disease), and harbored only rare examples of the common alterations seen in WT adult GBM (e.g., 4.9% *EGFR* mutation/amplification). Instead, most prominent among the differences between pediatric and adult studies is the frequency of hotspot mutations in genes encoding histone H3 variants: 2/820 (0.2%) in adults (Ceccarelli et al., 2016) versus 449/893 (50.3%) in the present pHGG/DIPG series.

The importance of recurrent H3 mutations in the childhood setting has become increasingly clear since their unexpected discovery in 2012 (Schwartzentruber et al., 2012; Wu et al., 2012), with clear clinicopathological differences associated with distinct variants (Jones and Baker, 2014; Jones et al., 2016; Sturm et al., 2014), and fundamental insights into mechanisms of epigenetically linked tumorigenesis (Bender et al., 2013; Bjerke et al., 2013; Chan et al., 2013; Funato et al., 2014). Despite this, precisely how we can target these mutations clinically remains elusive (Grasso et al., 2015; Hennika et al., 2017). Data from such a large series of tumors demonstrates the robustness of the histone-defined subgroups in terms of anatomical location, age of incidence, clinical outcome, methylation and gene expression profiles, copy-number changes, co-segregating somatic mutations, and pathway dysregulation. As most of the non-histone molecular alterations previously reported in pHGG/DIPG have been relatively infrequent, it is only through this accumulated dataset that we have been able to uncover subgroup-specific genes/processes that may play a role as diagnostic, prognostic, or predictive markers or drug targets in these diseases.

H3.3G34R/V-mutant tumors are restricted to the cerebral hemispheres and co-segregate with *ATRX* and *TP53* mutations; they are also the only pediatric subgroup to harbor frequent *MGMT* promoter methylation (Korshunov et al., 2015). Copy-number profiling of 63 cases highlighted a significant enrichment of chromosomal arm losses at 3q, 4q, 5q, and 18q, further refined by smallest region of overlap and GISTIC analyses. At 4q31.3, this identified *FBXW7* as a candidate gene target of the loss. *FBXW7* encodes a member of the F box protein family and is frequently deleted/mutated in cancer, supporting its tumor-suppressive function (Davis et al., 2014); notably in relation to H3.3G34R/V it has been reported to play a role in MYC/MYCN stabilization through its action as a component of the SCF-like ubiquitin ligase complex that targets MYC/MYCN for proteasomal degradation (Welcker et al., 2004; Yada et al., 2004). With MYCN upregulated in H3.3G34R/V tumors through differential H3K36me3 binding (Bjerke et al., 2013), this observation adds to the mechanisms by which Myc proteins exert their influence in this subgroup, and provide further rationale for the observed effects of disrupting these interactions, such as with Aurora kinase A inhibitors which target the direct interaction between the catalytic domain of Aurora A and a site flanking Myc Box I that also binds SCF/FbxW7 (Richards et al., 2016).

H3.3K27M tumors are found in two-thirds of DIPG and non-brainstem midline pHGG alike, where they are associated with a shorter overall survival in both locations, as well as in the small number of cases reported in the cortex. Although presumably reflecting a common or overlapping origin, the pattern of co-segregating mutations differ, e.g., *PDGFRA* alterations predominating

(E) Sequencing coverage (top) and $\log_2$ ratio plot (bottom) for chromosomes 7, 17, and 20 for two cases, showing complex intra- or inter-chromosomal rearrangements leading to specific copy-number amplification of *TOP3A*.

(F) Boxplots representing gene expression differences between *TOP3A* amplified cases (red) and those with normal copy (gray) in three independent gene expression platform datasets. The thick line within the box is the median, the lower and upper limits of the boxes represent the first and third quartiles, and the whiskers 1.5× the interquartile range. See also Figure S5 and Table S5.

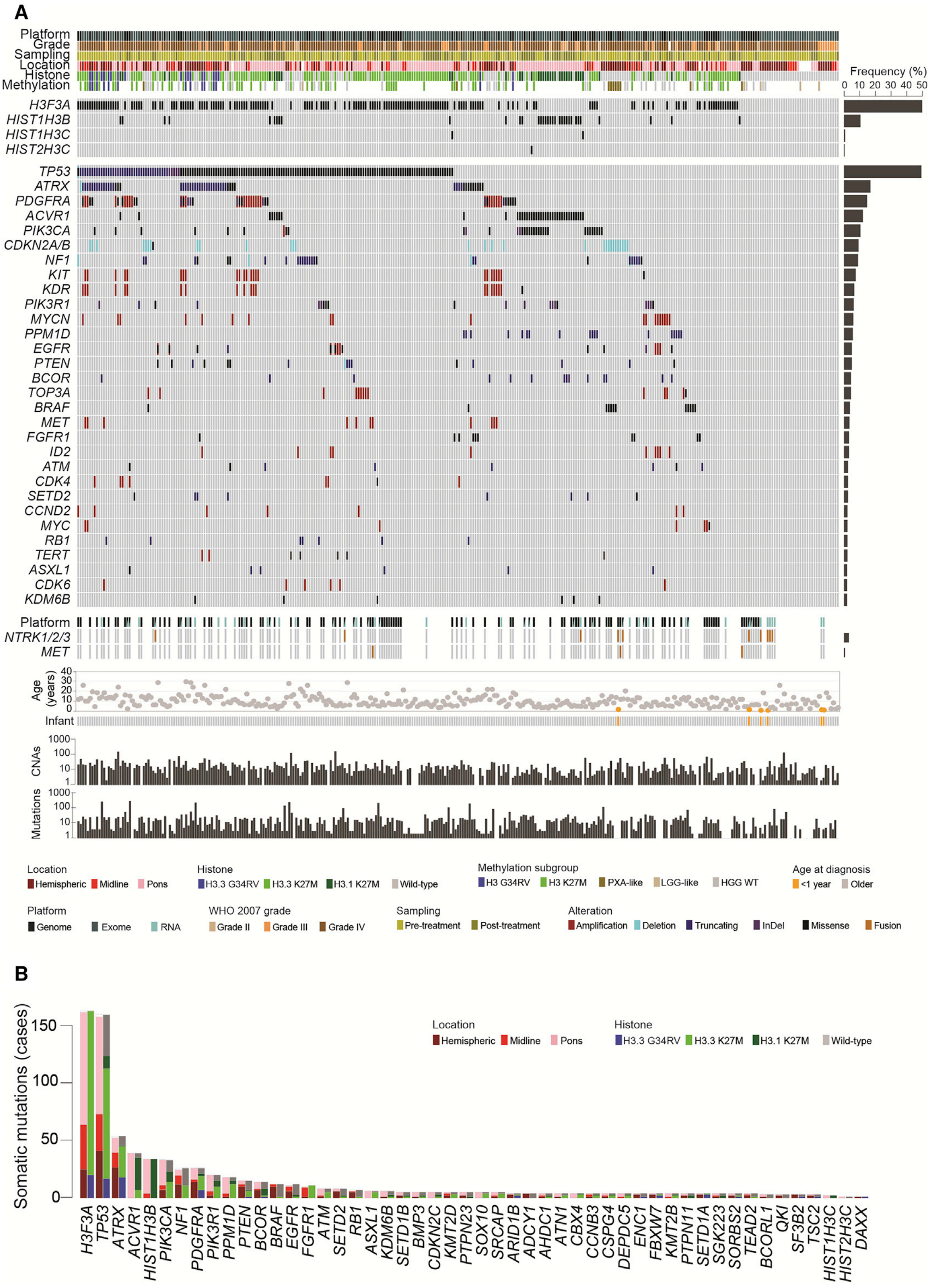

Figure 6. Somatic Mutations in pHGG/DIPG

(A) Oncoprint representation of an integrated annotation of somatic mutations and DNA copy-number changes for the 30 most frequently altered genes in 326 pHGG/DIPG (n $\geq$ 6, frequency barplot on the right). Selected common fusion events are also shown where available. Samples are arranged in columns with genes labeled along rows. Age at diagnosis is provided below. Underneath, barplots are provided on a $\log_{10}$ scale for numbers of copy-number aberrations and somatic mutations per case. Clinicopathological and molecular annotations are provided as bars according to the included key.

(B) Barplot of all recurrent somatic mutations across all 326 cases, in order of frequency, and colored independently by both anatomical location and histone mutation. See also Figure S6 and Table S6.

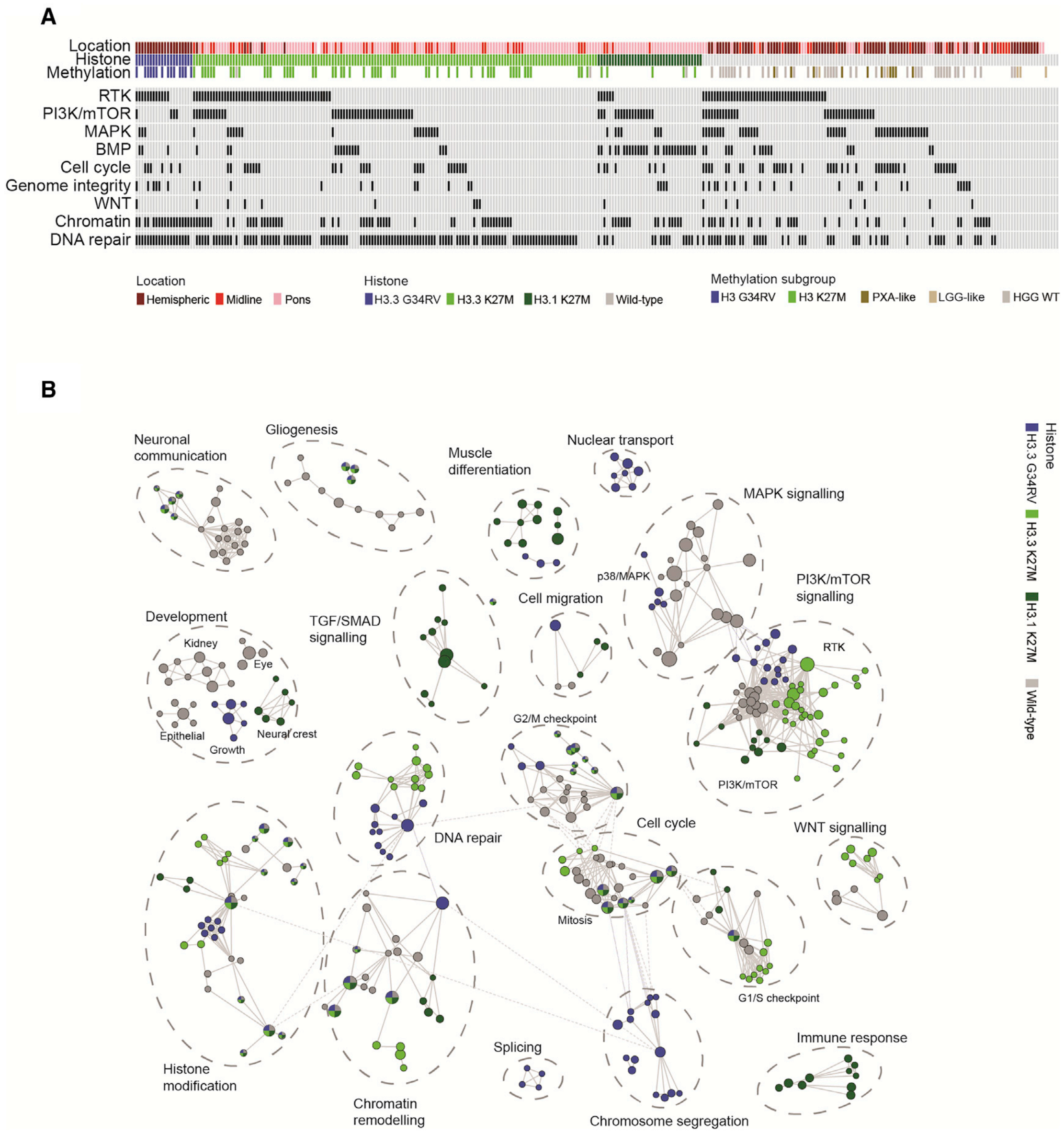

Figure 7. Integrated Pathway Analysis of pHGG/DIPG

(A) Oncoprint-style representation of an integrated annotation of somatic mutations and DNA copy-number changes in one or more of nine commonly targeted pathways in 326 pHGG/DIPG ($n \geq 6$, frequency barplot on the right). Samples are arranged in columns with pathways labeled along rows. Clinicopathological and molecular annotations are provided as bars according to the included key.

(B) Pathway enrichment analysis of pHGG/DIPG subgroups. Distinct pathways and biological processes between the subgroups are colored appropriately (FDR $q < 0.01$). Nodes represent enriched gene sets, which are grouped and annotated by their similarity according to related gene sets. Node size is proportional to the total number of genes within each gene set. The illustrated network map was simplified by manual curation to remove general and uninformative sub-networks. See also Figure S7 and Table S7.

in the pons, and *FGFR1* variants being largely restricted to the thalamus (Fontebasso et al., 2014). Our analysis of more than 300 cases further identifies differential amplification of *CCND2* (DIPG) and *CDK4* (non-brainstem midline), and, most strikingly, an amplification at 17p11.2 involving *TOP3A* in H3.3K27M DIPG. This complex rearrangement often involves loss of the more distal part of 17p involving *TP53*, along with intra- or inter-chromosomal translocations to deliver an increase in *TOP3A* copy number and gene expression. *TOP3A* encodes DNA topoisomerase III alpha, which forms a complex with BLM (Wu et al., 2000), has an important role in homologous recombination (Yang et al., 2010), and has been implicated in maintenance of the ALT phenotype (Temime-Smaali et al., 2009). Notably, *TOP3A* amplification/mutation was found to be mutually exclusive with *ATRX* mutation in H3.3K27M DIPG, with depletion by small interfering RNA reducing ALT cell survival (Temime-Smaali et al., 2008), and therefore represents a potential therapeutic target in this subgroup.

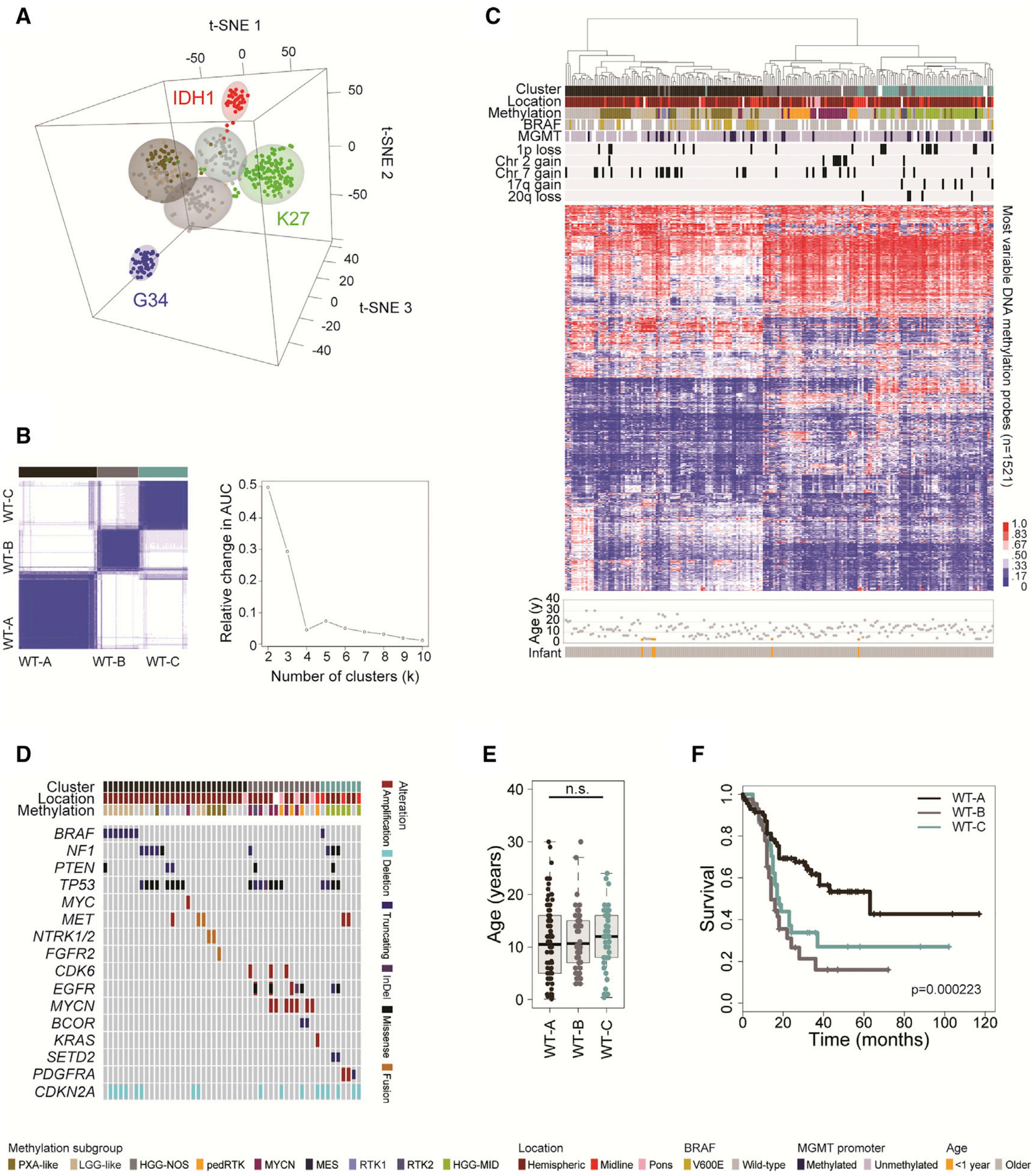

Figure 8. Integrated Analysis of H3/IDH1 WT pHGG/DIPG

(A) t Statistic-based stochastic neighbor embedding (t-SNE) projection of the combined 450k methylation dataset (n = 441). The first three projections are plotted in the x, y, and z axes, with samples represented by dots colored by histone H3G34 (blue), H3K27 (green), IDH1 (red), PXA-like (dark gold), LGG-like (tan), and "others" (gray).

(B) K means consensus clustering on the H3/IDH1 WT cases highlights three stable clusters (left, black/brown [WT-A], gray/pink [WT-B], and dark cyan [WT-C]) as the most robust subdivision of the data (right, area under the curve analysis for different cluster numbers).

(C) Unsupervised hierarchical clustering and attendant heatmap of the H3/IDH1 WT cases (n = 219). Samples are arranged in columns clustered by the most variable 1,521 classifier probes. Age at diagnosis is provided below. Clinicopathological and molecular annotations are provided as bars according to the included key.

(D) Oncoprint representation of an integrated annotation of somatic mutations and DNA copy-number changes for the H3/IDH1 WT cases (n = 50). Samples are arranged in columns with genes labeled along rows. Age at diagnosis is provided below. Clinicopathological and molecular annotations are provided as bars according to the included key.

(E) Boxplot showing age at diagnosis of H3/IDH1 WT subgroups, separated by anatomical location (n = 190). The thick line within the box is the median, the lower and upper limits of the boxes represent the first and third quartiles, and the whiskers 1.5× the interquartile range.

(F) Kaplan-Meier plot of overall survival of H3/IDH1 WT subgroups separated by anatomical location, p value calculated by the log rank test (n = 150). See also Figure S8 and Table S8.

H3.1K27M tumors by contrast are restricted to the pons, patients are younger and with a slightly longer survival (Castel et al., 2015), and are largely defined at the copy-number level by whole chromosomal arm gains and losses (Taylor et al., 2014a). They have the well-recognized association with *ACVR1* mutation (Taylor et al., 2014b); however, we also identify an enrichment of downstream PI3K pathway mutations (*PIK3CA* and *PIK3R1*) in comparison with the largely upstream RTK alterations present in H3.3K27M DIPGs, important in designing stratified trials and combinatorial therapies. Further association with mutations of the BCL6 repressor gene *BCOR*, commonly altered in medulloblastomas, neuroepithelial tumors, and sarcomas, highlights a further avenue for interventional study through its regulation of the SHH pathway (Tiberi et al., 2014).

In H3/IDH1 WT cases, methylation profiling refines the heterogeneous collection of tumors, particularly identifying two predominantly hemispheric intermediate risk subgroups that classify alongside other entities (PXA- and LGG-like) in a larger series of better outcome tumors (WT-A). These had already been strongly linked with dysregulation of the MAPK pathway (*BRAF* V600E) (Korshunov et al., 2015) along with *CDKN2A*/*CDKN2B* deletion (Nicolaides et al., 2011). However, with molecular markers such as losses at 1q and 17p appearing to confer a worse outcome there may be more than one subgroup within this entity, and a co-clustering group of H3/IDH1 WT tumors appeared distinctly driven by somatic *NF1* mutation. The LGG-like tumors generally occur in very young patients, where the appearance of few genetic alterations and a significantly better prognosis is shared by the majority of infant HGG. Gene fusion events, including those targeting *NTRKs1-NTRK3*, are common in this age range. Notably this enhanced survival is restricted to patients diagnosed under 12 months of age, and is not recapitulated in the 1–3 year age group, although this is the common clinical definition of "infants" in many centers.

Excluding these morphologically high-grade but biologically and clinically low-grade tumors, the remaining H3/IDH1 WT cases can be further split into two poor-outcome groups driven by *EGFR*/*MYCN*/*CDK6* (WT-B) or *PDGFRA*/*MET* (WT-C) or amplifications. These groups overlap with other methylation-based classification groups (PDGFRA versus EGFR versus MYCN (Korshunov et al., 2017); "GBM_pedRTK" versus "GBM_MYCN" versus "HGG_MID" (molecularneuropathology.org/mnp), however, are uniquely defined here spanning anatomical locations and integrated with sequencing data. Further exploration of these heterogeneous subgroups in order to refine integrated molecular diagnostics to prioritize patient subpopulations for stratified treatment remains a priority.

The remarkable biological diversity spanning pediatric malignant glioma is finally demonstrated by the <5% tumors with a hypermutator phenotype, some of the greatest mutational burdens in all human cancer, and candidates for immune checkpoint inhibitors (Bouffet et al., 2016). Previously unrecognized processes altered in small subsets of tumors identified through this meta-analysis, such as the splicing machinery, miRNA regulation, and the WNT pathway offer further areas for exploration. The thorough cataloging of dysregulated molecular pathways across the whole spectrum of pediatric diffusely infiltrating gliomas in the present study provides the basis for novel therapeutic development.

STAR★METHODS

Detailed methods are provided in the online version of this paper and include the following:

- KEY RESOURCES TABLE
- CONTACT FOR REAGENT AND RESOURCE SHARING
- EXPERIMENTAL MODEL AND SUBJECT DETAILS
  - Patient Samples
- METHOD DETAILS
  - Nucleic Acid Extraction
  - Sanger Sequencing of *H3F3A* / *HIST1H3B*
  - Methylation Profiling
  - Exome and RNA Sequencing
- QUANTIFICATION AND STATISTICAL ANALYSIS
  - Published Data Sources
  - DNA Copy Number
  - DNA Methylation
  - mRNA Expression
  - Sequence Analysis
  - Candidate Fusion Gene Nomination
  - Inferred Tumor Purity
  - Integrated Analysis of Driver Events
  - Pathway Analysis
  - Statistical Analysis
- DATA AND SOFTWARE AVAILABILITY
- ADDITIONAL RESOURCES

SUPPLEMENTAL INFORMATION

Supplemental Information includes eight figures and eight tables and can be found with this article online at http://dx.doi.org/10.1016/j.ccell.2017.08.017.

AUTHOR CONTRIBUTIONS

A.M., M.F., A.O.v.B., M.B., and C.J. conceived the study. A.M. and C.J. analyzed data and wrote the manuscript. A.B., D.C., E.I.D., J.F.S., K.T., L.B., M.V., M.N., S.T., and V.M. performed molecular analysis of unpublished samples. M.C., S.P., L.R.B., S.A.-S., A.N.K., D.M.K., K.M., K.-K.N., M.S., and C.K. carried out histopathological assessment of cases. M.M., J.G., C.H., N.J., S.J.B., S.M.P., and D.T.W.J. provided data. L.M., S.Z., S.V., H.C.M., A.J.M., C.C., N.E.-W., J.P., J.S., R.M.R., A.S.M., L.S., S.T., D.H.-B.B., A.M.C., C.d.T., O.C., J.M., and M.M. provided samples and clinical annotation. M.B., P.R., A.J.W., H.J.H., S.G., and A.R. constructed analytical and visualization tools and databases. All authors approved the manuscript.

ACKNOWLEDGMENTS

This work was supported by Cancer Research UK (grants C13468/A13982 and C13468/A23536), CRIS Cancer Foundation, Abbie's Army and the DIPG Collaborative, the Cure Starts Now Foundation, Christopher's Smile, McKenna Claire Foundation, Lyla Nsouli Foundation, National Institutes of Health (grants R01NS085336 and R01NS091620), The Dragon Master Foundation, The Kortney Rose Foundation, The Musella Foundation For Brain Tumor Research & Information, Gray Matters Foundation, Pediatric Brain Tumor Foundation, and the INSTINCT network funded by The Brain Tumour Charity, Great Ormond Street Children's Charity, and Children with Cancer UK. The authors acknowledge NHS funding to the NIHR Biomedical Research Centre at The Royal Marsden and the ICR. LTB was recipient of São Paulo Research Foundation fellowships (2011/08523-7 and 2012/08287-4). This study makes use of data generated by the St. Jude Children's Research Hospital – Washington University Pediatric Cancer Genome Project, the Hospital for Sick Children, the McGill University-DKFZ Pediatric Brain Tumor Consortium, the International Cancer Genomics Consortium PedBrain Project and the Cancer Research

UK Genomics Initiative (C13468/A14078) and supports the Children's Brain Tumor Tissue Consortium and The Pacific Pediatric Neuro-Oncology Consortium (PNOC) CAVATICA: Project OPEN DIPG initiative.

Received: May 5, 2017
Revised: July 14, 2017
Accepted: August 29, 2017
Published: September 28, 2017

REFERENCES

Baker, S.J., Ellison, D.W., and Gutmann, D.H. (2015). Pediatric gliomas as neurodevelopmental disorders. Glia *64*, 879–895.

Barrow, J., Adamowicz-Brice, M., Cartmill, M., MacArthur, D., Lowe, J., Robson, K., Brundler, M.A., Walker, D.A., Coyle, B., and Grundy, R. (2011). Homozygous loss of ADAM3A revealed by genome-wide analysis of pediatric high-grade glioma and diffuse intrinsic pontine gliomas. Neuro Oncol. *13*, 212–222.

Bax, D.A., Mackay, A., Little, S.E., Carvalho, D., Viana-Pereira, M., Tamber, N., Grigoriadis, A.E., Ashworth, A., Reis, R.M., Ellison, D.W., et al. (2010). A distinct spectrum of copy number aberrations in pediatric high-grade gliomas. Clin. Cancer Res. *16*, 3368–3377.

Bender, S., Tang, Y., Lindroth, A.M., Hovestadt, V., Jones, D.T., Kool, M., Zapatka, M., Northcott, P.A., Sturm, D., Wang, W., et al. (2013). Reduced H3K27me3 and DNA hypomethylation are major drivers of gene expression in K27M mutant pediatric high-grade gliomas. Cancer Cell *24*, 660–672.

Bjerke, L., Mackay, A., Nandhabalan, M., Burford, A., Jury, A., Popov, S., Bax, D.A., Carvalho, D., Taylor, K.R., Vinci, M., et al. (2013). Histone H3.3. mutations drive pediatric glioblastoma through upregulation of MYCN. Cancer Discov. *3*, 512–519.

Bouffet, E., Larouche, V., Campbell, B.B., Merico, D., de Borja, R., Aronson, M., Durno, C., Krueger, J., Cabric, V., Ramaswamy, V., et al. (2016). Immune checkpoint inhibition for hypermutant glioblastoma multiforme resulting from germline biallelic mismatch repair deficiency. J. Clin. Oncol. *34*, 2206.

Buczkowicz, P., Hoeman, C., Rakopoulos, P., Pajovic, S., Letourneau, L., Dzamba, M., Morrison, A., Lewis, P., Bouffet, E., Bartels, U., et al. (2014). Genomic analysis of diffuse intrinsic pontine gliomas identifies three molecular subgroups and recurrent activating ACVR1 mutations. Nat. Genet. *46*, 451–456.

Cai, H., Kumar, N., and Baudis, M. (2012). arrayMap: a reference resource for genomic copy number imbalances in human malignancies. PLoS One *7*, e36944.

Cai, H., Kumar, N., Ai, N., Gupta, S., Rath, P., and Baudis, M. (2014). Progenetix: 12 years of oncogenomic data curation. Nucleic Acids Res. *42*, D1055–D1062.

Carvalho, D., Mackay, A., Bjerke, L., Grundy, R.G., Lopes, C., Reis, R.M., and Jones, C. (2014). The prognostic role of intragenic copy number breakpoints and identification of novel fusion genes in paediatric high grade glioma. Acta Neuropathol. Commun. *2*, 23.

Castel, D., Philippe, C., Calmon, R., Le Dret, L., Truffaux, N., Boddaert, N., Pages, M., Taylor, K.R., Saulnier, P., Lacroix, L., et al. (2015). Histone H3F3A and HIST1H3B K27M mutations define two subgroups of diffuse intrinsic pontine gliomas with different prognosis and phenotypes. Acta Neuropathol. *130*, 815–827.

Ceccarelli, M., Barthel, F.P., Malta, T.M., Sabedot, T.S., Salama, S.R., Murray, B.A., Morozova, O., Newton, Y., Radenbaugh, A., Pagnotta, S.M., et al. (2016). Molecular profiling reveals biologically discrete subsets and pathways of progression in diffuse glioma. Cell *164*, 550–563.

Chan, K.M., Fang, D., Gan, H., Hashizume, R., Yu, C., Schroeder, M., Gupta, N., Mueller, S., James, C.D., Jenkins, R., et al. (2013). The histone H3.3K27M mutation in pediatric glioma reprograms H3K27 methylation and gene expression. Genes Dev. *27*, 985–990.

Davis, R.J., Welcker, M., and Clurman, B.E. (2014). Tumor suppression by the Fbw7 ubiquitin ligase: mechanisms and opportunities. Cancer Cell *26*, 455–464.

Fontebasso, A.M., Schwartzentruber, J., Khuong-Quang, D.A., Liu, X.Y., Sturm, D., Korshunov, A., Jones, D.T., Witt, H., Kool, M., Albrecht, S., et al. (2013). Mutations in SETD2 and genes affecting histone H3K36 methylation target hemispheric high-grade gliomas. Acta Neuropathol. *125*, 659–669.

Fontebasso, A.M., Papillon-Cavanagh, S., Schwartzentruber, J., Nikbakht, H., Gerges, N., Fiset, P.O., Bechet, D., Faury, D., De Jay, N., Ramkissoon, L.A., et al. (2014). Recurrent somatic mutations in ACVR1 in pediatric midline high-grade astrocytoma. Nat. Genet. *46*, 462–466.

Funato, K., Major, T., Lewis, P.W., Allis, C.D., and Tabar, V. (2014). Use of human embryonic stem cells to model pediatric gliomas with H3.3K27M histone mutation. Science *346*, 1529–1533.

Grasso, C.S., Tang, Y., Truffaux, N., Berlow, N.E., Liu, L., Debily, M.A., Quist, M.J., Davis, L.E., Huang, E.C., Woo, P.J., et al. (2015). Functionally defined therapeutic targets in diffuse intrinsic pontine glioma. Nat. Med. *21*, 555–559.

Hennika, T., Hu, G., Olaciregui, N.G., Barton, K.L., Ehteda, A., Chitranjan, A., Chang, C., Gifford, A.J., Tsoli, M., Ziegler, D.S., et al. (2017). Pre-clinical study of panobinostat in xenograft and genetically engineered murine diffuse intrinsic pontine glioma models. PLoS One *12*, e0169485.

International Cancer Genome Consortium PedBrain Tumor Project (2016). Recurrent MET fusion genes represent a drug target in pediatric glioblastoma. Nat. Med. *22*, 1314–1320.

Jones, C., and Baker, S.J. (2014). Unique genetic and epigenetic mechanisms driving paediatric diffuse high-grade glioma. Nat. Rev. Cancer *14*, 651–661.

Jones, C., Perryman, L., and Hargrave, D. (2012). Paediatric and adult malignant glioma: close relatives or distant cousins? Nat. Rev. Clin. Oncol. *9*, 400–413.

Jones, C., Karajannis, M.A., Jones, D.T., Kieran, M.W., Monje, M., Baker, S.J., Becher, O.J., Cho, Y.J., Gupta, N., Hawkins, C., et al. (2016). Pediatric high-grade glioma: biologically and clinically in need of new thinking. Neuro Oncol. *19*, 153.

Khuong-Quang, D.A., Buczkowicz, P., Rakopoulos, P., Liu, X.Y., Fontebasso, A.M., Bouffet, E., Bartels, U., Albrecht, S., Schwartzentruber, J., Letourneau, L., et al. (2012). K27M mutation in histone H3.3 defines clinically and biologically distinct subgroups of pediatric diffuse intrinsic pontine gliomas. Acta Neuropathol. *124*, 439–447.

Korshunov, A., Ryzhova, M., Hovestadt, V., Bender, S., Sturm, D., Capper, D., Meyer, J., Schrimpf, D., Kool, M., Northcott, P.A., et al. (2015). Integrated analysis of pediatric glioblastoma reveals a subset of biologically favorable tumors with associated molecular prognostic markers. Acta Neuropathol. *129*, 669–678.

Korshunov, A., Schrimpf, D., Ryzhova, M., Sturm, D., Chavez, L., Hovestadt, V., Sharma, T., Habel, A., Burford, A., Jones, C., et al. (2017). H3-/IDH-wild type pediatric glioblastoma is comprised of molecularly and prognostically distinct subtypes with associated oncogenic drivers. Acta Neuropathol. http://dx.doi.org/10.1007/s00401-017-1710-1.

Kramm, C.M., Butenhoff, S., Rausche, U., Warmuth-Metz, M., Kortmann, R.D., Pietsch, T., Gnekow, A., Jorch, N., Janssen, G., Berthold, F., et al. (2011). Thalamic high-grade gliomas in children: a distinct clinical subset? Neuro Oncol. *13*, 680–689.

Louis, D.N., Perry, A., Reifenberger, G., von Deimling, A., Figarella-Branger, D., Cavenee, W.K., Ohgaki, H., Wiestler, O.D., Kleihues, P., and Ellison, D.W. (2016). The 2016 World Health Organization classification of tumors of the central nervous system: a summary. Acta Neuropathol. *131*, 803–820.

Nicolaides, T.P., Li, H., Solomon, D.A., Hariono, S., Hashizume, R., Barkovich, K., Baker, S.J., Paugh, B.S., Jones, C., Forshew, T., et al. (2011). Targeted therapy for BRAFV600E malignant astrocytoma. Clin. Cancer Res. *17*, 7595–7604.

Northcott, P.A., Lee, C., Zichner, T., Stutz, A.M., Erkek, S., Kawauchi, D., Shih, D.J., Hovestadt, V., Zapatka, M., Sturm, D., et al. (2014). Enhancer hijacking activates GFI1 family oncogenes in medulloblastoma. Nature *511*, 428–434.

Ostrom, Q.T., Gittleman, H., Fulop, J., Liu, M., Blanda, R., Kromer, C., Wolinsky, Y., Kruchko, C., and Barnholtz-Sloan, J.S. (2015). CBTRUS statistical report: primary brain and central nervous system tumors diagnosed in the United States in 2008–2012. Neuro Oncol. *17* (*Suppl 4*), iv1–iv62.

Paugh, B.S., Qu, C., Jones, C., Liu, Z., Adamowicz-Brice, M., Zhang, J., Bax, D.A., Coyle, B., Barrow, J., Hargrave, D., et al. (2010). Integrated molecular genetic profiling of pediatric high-grade gliomas reveals key differences with the adult disease. J. Clin. Oncol. *28*, 3061–3068.

Paugh, B.S., Broniscer, A., Qu, C., Miller, C.P., Zhang, J., Tatevossian, R.G., Olson, J.M., Geyer, J.R., Chi, S.N., da Silva, N.S., et al. (2011). Genome-wide analyses identify recurrent amplifications of receptor tyrosine kinases and cell-cycle regulatory genes in diffuse intrinsic pontine glioma. J. Clin. Oncol. *29*, 3999–4006.

Paugh, B.S., Zhu, X., Qu, C., Endersby, R., Diaz, A.K., Zhang, J., Bax, D.A., Carvalho, D., Reis, R.M., Onar-Thomas, A., et al. (2013). Novel oncogenic PDGFRA mutations in pediatric high-grade gliomas. Cancer Res. *73*, 6219–6229.

Puget, S., Philippe, C., Bax, D.A., Job, B., Varlet, P., Junier, M.P., Andreiuolo, F., Carvalho, D., Reis, R., Guerrini-Rousseau, L., et al. (2012). Mesenchymal transition and PDGFRA amplification/mutation are key distinct oncogenic events in pediatric diffuse intrinsic pontine gliomas. PLoS One *7*, e30313.

Qin, E.Y., Cooper, D.D., Abbott, K.L., Lennon, J., Nagaraja, S., Mackay, A., Jones, C., Vogel, H., Jackson, P.K., and Monje, M. (2017). Neural Precursor-Derived Pleiotrophin Mediates Subventricular Zone Invasion by Glioma. Cell *170* (5), 845–859.

Richards, M.W., Burgess, S.G., Poon, E., Carstensen, A., Eilers, M., Chesler, L., and Bayliss, R. (2016). Structural basis of N-Myc binding by Aurora-A and its destabilization by kinase inhibitors. Proc. Natl. Acad. Sci. USA *113*, 13726–13731.

Schiffman, J.D., Hodgson, J.G., VandenBerg, S.R., Flaherty, P., Polley, M.Y., Yu, M., Fisher, P.G., Rowitch, D.H., Ford, J.M., Berger, M.S., et al. (2010). Oncogenic BRAF mutation with CDKN2A inactivation is characteristic of a subset of pediatric malignant astrocytomas. Cancer Res. *70*, 512–519.

Schwartzentruber, J., Korshunov, A., Liu, X.Y., Jones, D.T., Pfaff, E., Jacob, K., Sturm, D., Fontebasso, A.M., Quang, D.A., Tonjes, M., et al. (2012). Driver mutations in histone H3.3 and chromatin remodelling genes in paediatric glioblastoma. Nature *482*, 226–231.

Shlien, A., Campbell, B.B., de Borja, R., Alexandrov, L.B., Merico, D., Wedge, D., Van Loo, P., Tarpey, P.S., Coupland, P., Behjati, S., et al. (2015). Combined hereditary and somatic mutations of replication error repair genes result in rapid onset of ultra-hypermutated cancers. Nat. Genet. *47*, 257–262.

Sturm, D., Witt, H., Hovestadt, V., Khuong-Quang, D.A., Jones, D.T., Konermann, C., Pfaff, E., Tonjes, M., Sill, M., Bender, S., et al. (2012). Hotspot mutations in H3F3A and IDH1 define distinct epigenetic and biological subgroups of glioblastoma. Cancer Cell *22*, 425–437.

Sturm, D., Bender, S., Jones, D.T., Lichter, P., Grill, J., Becher, O., Hawkins, C., Majewski, J., Jones, C., Costello, J.F., et al. (2014). Paediatric and adult glioblastoma: multiform (epi)genomic culprits emerge. Nat. Rev. Cancer *14*, 92–107.

Taylor, K.R., Mackay, A., Truffaux, N., Butterfield, Y.S., Morozova, O., Philippe, C., Castel, D., Grasso, C.S., Vinci, M., Carvalho, D., et al. (2014a). Recurrent activating ACVR1 mutations in diffuse intrinsic pontine glioma. Nat. Genet. *46*, 457–461.

Taylor, K.R., Vinci, M., Bullock, A.N., and Jones, C. (2014b). ACVR1 mutations in DIPG: lessons learned from FOP. Cancer Res. *74*, 4565–4570.

Temime-Smaali, N., Guittat, L., Wenner, T., Bayart, E., Douarre, C., Gomez, D., Giraud-Panis, M.J., Londono-Vallejo, A., Gilson, E., Amor-Gueret, M., et al. (2008). Topoisomerase IIIalpha is required for normal proliferation and telomere stability in alternative lengthening of telomeres. EMBO J. *27*, 1513–1524.

Temime-Smaali, N., Guittat, L., Sidibe, A., Shin-ya, K., Trentesaux, C., and Riou, J.F. (2009). The G-quadruplex ligand telomestatin impairs binding of topoisomerase IIIalpha to G-quadruplex-forming oligonucleotides and uncaps telomeres in ALT cells. PLoS One *4*, e6919.

Tiberi, L., Bonnefont, J., van den Ameele, J., Le Bon, S.D., Herpoel, A., Bilheu, A., Baron, B.W., and Vanderhaeghen, P. (2014). A BCL6/BCOR/SIRT1 complex triggers neurogenesis and suppresses medulloblastoma by repressing Sonic Hedgehog signaling. Cancer Cell *26*, 797–812.

Venkatesh, H.S., Johung, T.B., Caretti, V., Noll, A., Tang, Y., Nagaraja, S., Gibson, E.M., Mount, C.W., Polepalli, J., Mitra, S.S., et al. (2015). Neuronal activity promotes glioma growth through Neuroligin-3 secretion. Cell *161*, 803–816.

Welcker, M., Orian, A., Jin, J., Grim, J.E., Harper, J.W., Eisenman, R.N., and Clurman, B.E. (2004). The Fbw7 tumor suppressor regulates glycogen synthase kinase 3 phosphorylation-dependent c-Myc protein degradation. Proc. Natl. Acad. Sci. USA *101*, 9085–9090.

Wu, L., Davies, S.L., North, P.S., Goulaouic, H., Riou, J.F., Turley, H., Gatter, K.C., and Hickson, I.D. (2000). The Bloom's syndrome gene product interacts with topoisomerase III. J. Biol. Chem. *275*, 9636–9644.

Wu, G., Broniscer, A., McEachron, T.A., Lu, C., Paugh, B.S., Becksfort, J., Qu, C., Ding, L., Huether, R., Parker, M., et al. (2012). Somatic histone H3 alterations in pediatric diffuse intrinsic pontine gliomas and non-brainstem glioblastomas. Nat. Genet. *44*, 251–253.

Wu, G., Diaz, A.K., Paugh, B.S., Rankin, S.L., Ju, B., Li, Y., Zhu, X., Qu, C., Chen, X., Zhang, J., et al. (2014). The genomic landscape of diffuse intrinsic pontine glioma and pediatric non-brainstem high-grade glioma. Nat. Genet. *46*, 444–450.

Yada, M., Hatakeyama, S., Kamura, T., Nishiyama, M., Tsunematsu, R., Imaki, H., Ishida, N., Okumura, F., Nakayama, K., and Nakayama, K.I. (2004). Phosphorylation-dependent degradation of c-Myc is mediated by the F-box protein Fbw7. EMBO J. *23*, 2116–2125.

Yang, J., Bachrati, C.Z., Ou, J., Hickson, I.D., and Brown, G.W. (2010). Human topoisomerase IIIalpha is a single-stranded DNA decatenase that is stimulated by BLM and RMI1. J. Biol. Chem. *285*, 21426–21436.

Zarghooni, M., Bartels, U., Lee, E., Buczkowicz, P., Morrison, A., Huang, A., Bouffet, E., and Hawkins, C. (2010). Whole-genome profiling of pediatric diffuse intrinsic pontine gliomas highlights platelet-derived growth factor receptor alpha and poly (ADP-ribose) polymerase as potential therapeutic targets. J. Clin. Oncol. *28*, 1337–1344.

STAR★METHODS

KEY RESOURCES TABLE

REAGENT or RESOURCE	SOURCE	IDENTIFIER
Critical Commercial Assays		
DNeasy blood & tissue kit	Qiagen	69504
QIAmp DNA FFPE tissue kit	Qiagen	56404
RNeasy mini kit	Qiagen	74104
QIAquick PCR purification kit	Qiagen	28104
BigDye terminator v3.1 mix	Thermo Fisher	4337455
SureSelect Human All Exon capture set V4<br>SureSelect Human All Exon capture set V5	Agilent	5190-4666<br>5190-6208
SureSelect RNA Capture, 0.5-2.9Mb	Agilent	5190-4944
Deposited Data		
Exome and RNA sequencing of new samples	This paper	EGA: EGAS00001002314
Illumina methylation BeadChip profiling of new samples	This paper	ArrayExpress: E-MTAB-5528
Sequencing and methylation data	This paper	cavatica.org
Oligonucleotides		
Primer: H3F3A_forward TGGCTCGTACAAAGCAGACT	This paper	N/A
Primer: H3F3A_reverse ATATGGATACATACAAGAGAGACT	This paper	N/A
Primer: HIST1H3B_forward GGGCAGGAGCCTCTCTTAAT	This paper	N/A
Primer: HIST1H3B _ reverse ACCAAGTAGGCCTCACAAGC	This paper	N/A
Software and Algorithms		
Mutation Surveyor	SoftGenetics	softgenetics.com/mutationSurveyor.php
4Peaks	Nucleobytes	http://nucleobytes.com/4peaks/
limma	BioConductor	bioconductor.org/packages/release/bioc/html/limma.html
marray	BioConductor	bioconductor.org/packages/release/bioc/html/marray.html
aroma.affymetrix	The Comprehensive R Archive Network	cran.rstudio.com/web/packages/aroma.affymetrix/index.html
aroma.cn	The Comprehensive R Archive Network	cran.r-project.org/web/packages/aroma.cn/index.html
minfi	BioConductor	bioconductor.org/packages/release/bioc/html/minfi.html
conumee	BioConductor	bioconductor.org/packages/release/bioc/html/conumee.html
BEDtools	University of Utah	github.com/arq5x/bedtools2
DNAcopy	BioConductor	bioconductor.org/packages/release/bioc/html/DNAcopy.html
gviz	BioConductor	bioconductor.org/packages/release/bioc/html/Gviz.html
GISTIC	Broad Institute	oftware.broadinstitute.org/software/cprg/?q=node/31
CopyNumber450kData	BioConductor	bioconductor.org/packages/release/data/experiment/html/CopyNumber450kData.html
MNP	DKFZ Heidelberg	molecularneuropathology.org/mnp

(*Continued on next page*)

Continued		
REAGENT or RESOURCE	SOURCE	IDENTIFIER
tSNE	The Comprehensive R Archive Network	cran.r-project.org/web/packages/Rtsne/index.html
rgl	The Comprehensive R Archive Network	cran.r-project.org/web/packages/rgl/index.html
affy	BioConductor	bioconductor.org/packages/release/bioc/html/affy.html
Bowtie2	Johns Hopkins University	bowtie-bio.sourceforge.net/bowtie2/index.shtml
TopHat	Johns Hopkins University	ccb.jhu.edu/software/tophat/index.shtml
cufflinks	University of Washington	ole-trapnell-lab.github.io/cufflinks/cufflinks/
DESeq2	BioConductor	bioconductor.org/packages/release/bioc/html/DESeq2.html
Gene Set Enrichment Analysis	Broad Institute	http://software.broadinstitute.org/gsea
bwa	Sanger Institute	http://bio-bwa.sourceforge.net/
Genome Analysis Toolkit	Broad Institute	oftware.broadinstitute.org/gatk/
Variant Effect predictor	Ensembl tools	ensembl.org/info/docs/variation/vep
ANNOVAR	Children's Hospital of Philadelphia	annovar.openbioinformatics.org/en/latest/
ExAc	Broad Institute	exac.broadinstitute.org/
BCBio	Harvard TH Chan	bcb.io/
SIFT	J Craig Venter Institute	sift.jcvi.org
PolyPhen	Harvard	genetics.bwh.harvard.edu/pph2
ChimeraScan	University of Michigan	omictools.com/chimerascan-tool
Breakdancer	Washington University of St Louis	breakdancer.sourceforge.net
ASCAT	Francis Crick Institute	rick.ac.uk/peter-van-loo/software/ASCAT
Oncoprinter	Memorial Sloan Kettering	cbioportal.org/oncoprinter.jsp
ProteinPaint	St Jude	pecan.stjude.org/#/proteinpaint
Circos	Michael Smith Genome Sciences Center	circos.ca
MSigDB	Broad Institute	http://software.broadinstitute.org/gsea/msigdb
CytoScape	National Institute of General Medical Sciences	cytoscape.org
R	The Comprehensive R Archive Network	r-project.org
Other		
Processed DNA copy number profiles	This paper and cited sources	dipg.progenetix.org arraymap.org
Integrated mutation, copy number, expression and methylation data	This paper and cited sources	pedcbioportal.org

CONTACT FOR REAGENT AND RESOURCE SHARING

Further information and requests for resources and reagents should be directed to and will be fulfilled by the Lead Contact, Chris Jones (chris.jones@icr.ac.uk).

EXPERIMENTAL MODEL AND SUBJECT DETAILS

Patient Samples

All new patient material was collected after informed consent and subject to local research ethics committee approval. We collated and profiled 157 unpublished cases of HGG in children and young adults up to the age of 30 years at diagnosis obtained from the Royal Marsden, St Georges and Kings College Hospitals, (n=39, all London, UK), Chinese University of Hong Kong (n=24,

Hong Kong, China), Qilu University Hospital (n=23, Jinan, China), Farhad Hatched Hospital (n=14, Sousse, Tunisia), Federal University of São Paolo (n=14, São Paulo, Brazil), Morozov Children's and Dmitri Rogachev Hospitals (n=12, Moscow, Russia), Queensland Children's Tumor Bank (n=8, Brisbane, Australia), Hospital San Joan de Déu (n=8, Barcelona, Spain), City Hospital #31 (n=6, St Petersburg, Russia), Barretos Cancer Hospital (n=4, Barretos, Brazil), Centre Hospitalier Régional et Universitaire Hautepierre (n=3, Strasbourg, France), and Our Lady Children's Hospital Crumlin (n=2, Dublin, Ireland). A full description of the samples included are provided in Table S1.

METHOD DETAILS

Nucleic Acid Extraction

DNA was extracted from frozen tissue by homogenisation prior to following the DNeasy Blood & Tissue kit protocol (Qiagen, Crawley, UK). DNA was extracted from formalin-fixed, paraffin-embedded (FFPE) pathology blocks after manual macrodissection using the QIAamp DNA FFPE tissue kit protocol (Qiagen). Matched normal DNA was extracted from blood samples using the DNeasy Blood & Tissue kit (Qiagen, Crawley, UK). Concentrations were measured using a Qubit fluorometer (Life Technologies, Paisley, UK). RNA was extracted by following the RNeasy Mini Kit protocol (Qiagen), and quantified on a 2100 Bioanalyzer (Agilent Technologies).

Sanger Sequencing of *H3F3A / HIST1H3B*

PCR for *H3F3A* and *HIST1H3B* was carried out using primers obtained from Life Technologies (Paisley, UK). Products were purified using the QIAquick PCR purification kit (Qiagen), subjected to bidirectional sequencing using BigDye Terminator mix 3.1 (Applied Biosystems, Foster City, CA, USA), with capillary sequencing was done on an ABI 3100 genetic analyzer (Applied Biosystems, Foster City, CA, USA). Sequences were analysed using Mutation Surveyor (SoftGenetics, PN, USA) and manually with 4Peaks (Nucleobytes, Aalsmeer, Netherlands).

Methylation Profiling

50-500 ng DNA was bisulphite-modified and analyzed for genome-wide methylation patterns using the Illumina HumanMethylation450 BeadArray (450k) platform at either the DKFZ or the University College London Genomics Centre, according the manufacturer's instructions. All samples were checked for expected and unexpected genotype matches by pairwise correlation of the 65 genotyping probes on the 450k array.

Exome and RNA Sequencing

50-500 ng DNA was sequenced at the Tumor Profiling Unit, ICR, London, UK using the SureSelect Human All Exon capture sets V4 or V5 (Agilent, Santa Clara, CA, USA), and paired-end-sequenced on an Illumina HiSeq2000 (Illumina, San Diego, CA, USA) with a 100 bp read length. Coverage ranged from 29-295x (median=105x). RNA was sequenced at the ICR Tumor Profiling Unit after SureSelect RNA capture on an Illumina HiSeq2500 with a 125 bp read length.

QUANTIFICATION AND STATISTICAL ANALYSIS

Published Data Sources

These data were combined with those obtained directly from the authors or from public data repositories representing 20 published studies (Barrow et al., 2011; Bax et al., 2010; Buczkowicz et al., 2014; Carvalho et al., 2014; Castel et al., 2015; Fontebasso et al., 2013, 2014; Grasso et al., 2015; International Cancer Genome Consortium PedBrain Tumor Project, 2016; Khuong-Quang et al., 2012; Korshunov et al., 2015; Paugh et al., 2010, 2011; Puget et al., 2012; Schwartzentruber et al., 2012; Sturm et al., 2012; Taylor et al., 2014a; Wu et al., 2012; Wu et al., 2014; Zarghooni et al., 2010) with the following accession numbers: EGA - EGAS00001000226, EGAS0000100192, EGAS00001000575, EGAS00001000720, EGAS00001001139; the Gene Expression Omnibus (www.ncbi.nlm.nih.gov/geo/) - GSE19578, GSE26576, GSE21420, GSE34824, GSE36245, GSE36278 GSE50022, GSE50021, GSE50024, GSE55712; ArrayExpress - E-TABM-857 , E-TABM-1107 . The full cohort included a total of 1254 molecular profiles from 955 samples across 12 platforms, which after quality control and manual annotation to remove duplicates, and supplemented with targeted sequencing of an additional 158 cases, resulted in a total dataset comprised of 1067 individual patients. The full dataset comprises genomic profiles from DNA copy number arrays (Agilent 44K, n=127; Affymetrix 500K, n=100; Affymetrix SNP6.0, n=78; 32k BAC, n=61), Illumina 450k methylation arrays (n=441), whole exome (n=254), genome (n=125), targeted (n=212) and RNA sequencing (n=82), as well as gene expression from Affymetrix U133Plus2 (n=102) and Agilent WG2.5 (n=67) platforms.

DNA Copy Number

DNA copy number data was obtained as array CGH (Agilent 44k and 32K BAC), SNP arrays (Affymetrix 500k and SNP6.0), 450k methylation arrays (Illumina) and/or sequencing data (whole genome and exome). Two color aCGH data was read and normalized using the R packages limma and marray. Log intensity data from Affymetrix SNP arrays was derived using the aroma.affymetrix and aroma.cn package. Combined log_2 intensity data from Illumina 450K methylation arrays was processed using the R packages minfi and conumee. For sequenced samples, coverage of aligned reads was binned into known genes and exons with BEDTools and

log_2 ratios of median coverage in tumor and normal sequences were processed with in-house scripts. To combine copy number platforms, median log_2 ratios were recovered within all known genes and exons and normalized such that the median displacement of X in male:female comparisons was rescaled to an average of -1. Exon-level median log ratios and smoothed values were then combined across platforms and thresholded to call gains and losses above and below log_2 ratios of ±0.3 with a contig of ~1MB and amplifications and deletions above and below a threshold of ±1.5 with a minimum of 3 contiguous exons.

CBS binary segmentation from the DNAcopy package was applied to each dataset to provide smoothed log_2 ratios. Genes within common CNVs in normal individuals were excluded from further analysis with reference to the CNV map of the human genome. DNA copy number data was clustered based upon categorical states (deep deletion, loss, no change, gain and amplification) based upon the Euclidean distance method with a Ward algorithm. Gains and losses in chromosomal arms were called based upon contiguous regions covering more than one third of the exonic regions within each arm. For regions of focal copy number change cases carrying copy number alterations were ranked according to the length of the largest CNA in each case and are plotted as heatmaps aligned to precise genomic coordinates alongside genomic tracks based upon hg19 made with the R package gviz. Minimal regions of copy number alteration were assigned based on the frequency of categorical states within each region. Focal amplifications and deletions were identified in CBS segmented data using the GISTIC algorithm in MATLAB on the exon-level data, with thresholds for gain and loss of 0.3 and gene-level filters to remove regions of common copy number variation in normal individuals based on the CNV map of the human genome.

DNA Methylation

Methylation data from the Illumina Infinium HumanMethylation450 BeadChip was preprocessed using the minfi package in R. DNA copy number was recovered from combined intensities using the conumee package with reference to methylation profiles from normal individuals provided in the CopyNumber450kData package. We have used the Heidelberg brain tumor classifier (molecularneuropathology.org) to assign subtype scores for each tumor compared to 91 different brain tumor entities using a training set built from more than 2000 tumors implemented in the MNP R package. Simplified methylation subgroup assignments were then made to incorporate cases carrying G34R/V or K27M mutations in H3 histones, IDH1 mutation at R132, low grade glioma-like profiles (predominantly diffuse infantile ganglioglioma and pilocytic astrocytoma) and those similar to pleomorphic xanthoastrocytoma (PXA). Wild-type HGG encompassed many other methylation subgroups and were simply assigned by exclusion with the groups above. Clustering of beta values from methylation arrays was performed using the 10K probeset from the Heidelberg classifier based upon Euclidean distance with a ward algorithm. Methylation heatmaps show only the most variable probes of the classifier between simplified methylation subgroups. Overall methylation was calculated as the mean of the 10K classifier probeset for each subgroup and MGMT promoter methylation was calculated based upon the MGMT-SPT27 model implemented in the MNP package. t- stochastic neighbor embedding (tSNE) was used to project the methylation clustering in three dimensions using the Rtsne package. A Pearson correlation matrix of the 10K probeset was subjected to tSNE using a theta value of zero over 10,000 iterations as previously described and plotted using the rgl package.

mRNA Expression

Gene expression data was obtained from Agilent WG2.5, Affymetrix U133Plus2.0 or RNA sequencing platforms. Gene expression was processed from two color Agilent microarrays using the R packages marray and limma and from single channel Affymetrix arrays using the affy package. Differential expression was assigned for microarray data using the limma package based upon a false discovery rate of 5%. RNASeq was aligned with Bowtie2 and TopHat and summarized as gene level fragments per kilobase per million reads sequenced using BEDTools and cufflinks/cuffnorm. Following rlog transformation and normalization, differential expression was assigned with DESeq.2. Known Ensembl genes were further filtered to remove low abundance genes in all three datasets whose maximal expression was within the lowest 20% of all expression values based upon probe intensities or read depth. Replicate probes/features for each gene were removed by selecting those with the greatest median absolute deviation (MAD) in each dataset. Following centering within each dataset, log-transformed expression measures were combined and further normalized using pairwise loess normalization. Gene Set Enrichment Analysis was performed using the GSEA java application based upon pairwise comparisons of the major subgroups in the merged dataset. Heatmaps of gene expression across chromosomal arms were made using centered expression values rescaled across each chromosomal arm based upon the median absolute deviation of each probe. Differential expression analysis of *TOP3A* and *FBXW7* was based on a Mann-Whitney U test of centred expression values between cases with and without losses and amplifications respectively in each case.

Sequence Analysis

Sequencing data was available as whole genome and/or whole exome (predominantly using Agilent's SureSelect whole exome capture sets v4 and v5) Short read sequences from whole exome or whole genome sequencing were aligned to the hg19 assembly of the human genome using bwa. Following duplicate removal with Picard tools variants were called using the Genome Analysis toolkit according to standard Best Practices (Broad) including local re-alignment around Indels, downsampling and variant calling with the Unified Genotyper. Variants were annotated with the variant Effect predictor v74 from Ensembl tools and ANNOVAR to include annotations for variant allele frequency in 1000 genomes dbSNP v132 and the ExAc database as well as functional annotation tools SIFT and Polyphen). Depth of coverage varied from 16-295x (median 88x), with the greatest variation unsurprisingly in the exome data (whole genome range 50-150x, median=85x). Somatic variants were identified in regions covered by at least 10 reads in normal and

tumor sequences carrying at least 3 variant reads in the tumor and less than 2 in normal sequences. Hotspot *TERT* promoter mutations C228T and C250T were incidentally captured by the various exome platforms as they are located only 114 and 146 bp upstream of the translation start site, and were called even if only covered by a few reads. Mutation signatures were ascertained by grouping somatic substitutions on the basis of their 3′ and 5′ bases into 96 possible trinucleotide categories.

Candidate Fusion Gene Nomination

Structural variants were called from whole genome data using Breakdancer (breakdancer.sourceforge.net) filtered to remove commonly multi-mapped regions to identify somatic breakpoints separated by a minimum of 10 kbp involving at least one Ensembl gene. Fusion transcripts were detected from RNAseq data using chimerascan version 0.4.5a filtered to remove common false positives. To minimize unverified false positives, reporting of nominated fusions was restricted to genes within the core functional pathways and processes identified through integrated DNA copy number and somatic variant calling.

Inferred Tumor Purity

We used determined the somatic allele-specific copy number profiles using read depth from whole genome / exome sequencing, and used ASCAT (rick.ac.uk/peter-van-loo/software/ASCAT) to provide for an estimate of the non-neoplastic cell contamination of the sample as well as the overall ploidy of the tumor. Values ranged from 36-100%, with a median of 83%.

Integrated Analysis of Driver Events

Somatic non-synonymous coding mutations were filtered to remove common passenger mutations, polymorphisms and false positives in exome sequencing. Data were integrated with focal DNA copy number calls by GISTIC to provide gene-level binary alteration calls which were further selected for putative drive status on the basis of functional annotation. Oncoprint representations of integrated mutations, gene-level copy number alterations and fusion events were made using the online tool available at cBioportal (cbioportal.org). For the most commonly mutated genes mutations were mapped to the canonical transcript and plotted according to their predicted protein position using the Protein Painter (pecan.stjude.org). Integrated views of copy number alterations, structural variants and somatic mutations were made using CIRCOS (circos.ca) and rearrangements within *TOP3*A amplified regions in whole genome sequenced cases were identified using Breakdancer and aligned with copy number breakpoints in R.

Pathway Analysis

Pathway assignments were made for all genes carrying copy number alterations, structural variations or somatic mutations based on pathways in the MSigDB molecular signatures databases (Broad) as well as Gene Ontologies for Biological Processes and Molecular Functions (Gene Ontology consortium) and canonical pathways from KEGG, NetPath and Reactome. Genes within known CNVs and common false positives in exomic sequencing were excluded with reference to large scale genome profiling studies (CNVmap, ExAc, BCBio) Pathway analysis of genes carrying mutations, gene fusions and copy number aberrations was based on the pathways defined by these combined databases and subjected to enrichment analysis using the EnrichmentMap module within CytoScape.

Statistical Analysis

Statistical analysis was carried out using R 3.3.1 (www.r-project.org). Categorical comparisons of counts were carried out using Fishers exact test, comparisons between groups of continuous variables employed Student's t-test, Wilcoxon signed –rank test, ANOVA or Mann-Whitney U test. Differences in survival were analysed by the Kaplan-Meier method and significance determined by the log-rank test. All tests were two-sided and a p value of less than 0.05 was considered significant. Multiple testing was accounted for using false discovery rate q values or the Bonferroni adjustment.

DATA AND SOFTWARE AVAILABILITY

All newly generated data have been deposited in the European Genome-phenome Archive (www.ebi.ac.uk/ega) with accession number EGAS00001002314 (sequencing) or ArrayExpress (www.ebi.ac.uk/arrayexpress/) with accession number E-MTAB-5528 (450k methylation).

ADDITIONAL RESOURCES

Processed copy number profiles are hosted as a disease-specific project within the Progenetix framework for annotated genomic analyses (dipg.progenetix.org) (Cai et al., 2014), and represented in the arrayMap resource (arraymap.org) (Cai et al., 2012). Curated gene-level copy number and mutation data are provided as part of the pediatric-specific implementation of the cBioPortal genomic data visualisation portal (pedcbioportal.org). Newly-generated raw data files are housed alongside published datasets made available to the Cavatica NIH-integrated cloud platform (www.cavatica.org).

Cancer Cell, Volume *32*

Supplemental Information

Integrated Molecular Meta-Analysis of 1,000 Pediatric High-Grade and Diffuse Intrinsic Pontine Glioma

Alan Mackay, Anna Burford, Diana Carvalho, Elisa Izquierdo, Janat Fazal-Salom, Kathryn R. Taylor, Lynn Bjerke, Matthew Clarke, Mara Vinci, Meera Nandhabalan, Sara Temelso, Sergey Popov, Valeria Molinari, Pichai Raman, Angela J. Waanders, Harry J. Han, Saumya Gupta, Lynley Marshall, Stergios Zacharoulis, Sucheta Vaidya, Henry C. Mandeville, Leslie R. Bridges, Andrew J. Martin, Safa Al-Sarraj, Christopher Chandler, Ho-Keung Ng, Xingang Li, Kun Mu, Saoussen Trabelsi, Dorra H'mida-Ben Brahim, Alexei N. Kisljakov, Dmitry M. Konovalov, Andrew S. Moore, Angel Montero Carcaboso, Mariona Sunol, Carmen de Torres, Ofelia Cruz, Jaume Mora, Ludmila I. Shats, João N. Stavale, Lucas T. Bidinotto, Rui M. Reis, Natacha Entz-Werle, Michael Farrell, Jane Cryan, Darach Crimmins, John Caird, Jane Pears, Michelle Monje, Marie-Anne Debily, David Castel, Jacques Grill, Cynthia Hawkins, Hamid Nikbakht, Nada Jabado, Suzanne J. Baker, Stefan M. Pfister, David T.W. Jones, Maryam Fouladi, André O. von Bueren, Michael Baudis, Adam Resnick, and Chris Jones

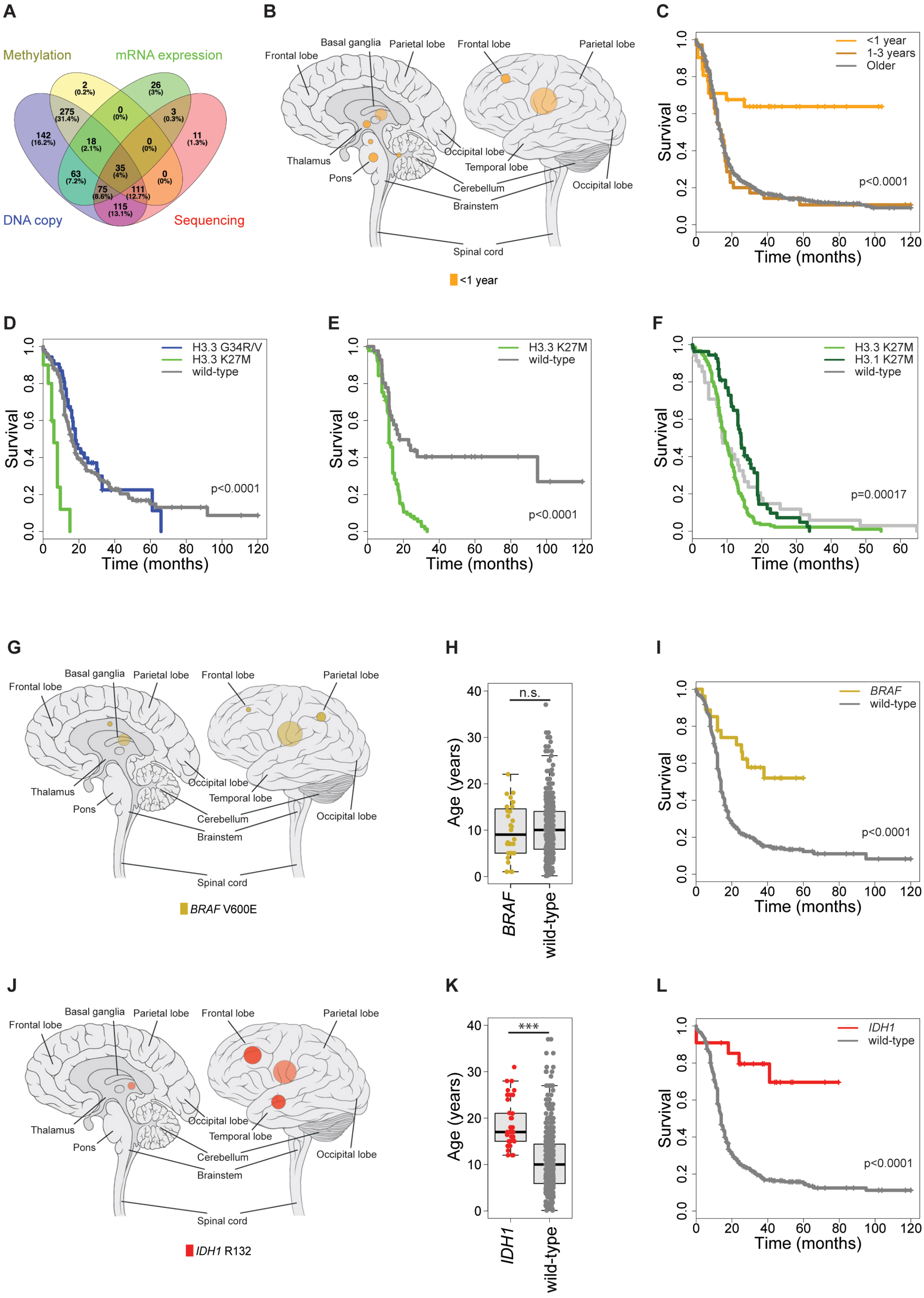
A
Methylation
mRNA expression
DNA copy
Sequencing
B
Basal ganglia
Parietal lobe
Frontal lobe
Thalamus
Pons
Occipital lobe
Temporal lobe
Cerebellum
Brainstem
Spinal cord
<1 year
C
<1 year
1-3 years
Older
p<0.0001
Survival
Time (months)
D
H3.3 G34R/V
H3.3 K27M
wild-type
p<0.0001
E
H3.3 K27M
wild-type
p<0.0001
F
H3.3 K27M
H3.1 K27M
wild-type
p=0.00017
G
BRAF V600E
H
n.s.
Age (years)
BRAF
wild-type
I
BRAF
wild-type
p<0.0001
J
IDH1 R132
K
***
IDH1
wild-type
L
IDH1
wild-type
p<0.0001

Figure S1 (related to Figure 1) – Clinicopathological and molecular subgroups of pHGG/DIPG. (A) Venn diagram showing overlapping molecular data for the cohort. (B) Anatomical location of all cases separated by age at diagnosis <1 year (n=40). Radius of circle is proportional to the number of cases. Left – sagittal section showing internal structures; right – external view highlighting cerebral lobes. Orange = infant. Lighter shaded circles represent a non-specific designation of hemispheric, midline or brainstem. (C) Kaplan-Meier plot of overall survival of cases separated by age at diagnosis <1 year, between 1-3 years, and >3 years of age, p value calculated by the log-rank test (n=818). (D-F) Kaplan-Meier plot of overall survival of cases separated by histone mutation in (D) hemispheric (n=220), (E) midline (n=131), (F) brainstem locations (n=242), p value calculated by the log-rank test. (G) Anatomical location of all cases separated by *BRAF* V600E mutation (n=32, wild-types not shown). Radius of circle is proportional to the number of cases. Left – sagittal section showing internal structures; right – external view highlighting cerebral lobes. Gold = *BRAF* V600E. Lighter shaded circles represent a non-specific designation of hemispheric, midline or brainstem. (H) Boxplot showing age at diagnosis of included cases, separated by *BRAF* V600E mutation (n=553). The lower and upper limits of the boxes represent the first and third quartiles, and the whiskers 1.5x the interquartile range. (I) Kaplan-Meier plot of overall survival of cases separated by *BRAF* V600E, p value calculated by the log-rank test (n=416). (J) Anatomical location of all cases separated by *IDH1* R132 mutations (n=40, wild-types not shown). Radius of circle is proportional to the number of cases. Left – sagittal section showing internal structures; right – external view highlighting cerebral lobes. Red = IDH R132. Lighter shaded circles represent a non-specific designation of hemispheric, midline or brainstem. (K) Boxplot showing age at diagnosis of included cases, separated by *IDH1* R132 mutations (n=672). The thick line within the box is the median, the lower and upper limits of the boxes represent the first and third quartiles, and the whiskers 1.5x the interquartile range. *** p<0.0001, t-test. (L) Kaplan-Meier plot of overall survival of cases separated by *IDH1* R132 mutations, p value calculated by the log-rank test (n=551).

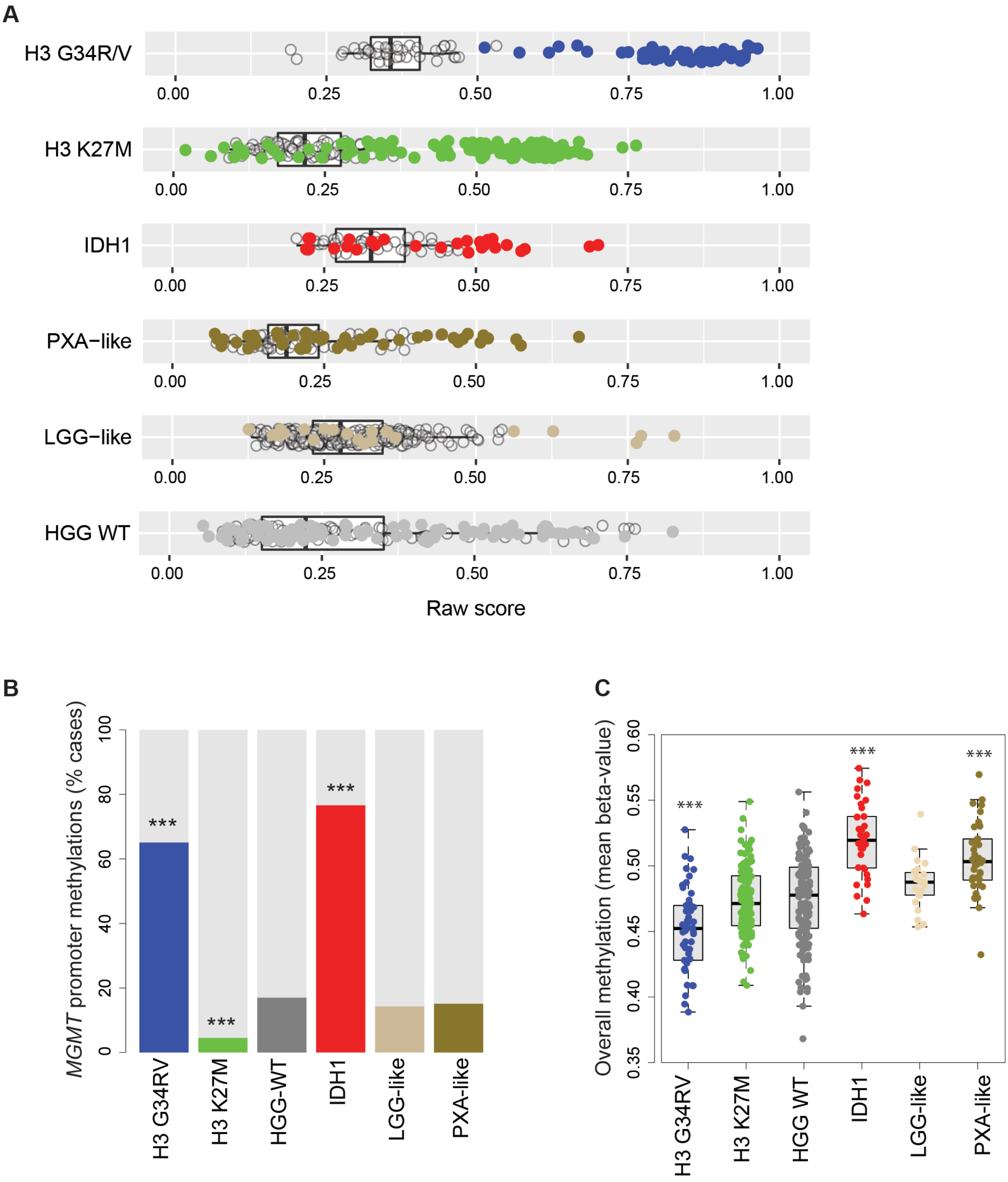

A
H3 G34R/V
H3 K27M
IDH1
PXA−like
LGG−like
HGG WT
0.00
0.25
0.50
0.75
1.00
Raw score
B
MGMT promoter methylations (% cases)
0
20
40
60
80
100
***
H3 G34RV
H3 K27M
HGG-WT
IDH1
LGG-like
PXA-like
C
Overall methylation (mean beta-value)
0.35
0.40
0.45
0.50
0.55
0.60
***
H3 G34RV
H3 K27M
HGG WT
IDH1
LGG-like
PXA-like

Figure S2 (related to Figure 2) – Methylation-based subclassification of pHGG/DIPG. (A) Boxplot of Heidelberg brain tumor subclassifier scores for pediatric HGG in the present study (solid circles, n=441) assigned to one of six HGG subgroups defined by a reference set (open circles). The thick line within the box is the median, the lower and upper limits of the boxes represent the first and third quartiles, and the whiskers 1.5x the interquartile range. H3 G34R/V – blue; H3 K27M – green; IDH1 – red; PXA-like – gold; LGG-like – tan; HGG WT – grey. (B) Barplots of number of cases with methylated *MGMT* promoter, subdivided by methylation subgroup (n=366). (C) Boxplots of mean methylation beta-values for cases subdivided by methylation subgroup (n=441). The thick line within the box is the median, the lower and upper limits of the boxes represent the first and third quartiles, and the whiskers 1.5x the interquartile range. *** $p<0.001$, ** $p<0.01$, *$p<0.05$ *versus* rest, t-test.

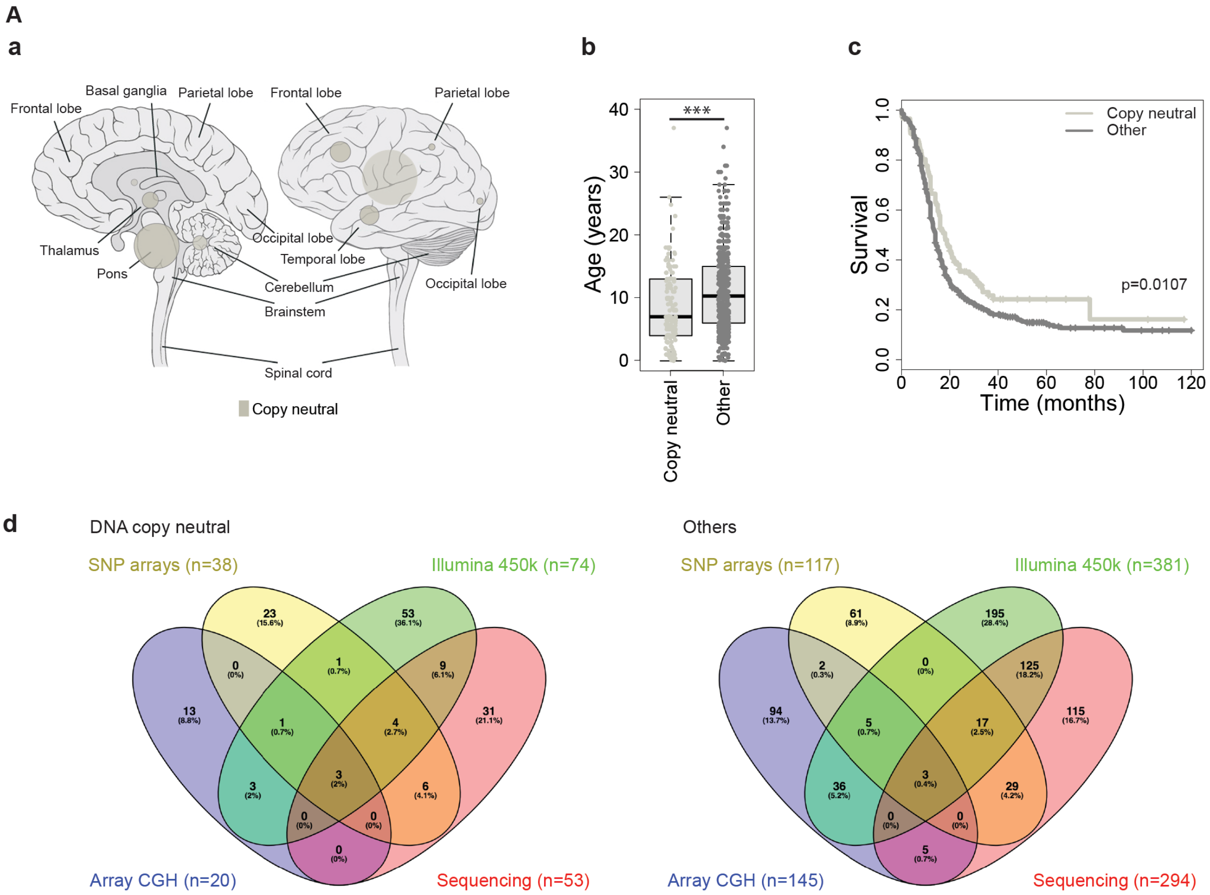
A
a
Frontal lobe
Basal ganglia
Parietal lobe
Frontal lobe
Parietal lobe
Thalamus
Pons
Occipital lobe
Temporal lobe
Cerebellum
Brainstem
Occipital lobe
Spinal cord
Copy neutral
b
***
Age (years)
Copy neutral
Other
c
Copy neutral
Other
Survival
p=0.0107
Time (months)
d
DNA copy neutral
SNP arrays (n=38)
Illumina 450k (n=74)
Array CGH (n=20)
Sequencing (n=53)
Others
SNP arrays (n=117)
Illumina 450k (n=381)
Array CGH (n=145)
Sequencing (n=294)

B

a

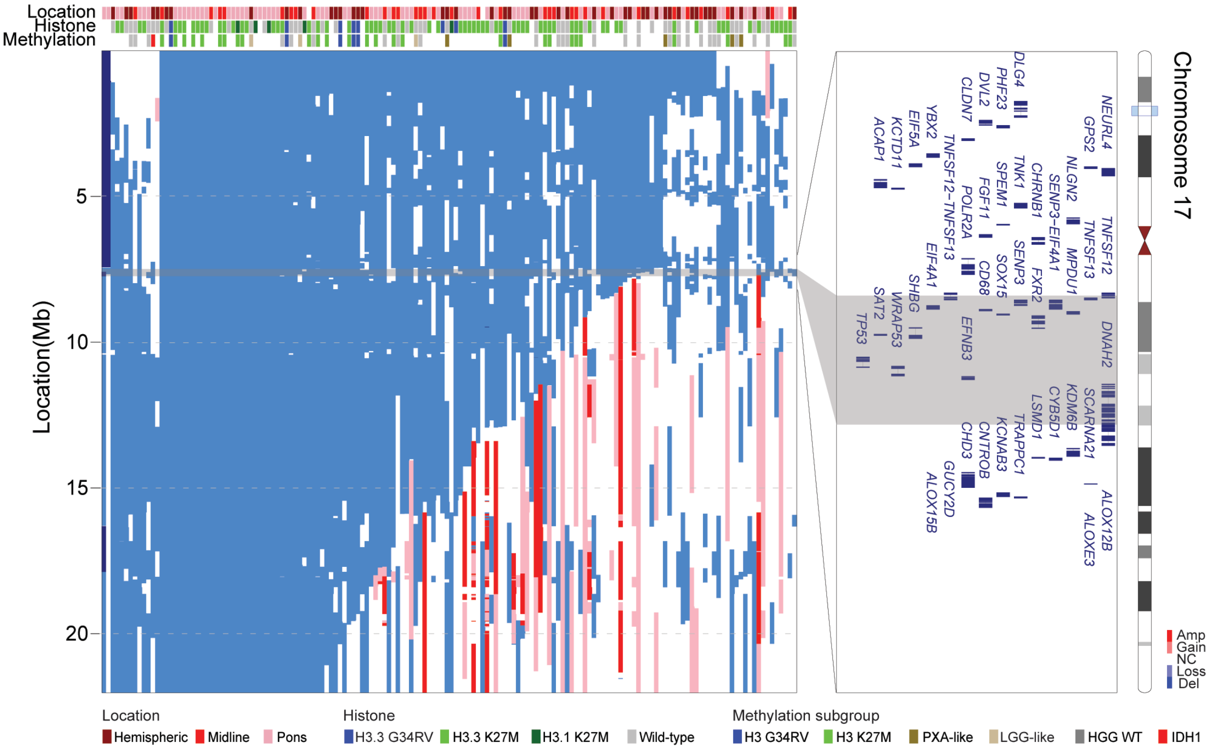

Location
Histone
Methylation
Location(Mb)
5
10
15
20
Chromosome 17
Amp
Gain
NC
Loss
Del
Location
Hemispheric
Midline
Pons
Histone
H3.3 G34RV
H3.3 K27M
H3.1 K27M
Wild-type
Methylation subgroup
H3 G34RV
H3 K27M
PXA-like
LGG-like
HGG WT
IDH1

b

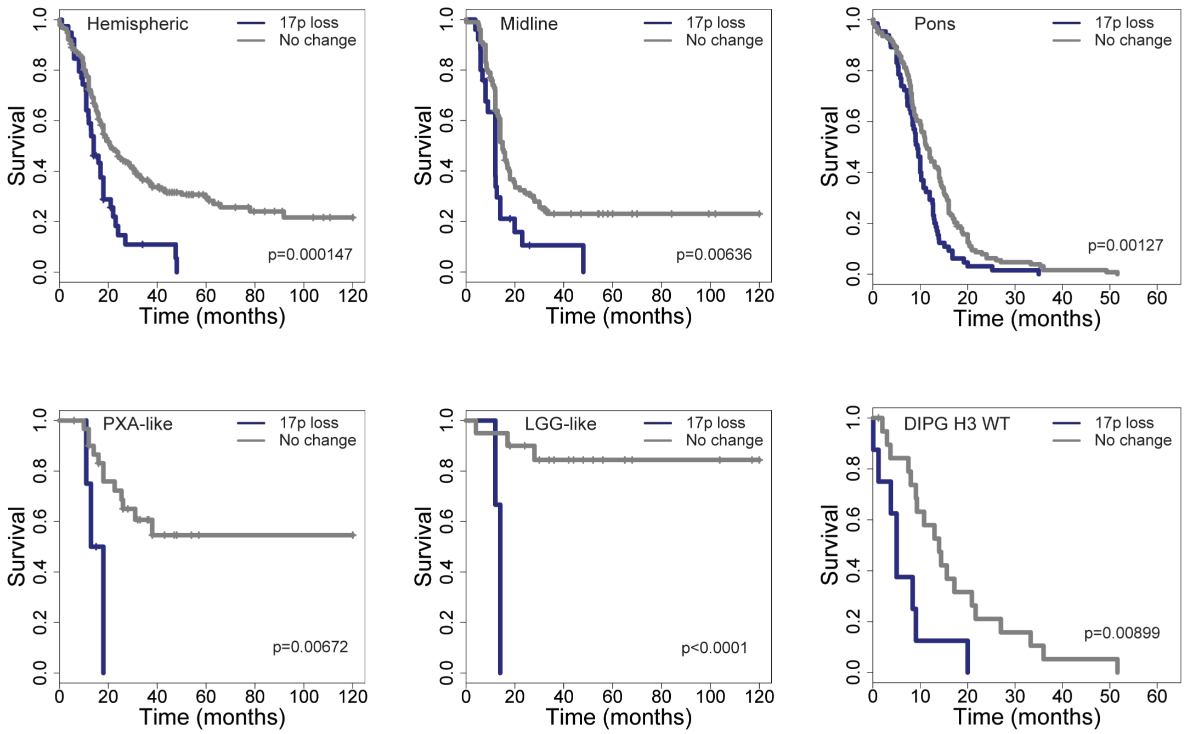

Hemispheric
17p loss
No change
p=0.000147
Midline
p=0.00636
Pons
p=0.00127
PXA-like
p=0.00672
LGG-like
p<0.0001
DIPG H3 WT
p=0.00899
Survival
Time (months)

C

a

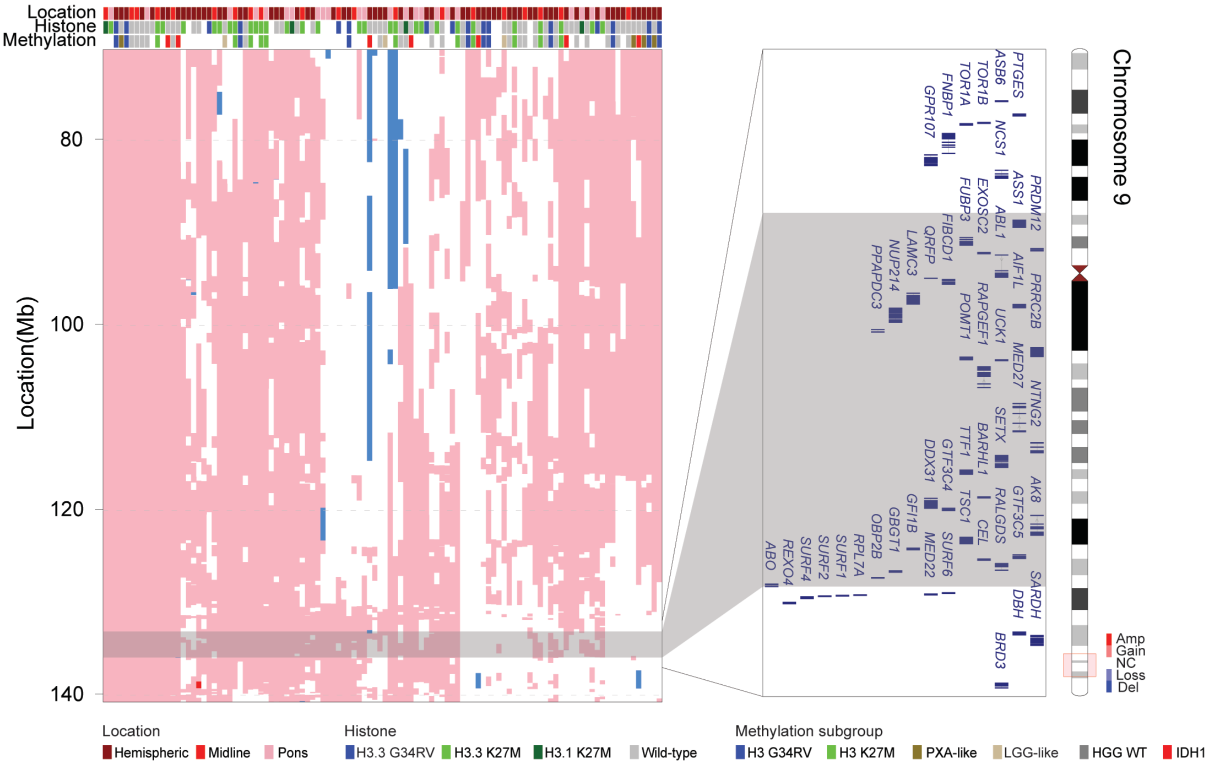
Location
Histone
Methylation
Location(Mb)
80
100
120
140
Chromosome 9
Amp
Gain
NC
Loss
Del
Location
Hemispheric
Midline
Pons
Histone
H3.3 G34RV
H3.3 K27M
H3.1 K27M
Wild-type
Methylation subgroup
H3 G34RV
H3 K27M
PXA-like
LGG-like
HGG WT
IDH1

b

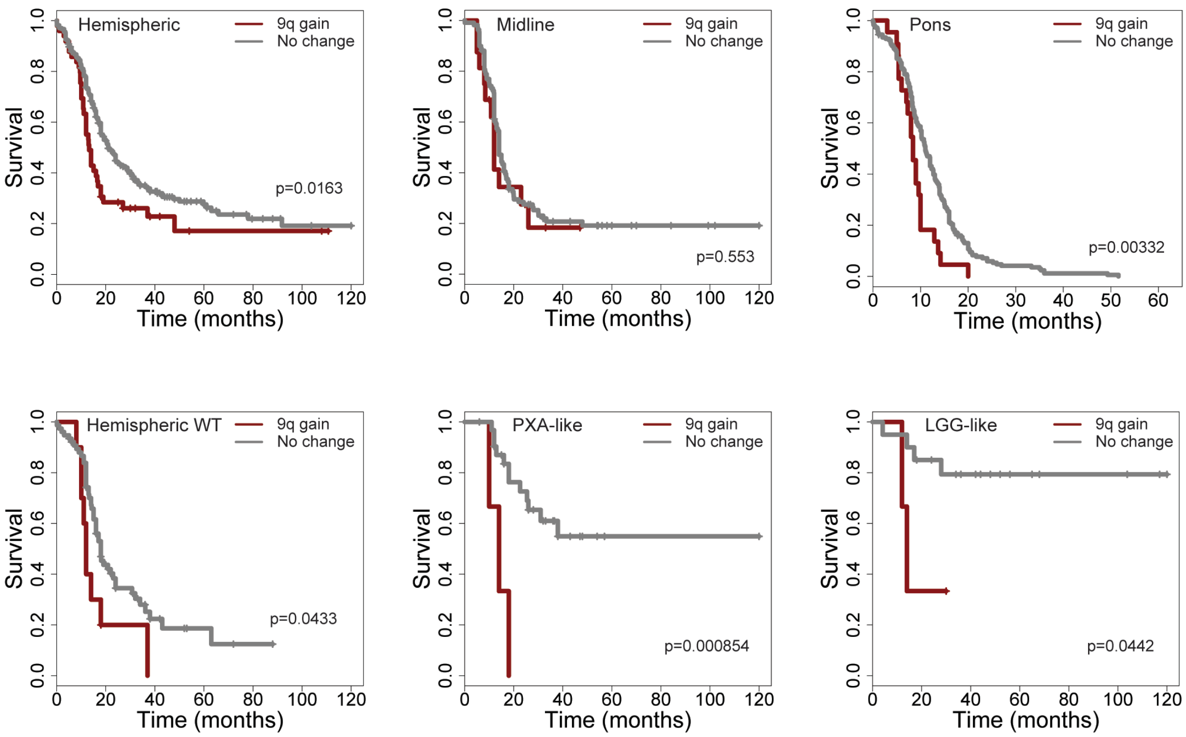
Hemispheric
9q gain
No change
p=0.0163
Midline
p=0.553
Pons
p=0.00332
Hemispheric WT
p=0.0433
PXA-like
p=0.000854
LGG-like
p=0.0442
Survival
Time (months)

D

a

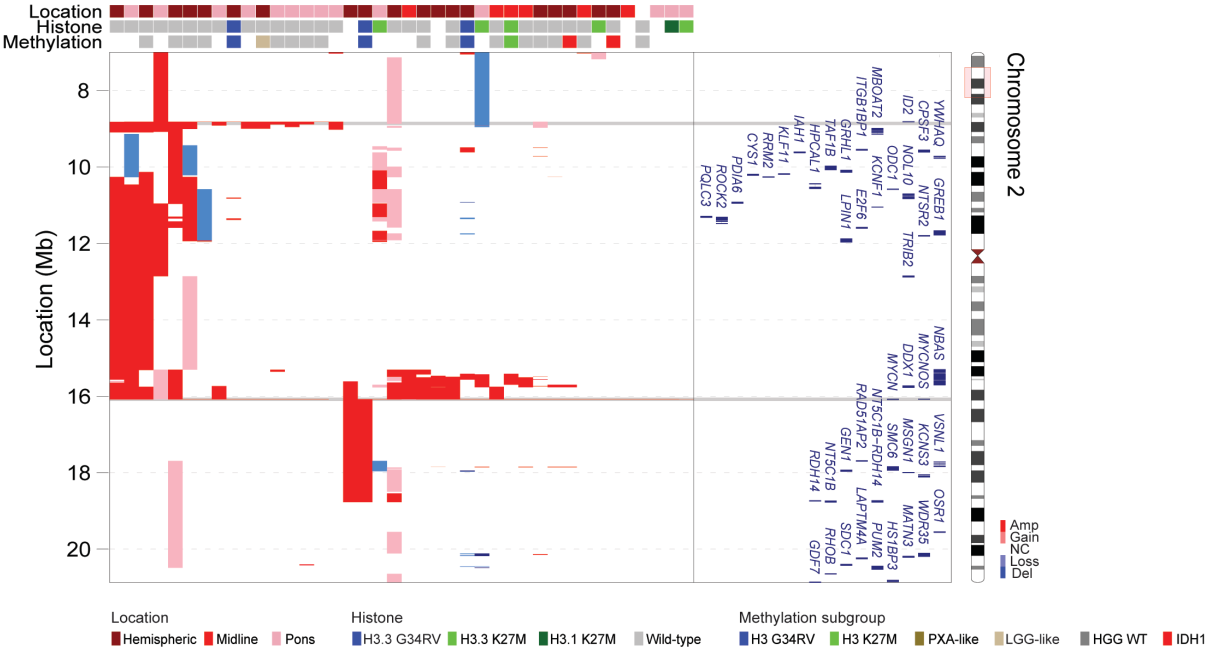

Location
Histone
Methylation
Location (Mb)
Chromosome 2
Amp
Gain
NC
Loss
Del
Location
Hemispheric
Midline
Pons
Histone
H3.3 G34RV
H3.3 K27M
H3.1 K27M
Wild-type
Methylation subgroup
H3 G34RV
H3 K27M
PXA-like
LGG-like
HGG WT
IDH1

b

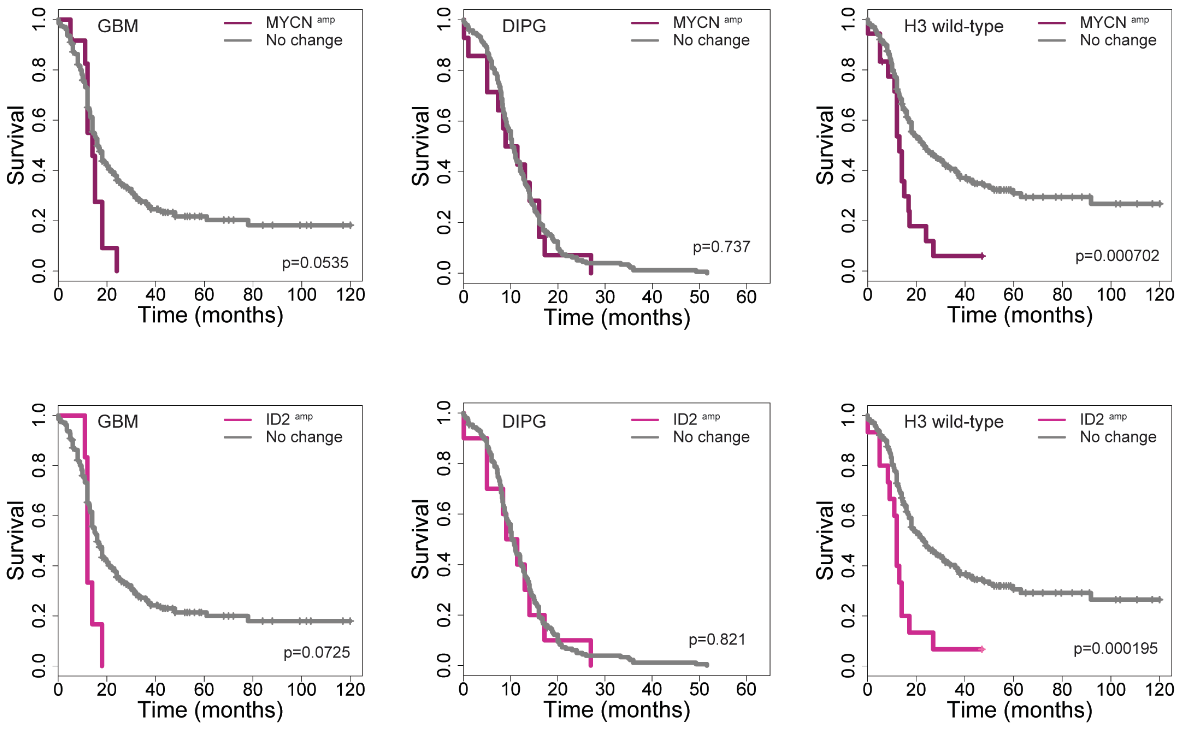

GBM
DIPG
H3 wild-type
MYCN amp
ID2 amp
No change
Survival
Time (months)
p=0.0535
p=0.737
p=0.000702
p=0.0725
p=0.821
p=0.000195

E

a

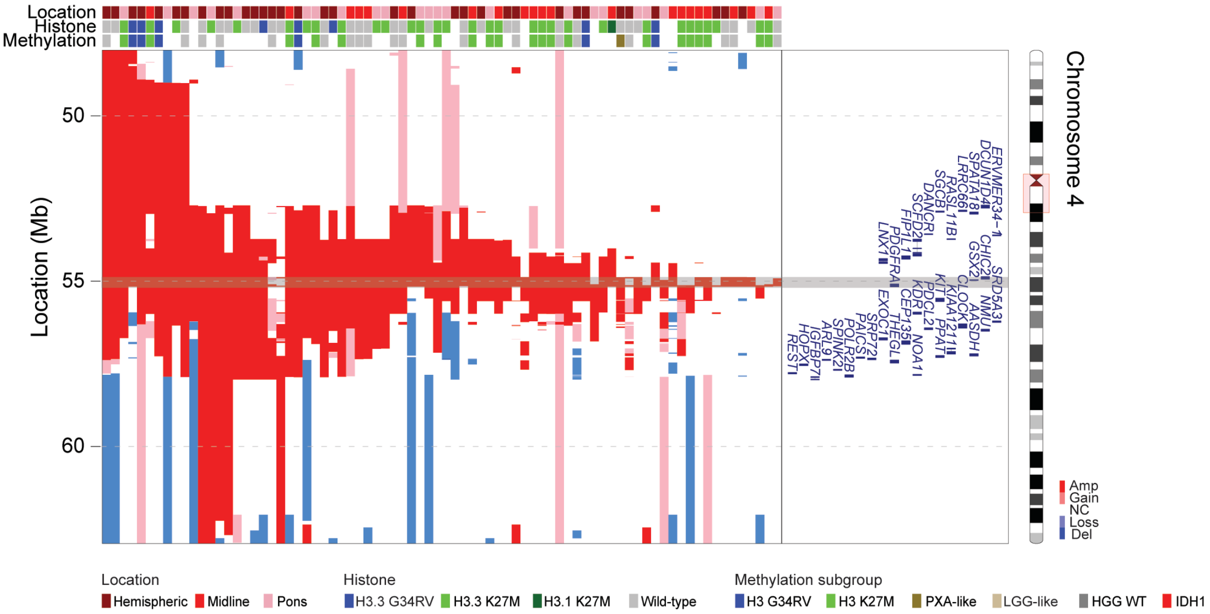

Location
Histone
Methylation
Location (Mb)
50
55
60
Chromosome 4
Amp
Gain
NC
Loss
Del
Location
Hemispheric
Midline
Pons
Histone
H3.3 G34RV
H3.3 K27M
H3.1 K27M
Wild-type
Methylation subgroup
H3 G34RV
H3 K27M
PXA-like
LGG-like
HGG WT
IDH1

b

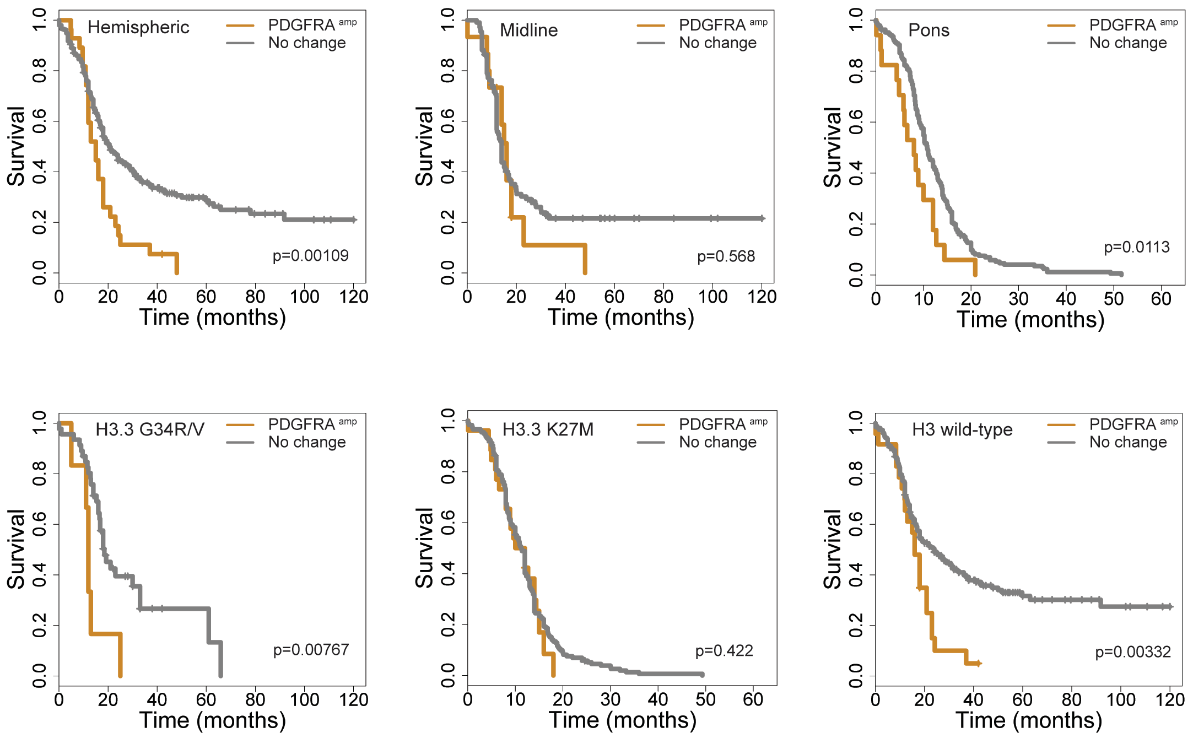

Hemispheric
PDGFRA amp
No change
p=0.00109
Midline
p=0.568
Pons
p=0.0113
H3.3 G34R/V
p=0.00767
H3.3 K27M
p=0.422
H3 wild-type
p=0.00332
Survival
Time (months)

F

a

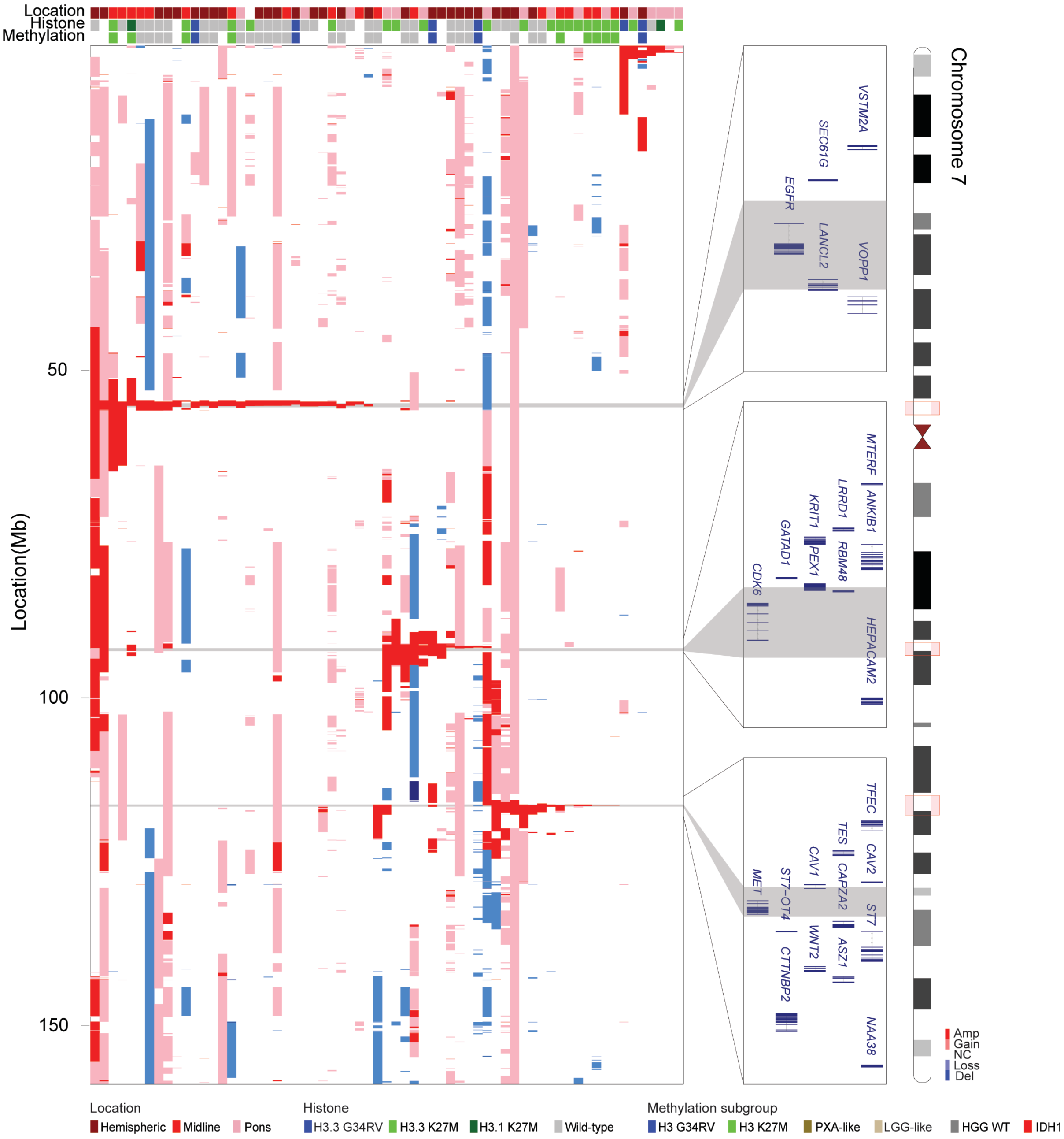

Location
Histone
Methylation
Location(Mb)
50
100
150
VSTM2A
SEC61G
EGFR
LANCL2
VOPP1
MTERF
ANKIB1
LRRD1
KRIT1
RBM48
GATAD1
PEX1
CDK6
HEPACAM2
TFEC
TES
CAV2
CAV1
CAPZA2
ST7-OT4
MET
ST7
WNT2
ASZ1
CTTNBP2
NAA38
Chromosome 7
Amp
Gain
NC
Loss
Del
Location
Hemispheric
Midline
Pons
Histone
H3.3 G34RV
H3.3 K27M
H3.1 K27M
Wild-type
Methylation subgroup
H3 G34RV
H3 K27M
PXA-like
LGG-like
HGG WT
IDH1

b

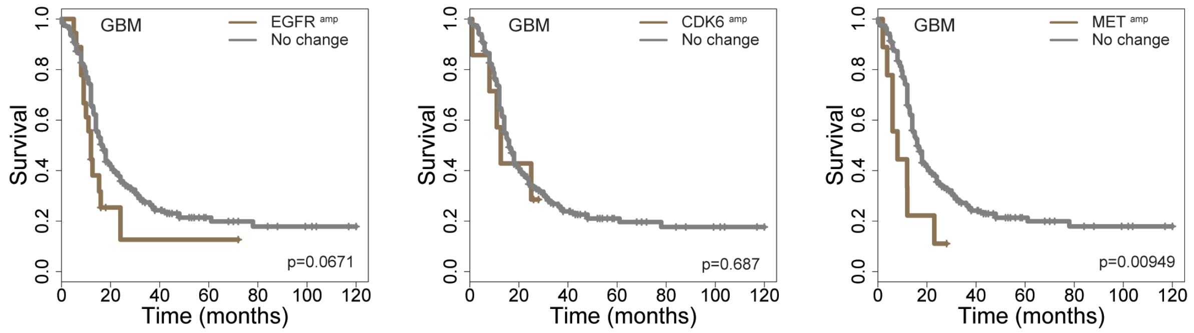

GBM
EGFR amp
No change
p=0.0671
GBM
CDK6 amp
No change
p=0.687
GBM
MET amp
No change
p=0.00949
Survival
0.0
0.2
0.4
0.6
0.8
1.0
0
20
40
60
80
100
120
Time (months)

G

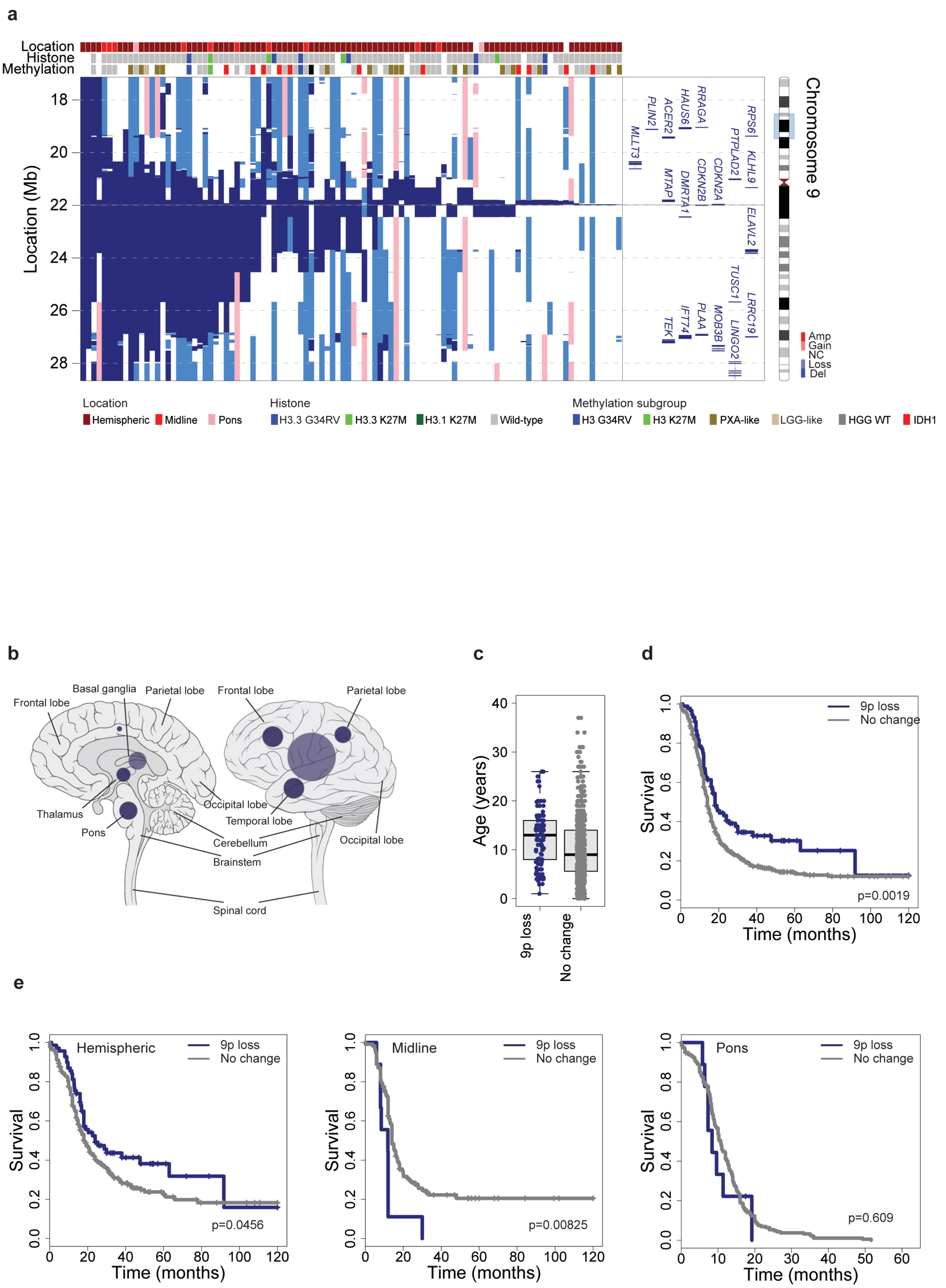
a
Location
Histone
Methylation
Location (Mb)
Chromosome 9
Amp
Gain
NC
Loss
Del
Location
Hemispheric
Midline
Pons
Histone
H3.3 G34RV
H3.3 K27M
H3.1 K27M
Wild-type
Methylation subgroup
H3 G34RV
H3 K27M
PXA-like
LGG-like
HGG WT
IDH1
b
Frontal lobe
Basal ganglia
Parietal lobe
Thalamus
Pons
Occipital lobe
Temporal lobe
Cerebellum
Brainstem
Spinal cord
c
Age (years)
9p loss
No change
d
Survival
Time (months)
9p loss
No change
p=0.0019
e
Hemispheric
p=0.0456
Midline
p=0.00825
Pons
p=0.609

Figure S3 (related to Figure 3) – DNA copy number aberrations in pHGG/DIPG. (A) DNA copy neutral cases. (a) Anatomical location (n=147). Radius of circle is proportional to the number of cases. Left – sagittal section showing internal structures; right – external view highlighting cerebral lobes. Pale grey = copy neutral cases. Lighter shaded circles represent a non-specific designation of hemispheric, midline or brainstem. (b) Boxplot showing age at diagnosis of included cases, separated by DNA copy neutral cases (n=798). The thick line within the box is the median, the lower and upper limits of the boxes represent the first and third quartiles, and the whiskers 1.5x the interquartile range. *** $p<0.0001$, t-test. (c) Kaplan-Meier plot of overall survival of cases separated by DNA copy neutral cases, p value calculated by the log-rank test (n=638). (d) Venn diagrams showing platform distribution of copy neutral (left, n=147) and other cases (right, n=687). (B-G) Recurrent copy number changes. (a) Segmented exon-level DNA copy number heatmaps for (B) 17p loss (n=156), (C) 9q gain (n=108), (D) 2p24 amplification (n=42), (E) 4q12 amplification (n=77), (F) chromosome 7 amplifications (n=59), (G) 9p21 deletion (n=102). Dark red, amplification; red, gain; dark blue, deletion; blue, loss. Chromosomal ideograms are provided indicating enlarged genome browser view and genes within common regions targeted across samples (grey). Clinicopathological and molecular annotations are provided as bars according to the included key. (B-F) (b) Kaplan-Meier plot of overall survival of subgroups of cases separated by indicated DNA copy number change, p value calculated by the log-rank test. (G) 9p loss. (b) Anatomical location (n=115). Radius of circle is proportional to the number of cases. Left – sagittal section showing internal structures; right – external view highlighting cerebral lobes. Dark blue = 9p loss cases. Lighter shaded circles represent a non-specific designation of hemispheric, midline or brainstem. (c) Boxplot showing age at diagnosis of included cases, separated by 9p loss cases (n=798). The thick line within the box is the median, the lower and upper limits of the boxes represent the first and third quartiles, and the whiskers 1.5x the interquartile range. *** $p<0.0001$, t-test. (d,e) Kaplan-Meier plot of overall survival of cases separated by 9p loss cases, p value calculated by the log-rank test (n=659).

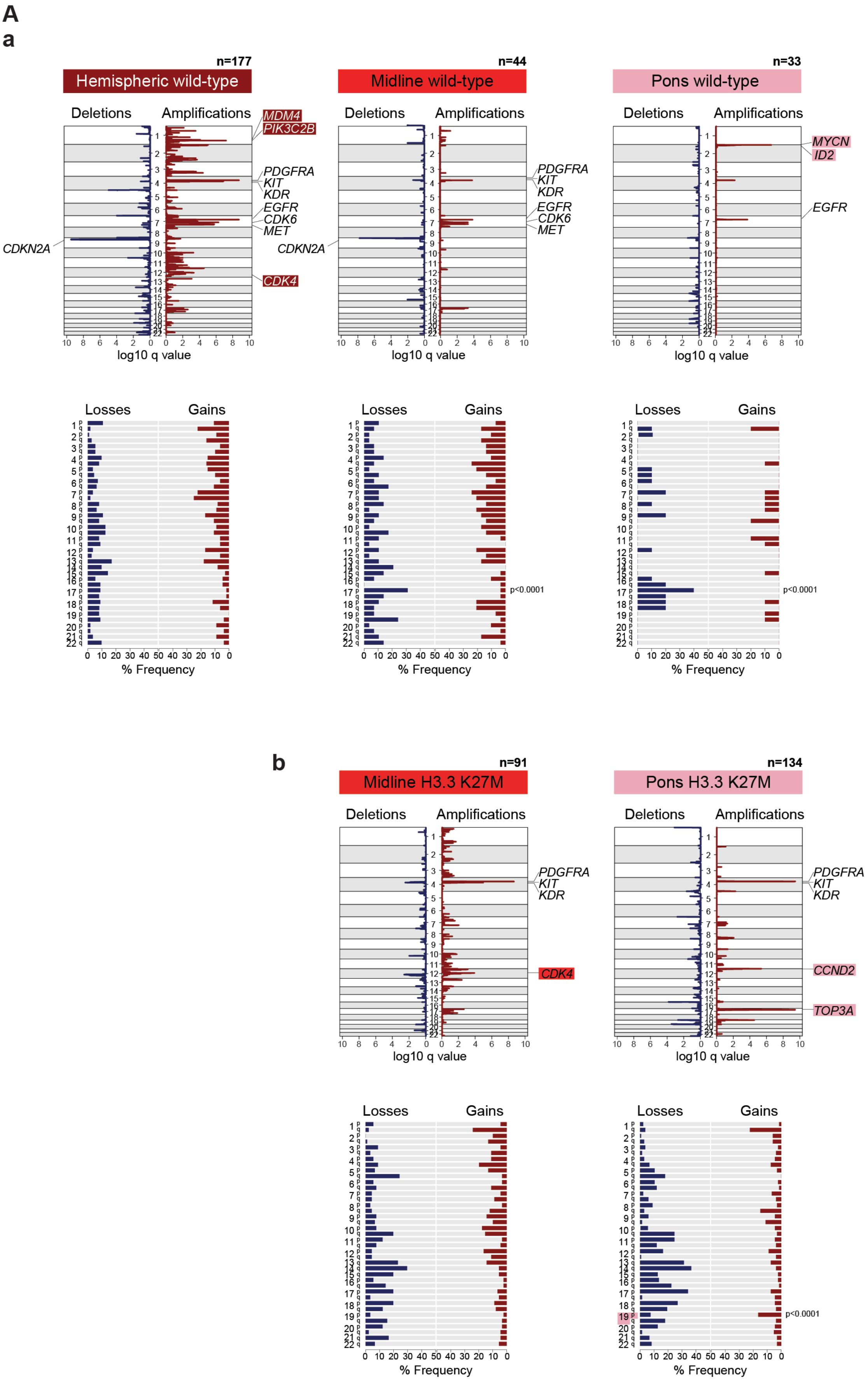
A
a
n=177
Hemispheric wild-type
n=44
Midline wild-type
n=33
Pons wild-type
Deletions
Amplifications
MDM4
PIK3C2B
PDGFRA
KIT
KDR
EGFR
CDK6
MET
CDKN2A
CDK4
MYCN
ID2
log10 q value
Losses
Gains
% Frequency
p<0.0001
b
n=91
Midline H3.3 K27M
n=134
Pons H3.3 K27M
CCND2
TOP3A

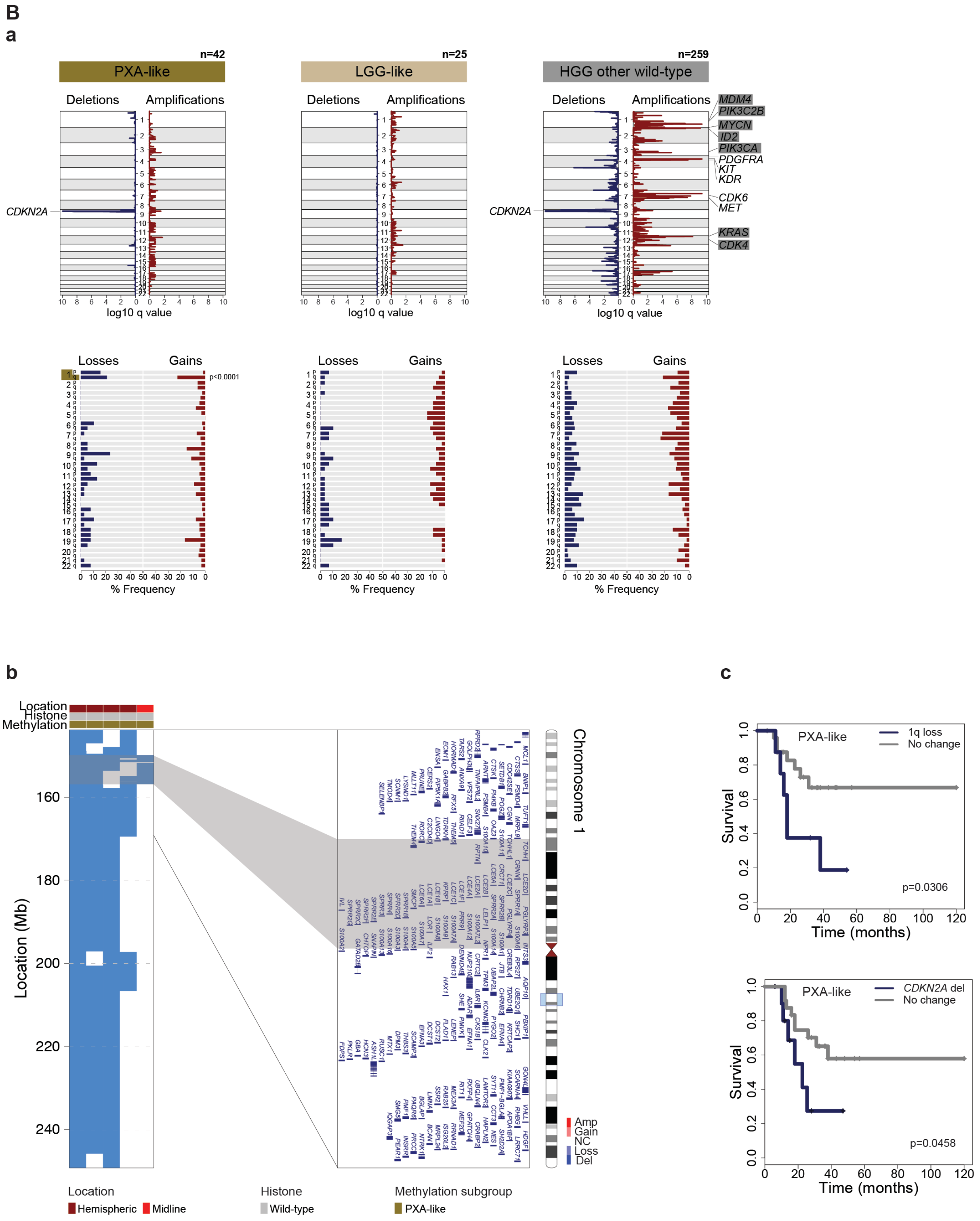

B
a
PXA-like
n=42
LGG-like
n=25
HGG other wild-type
n=259
Deletions
Amplifications
CDKN2A
MDM4
PIK3C2B
MYCN
ID2
PIK3CA
PDGFRA
KIT
KDR
CDK6
MET
KRAS
CDK4
log10 q value
Losses
Gains
p<0.0001
% Frequency
b
Location
Histone
Methylation
Location (Mb)
Chromosome 1
Amp
Gain
NC
Loss
Del
Location
Hemispheric
Midline
Histone
Wild-type
Methylation subgroup
PXA-like
c
PXA-like
1q loss
No change
Survival
p=0.0306
Time (months)
CDKN2A del
No change
p=0.0458

C

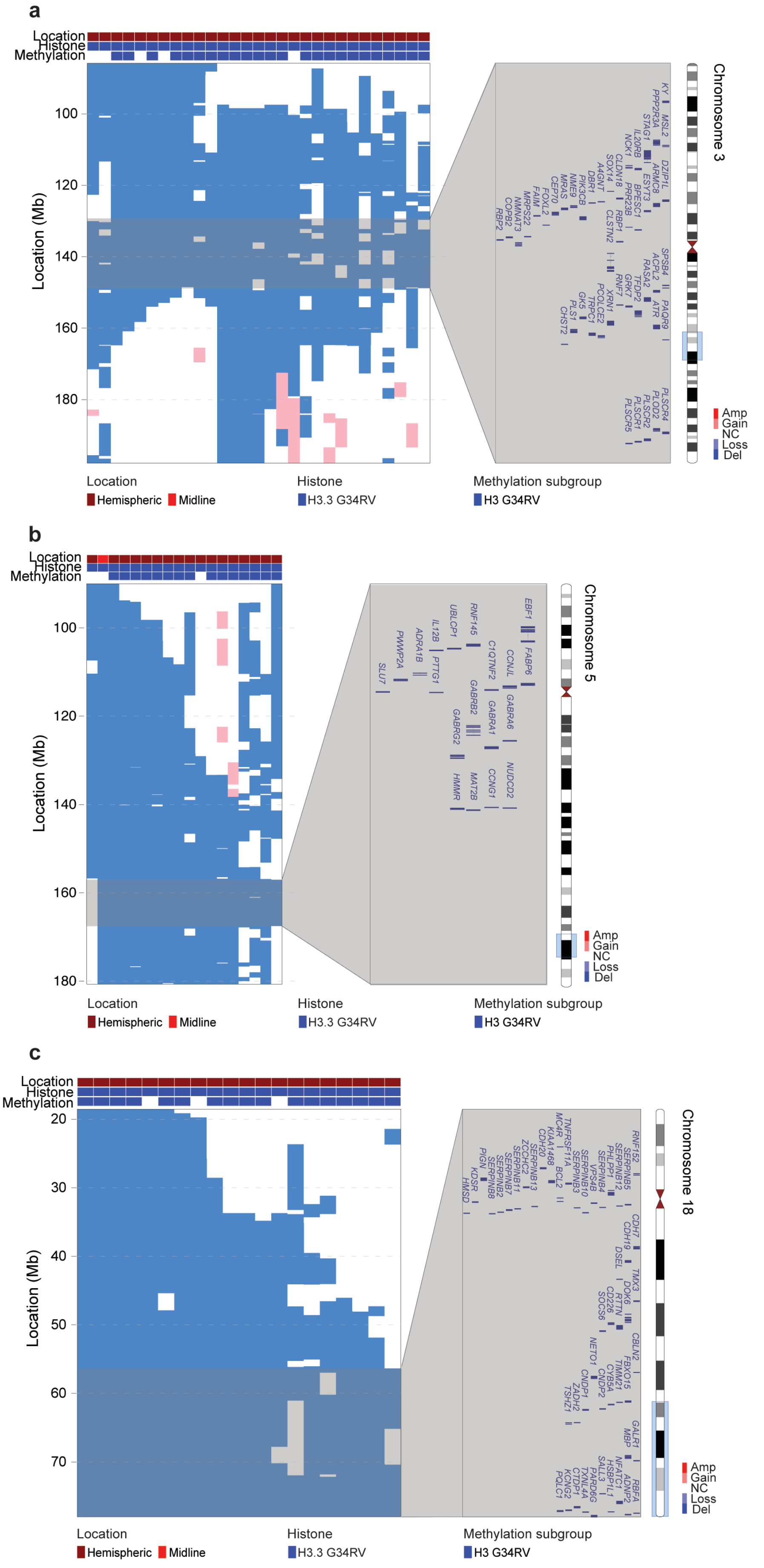
a
Location
Histone
Methylation
Location (Mb)
100
120
140
160
180
Chromosome 3
Amp
Gain
NC
Loss
Del
Location
Hemispheric
Midline
Histone
H3.3 G34RV
Methylation subgroup
H3 G34RV
b
Location
Histone
Methylation
Location (Mb)
100
120
140
160
180
Chromosome 5
Amp
Gain
NC
Loss
Del
Location
Hemispheric
Midline
Histone
H3.3 G34RV
Methylation subgroup
H3 G34RV
c
Location
Histone
Methylation
Location (Mb)
20
30
40
50
60
70
Chromosome 18
Amp
Gain
NC
Loss
Del
Location
Hemispheric
Midline
Histone
H3.3 G34RV
Methylation subgroup
H3 G34RV

Figure S4 (related to Figure 4) – Subgroup-specific copy number changes in pHGG/DIPG. (A) GISTIC and frequency barplots for whole chromosomal arm changes for (a) histone wild-type cases and (b) H3.3K27M cases subdivided by anatomical location. Log_{10} values for GISTIC are plotted across the genome for both amplifications (dark red) and deletions (dark blue), with significantly enriched events labelled by likely driver genes. Subgroup-specific genes are highlighted by the appropriate color, as are significantly enriched whole arm alterations (p<0.0001, Fishers exact test). (B) Histone wild-type cases. (b) GISTIC and frequency barplots for whole chromosomal arm changes subdivided by simplified methylation subclass. Log_{10} values for GISTIC are plotted across the genome for both amplifications (dark red) and deletions (dark blue), with significantly enriched events labelled by likely driver genes. Subgroup-specific genes are highlighted by the appropriate color, as are significantly enriched whole arm alterations (p<0.0001, Fishers exact test). (b) Segmented exon-level DNA copy number heatmaps for 1q loss in PXA-like cases (dark red, amplification; red, gain; dark blue, deletion; blue, loss; n=5). Chromosomal ideograms are provided indicating enlarged genome browser view and genes within common regions targeted across samples (grey). Clinicopathological and molecular annotations are provided as bars according to the included key. (c) Kaplan-Meier plot of overall survival of subgroups of cases separated by indicated DNA copy number change in PXA-like cases (n=35), p value calculated by the log-rank test. (C) Copy number losses in H3.3G34R/V cases. (a) 3q loss (n=29). (b) 5q loss (n=18). (c) 18q loss (n=20). Segmented exon-level DNA copy number heatmaps for losses in H3.3G34R/V cases (dark red, amplification; red, gain; dark blue, deletion; blue, loss). Chromosomal ideograms are provided indicating enlarged genome browser view and genes within common regions targeted across samples (grey). Clinicopathological and molecular annotations are provided as bars according to the included key.

A

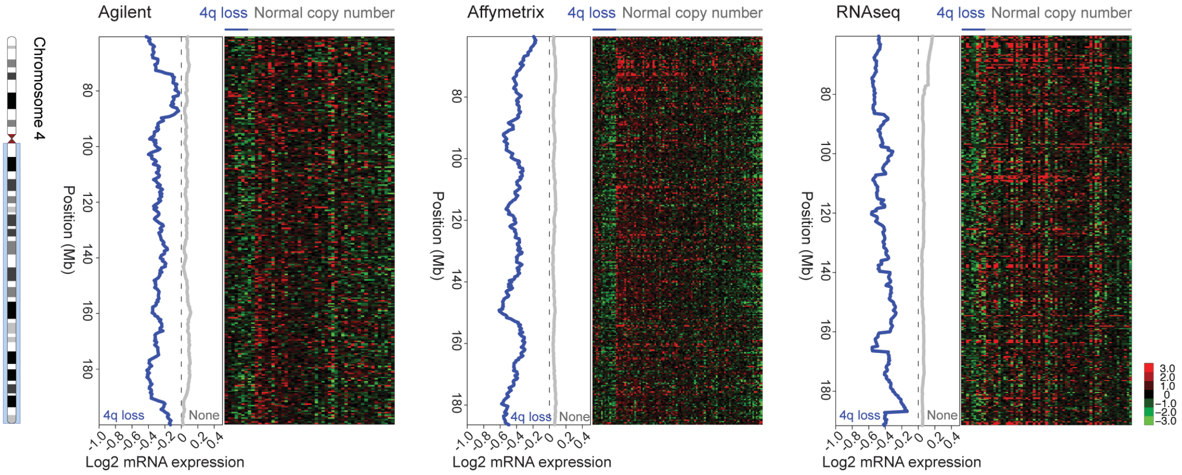
Chromosome 4
Agilent
Affymetrix
RNAseq
4q loss
Normal copy number
Position (Mb)
4q loss
None
Log2 mRNA expression
3.0
2.0
1.0
0
−1.0
−2.0
−3.0

B

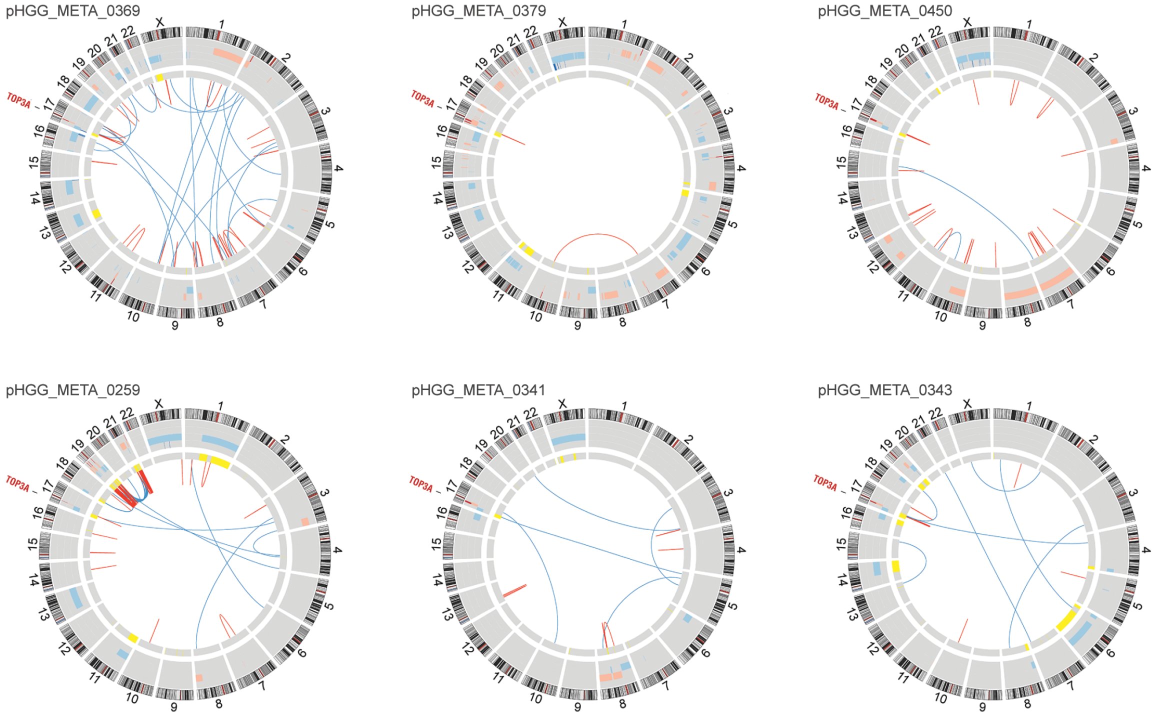
pHGG_META_0369
pHGG_META_0379
pHGG_META_0450
pHGG_META_0259
pHGG_META_0341
pHGG_META_0343
TOP3A

Figure S5 (related to Figure 5) – Alterations targeting *FBXW7* in H3.3G34R/V pHGG and *TOP3A* in H3.3K27M DIPG. (A) Gene expression heatmap for all genes on chromosome 4q across three independent platforms, separated by those with 4q loss (blue) and those with normal copy number (grey). Agilent, left (n=67); Affymetrix, middle (n=102); RNAseq, right (n=82). (B) CIRCOS plots for 6 cases (5x H3.3K27M DIPG, 1x midline histone wild-type) with complex rearrangements leading to *TOP3A* amplification. In each case, plots provide DNA copy number changes (dark red, amplification; red, gain; dark blue, deletion; blue, loss) and loss of heterozygosity (yellow) on the inner rings, and intra- (orange) and inter- (blue) chromosomal translocations inside the circle.

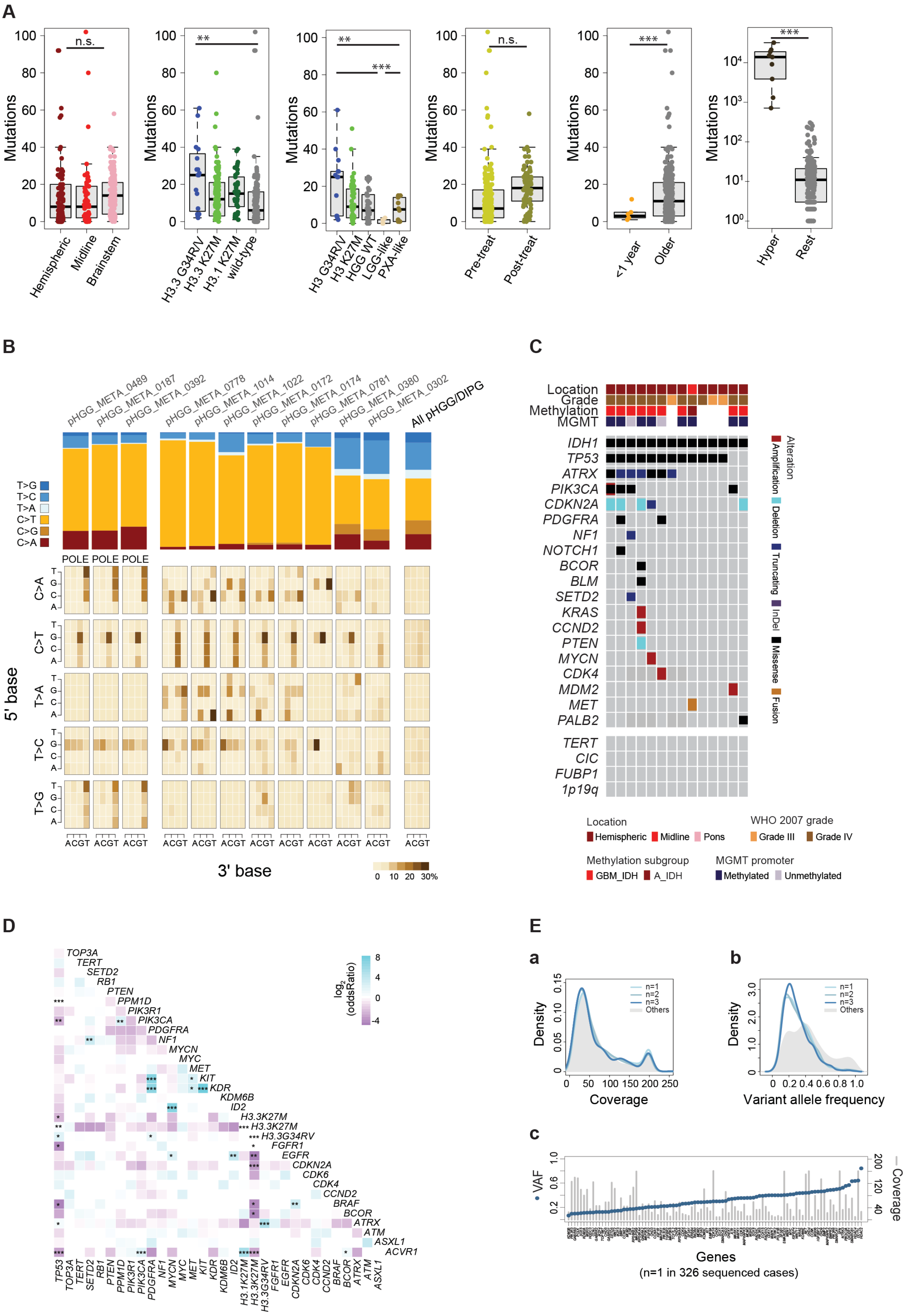
A
Mutations
n.s.
Hemispheric
Midline
Brainstem
**
H3.3 G34R/V
H3.3 K27M
H3.1 K27M
wild-type
***
H3 G34R/V
H3 K27M
HGG WT
LGG-like
PXA-like
Pre-treat
Post-treat
<1 year
Older
Hyper
Rest
B
pHGG_META_0489
pHGG_META_0187
pHGG_META_0392
pHGG_META_0778
pHGG_META_1014
pHGG_META_1022
pHGG_META_0172
pHGG_META_0174
pHGG_META_0781
pHGG_META_0380
pHGG_META_0302
All pHGG/DIPG
T>G
T>C
T>A
C>T
C>G
C>A
POLE POLE POLE
5' base
3' base
ACGT
0 10 20 30%
C
Location
Grade
Methylation
MGMT
IDH1
TP53
ATRX
PIK3CA
CDKN2A
PDGFRA
NF1
NOTCH1
BCOR
BLM
SETD2
KRAS
CCND2
PTEN
MYCN
CDK4
MDM2
MET
PALB2
TERT
CIC
FUBP1
1p19q
Alteration
Amplification
Deletion
Truncating
InDel
Missense
Fusion
Location
Hemispheric
Midline
Pons
WHO 2007 grade
Grade III
Grade IV
Methylation subgroup
GBM_IDH
A_IDH
MGMT promoter
Methylated
Unmethylated
D
TOP3A
TERT
SETD2
RB1
PTEN
PPM1D
PIK3R1
PIK3CA
PDGFRA
NF1
MYCN
MYC
MET
KIT
KDR
KDM6B
ID2
H3.1K27M
H3.3K27M
H3.3G34RV
FGFR1
EGFR
CDKN2A
CDK6
CDK4
CCND2
BRAF
BCOR
ATRX
ATM
ASXL1
ACVR1
TP53
log2 (oddsRatio)
E
a
Density
Coverage
n=1
n=2
n=3
Others
b
Variant allele frequency
c
VAF
Coverage
Genes
(n=1 in 326 sequenced cases)

Figure S6 (related to Figure 6) – Somatic mutations in pHGG/DIPG. (A) Mutation rate boxplots across non-hypermutator pHGG/DIPG separated according to location (n=318), histone mutation(n=326), methylation subgroup (n=129), pre/post-treatment sampling (n=326) and age <1 year (n=326), and then compared to 11 hypermutator cases (plotted on a $\log_{10}$ scale). The thick line within the box is the median, the lower and upper limits of the boxes represent the first and third quartiles, and the whiskers 1.5x the interquartile range. *** adjusted p<0.0001, **<0.01, t-test. (B) Mutation signatures for the 11 hypermutator cases. (Top) Simple stacked barplot representation of the proportion of mutation types observed in individual hypermutator cases and the remaining accumulated dataset. Base changes given in the key. (Bottom) Mutation context given for each of the 96 mutated trinucleotides, represented by heatmap. The base located 5′ to each mutated base is shown on the vertical axis, and the 3′ base is on the horizontal axis. (C) Oncoprint representation of an integrated annotation of somatic mutations and DNA copy number changes in *IDH1* mutant cases (n=14). Clinicopathological and molecular annotations are provided as bars according to the included key. (D) Mutual exclusivity analysis. Pairwise Fishers exact tests are calculated for each of the 30 most commonly altered genes in pHGG/DIPG. Log_2-transformed odds ratios are plotted in a purple (negative correlation) to cyan (positive correlation) color scheme. White boxes represent no co-occuring events. *** adjusted p<0.001, ** adjusted p<0.01, *adjusted p<0.05. (E) Coverage and variant allele frequency (VAF) statistics for rare variants. (a) Coverage and (b) VAF are provided as a density plot for those variants detected in 1-3 cases (blue lines) overlaid with the remaining variants (shaded grey). (c) Co-plot of VAF (blue points) and coverage (grey bars) for variants detected in a single case, ordered by VAF.

A

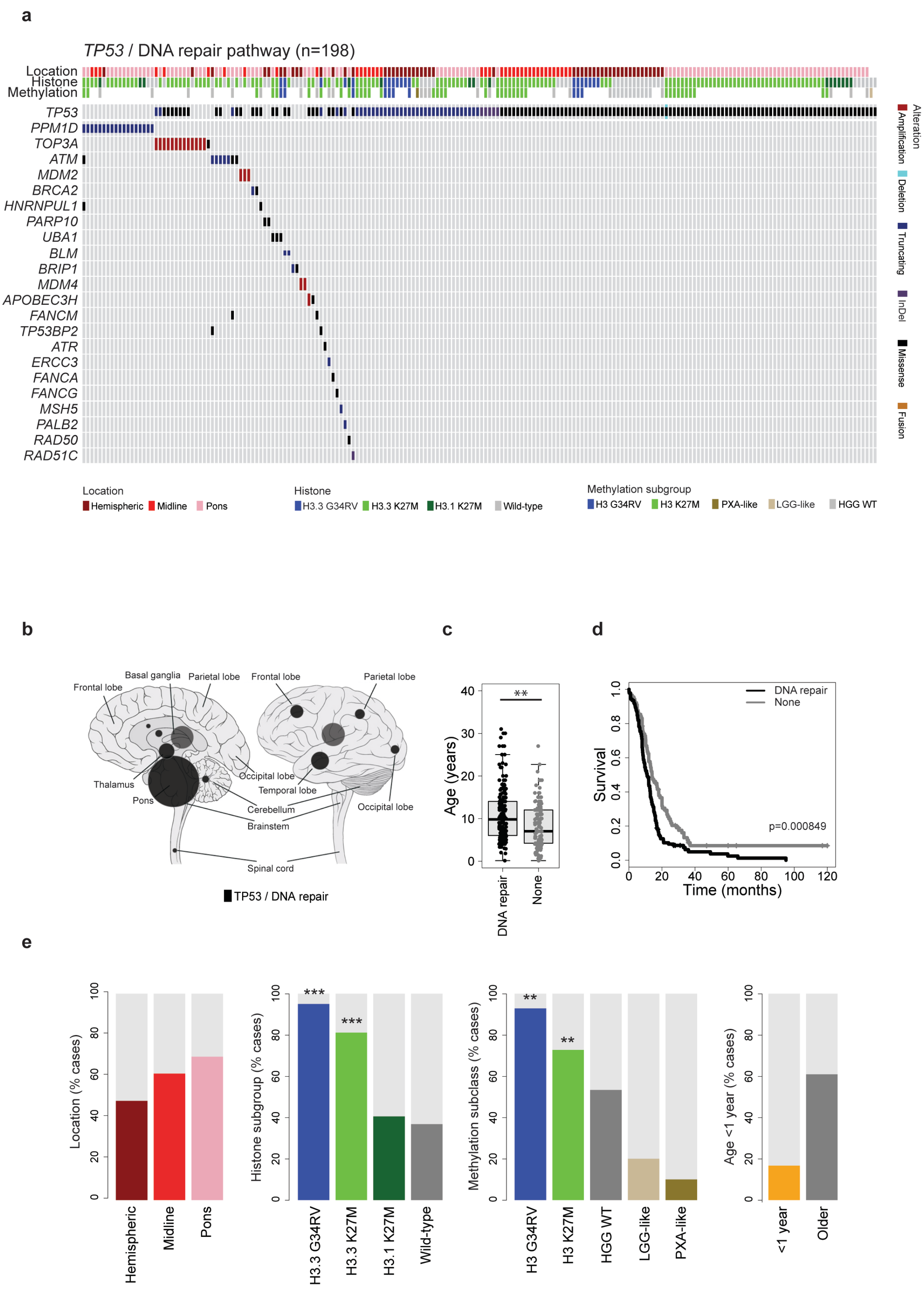

a
TP53 / DNA repair pathway (n=198)
Location
Histone
Methylation
TP53
PPM1D
TOP3A
ATM
MDM2
BRCA2
HNRNPUL1
PARP10
UBA1
BLM
BRIP1
MDM4
APOBEC3H
FANCM
TP53BP2
ATR
ERCC3
FANCA
FANCG
MSH5
PALB2
RAD50
RAD51C
Alteration
Amplification
Deletion
Truncating
InDel
Missense
Fusion
Location
Hemispheric Midline Pons
Histone
H3.3 G34RV H3.3 K27M H3.1 K27M Wild-type
Methylation subgroup
H3 G34RV H3 K27M PXA-like LGG-like HGG WT
b
Basal ganglia
Parietal lobe
Frontal lobe
Frontal lobe
Parietal lobe
Thalamus
Pons
Occipital lobe
Temporal lobe
Cerebellum
Brainstem
Occipital lobe
Spinal cord
TP53 / DNA repair
c
Age (years)
0
10
20
30
40
**
DNA repair
None
d
Survival
0.0
0.2
0.4
0.6
0.8
1.0
DNA repair
None
p=0.000849
0
20
40
60
80
100
120
Time (months)
e
Location (% cases)
Hemispheric
Midline
Pons
Histone subgroup (% cases)
***
***
H3.3 G34RV
H3.3 K27M
H3.1 K27M
Wild-type
Methylation subclass (% cases)
**
**
H3 G34RV
H3 K27M
HGG WT
LGG-like
PXA-like
Age <1 year (% cases)
<1 year
Older
0
20
40
60
80
100

B

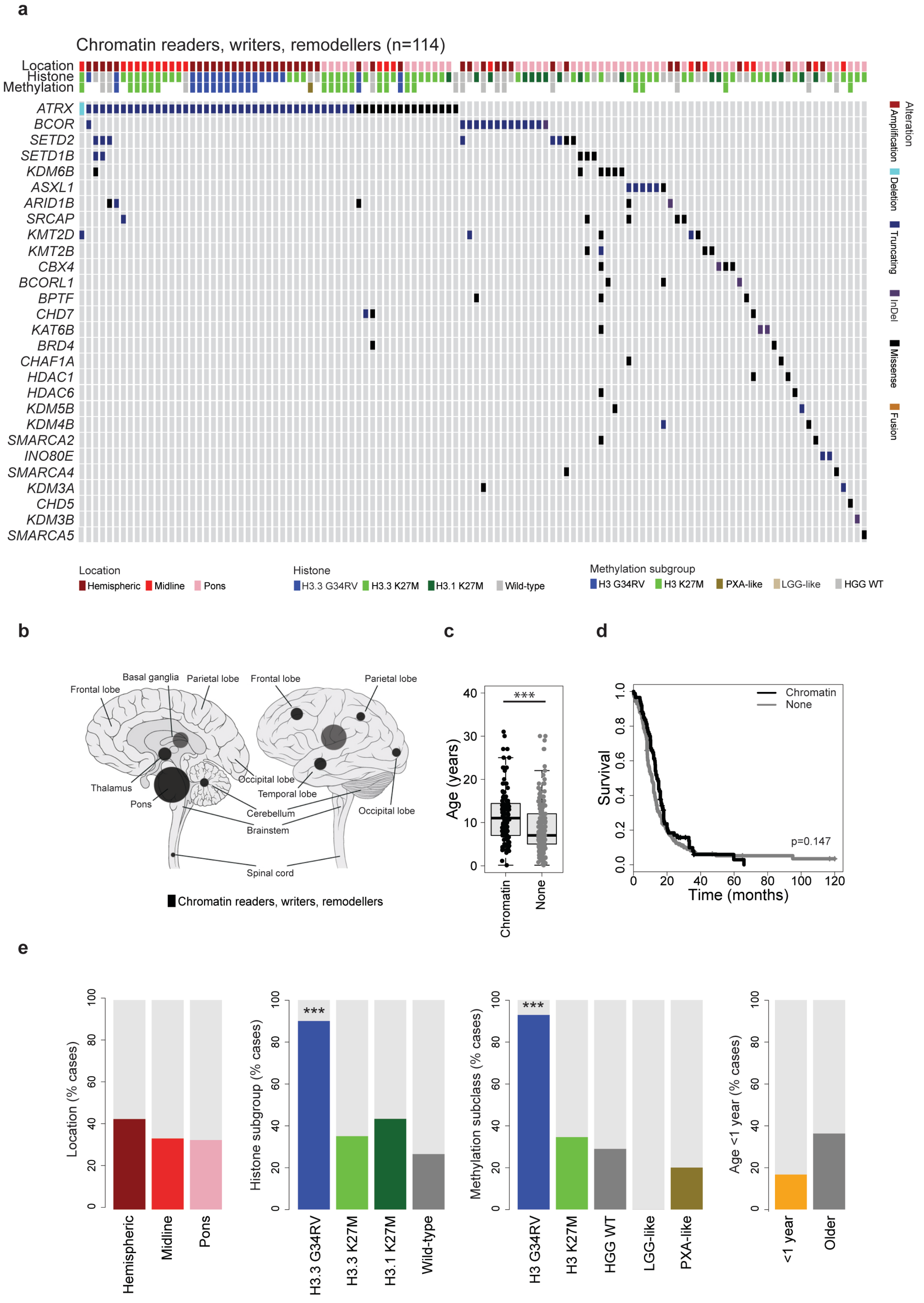
a
Chromatin readers, writers, remodellers (n=114)
Location
Histone
Methylation
ATRX
BCOR
SETD2
SETD1B
KDM6B
ASXL1
ARID1B
SRCAP
KMT2D
KMT2B
CBX4
BCORL1
BPTF
CHD7
KAT6B
BRD4
CHAF1A
HDAC1
HDAC6
KDM5B
KDM4B
SMARCA2
INO80E
SMARCA4
KDM3A
CHD5
KDM3B
SMARCA5
Alteration
Amplification
Deletion
Truncating
InDel
Missense
Fusion
Location
Hemispheric
Midline
Pons
Histone
H3.3 G34RV
H3.3 K27M
H3.1 K27M
Wild-type
Methylation subgroup
H3 G34RV
H3 K27M
PXA-like
LGG-like
HGG WT
b
Frontal lobe
Basal ganglia
Parietal lobe
Frontal lobe
Parietal lobe
Occipital lobe
Temporal lobe
Thalamus
Pons
Cerebellum
Brainstem
Occipital lobe
Spinal cord
Chromatin readers, writers, remodellers
c
Age (years)
***
Chromatin
None
d
Survival
Chromatin
None
p=0.147
Time (months)
e
Location (% cases)
Hemispheric
Midline
Pons
Histone subgroup (% cases)
***
H3.3 G34RV
H3.3 K27M
H3.1 K27M
Wild-type
Methylation subclass (% cases)
***
H3 G34RV
H3 K27M
HGG WT
LGG-like
PXA-like
Age <1 year (% cases)
<1 year
Older

C

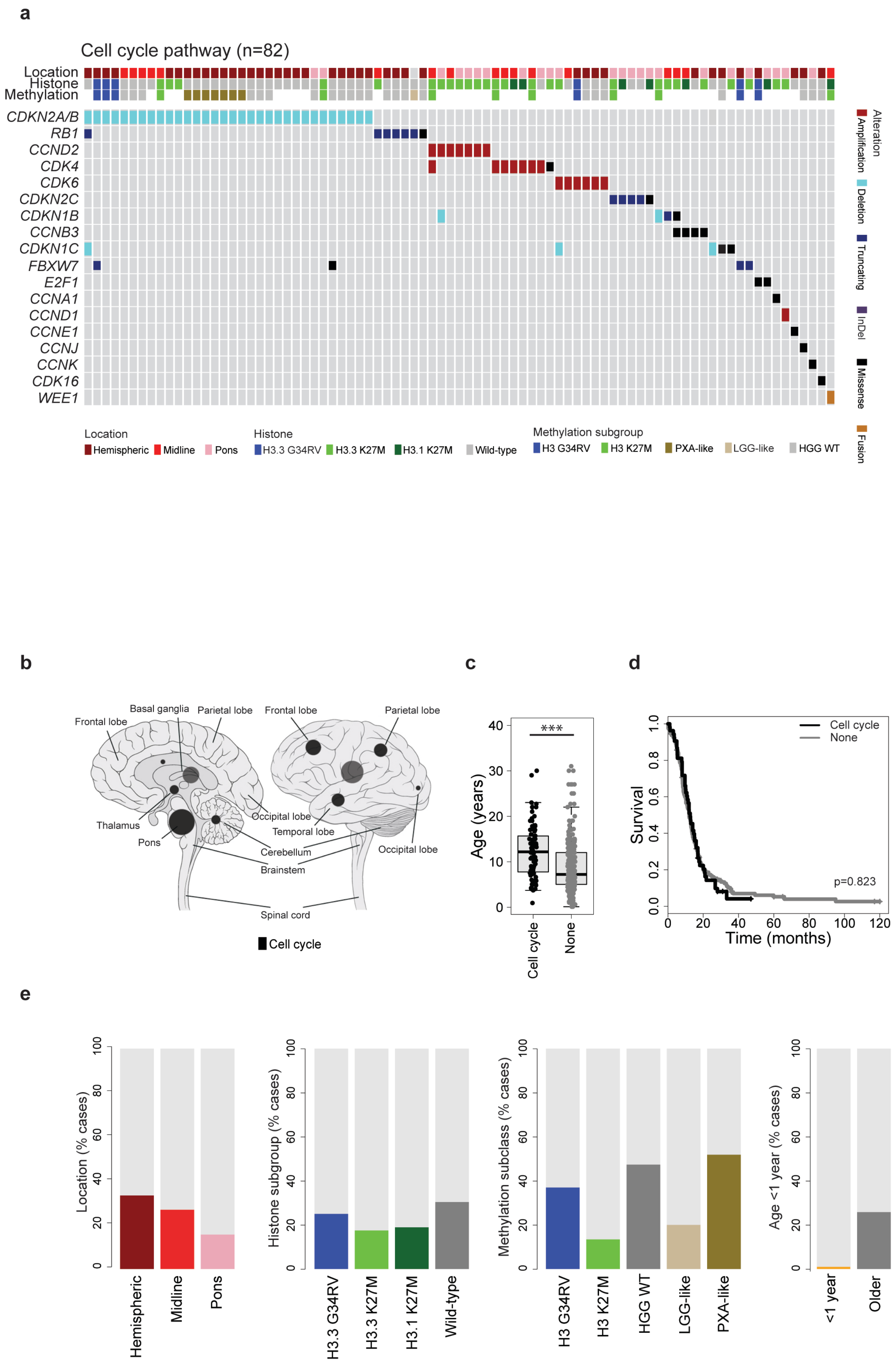
a
Cell cycle pathway (n=82)
Location
Histone
Methylation
CDKN2A/B
RB1
CCND2
CDK4
CDK6
CDKN2C
CDKN1B
CCNB3
CDKN1C
FBXW7
E2F1
CCNA1
CCND1
CCNE1
CCNJ
CCNK
CDK16
WEE1
Alteration
Amplification
Deletion
Truncating
InDel
Missense
Fusion
Location
Hemispheric
Midline
Pons
Histone
H3.3 G34RV
H3.3 K27M
H3.1 K27M
Wild-type
Methylation subgroup
H3 G34RV
H3 K27M
PXA-like
LGG-like
HGG WT
b
Frontal lobe
Basal ganglia
Parietal lobe
Frontal lobe
Parietal lobe
Thalamus
Pons
Occipital lobe
Temporal lobe
Cerebellum
Occipital lobe
Brainstem
Spinal cord
Cell cycle
c
Age (years)
***
Cell cycle
None
d
Survival
Cell cycle
None
p=0.823
Time (months)
e
Location (% cases)
Hemispheric
Midline
Pons
Histone subgroup (% cases)
H3.3 G34RV
H3.3 K27M
H3.1 K27M
Wild-type
Methylation subclass (% cases)
H3 G34RV
H3 K27M
HGG WT
LGG-like
PXA-like
Age <1 year (% cases)
<1 year
Older

D

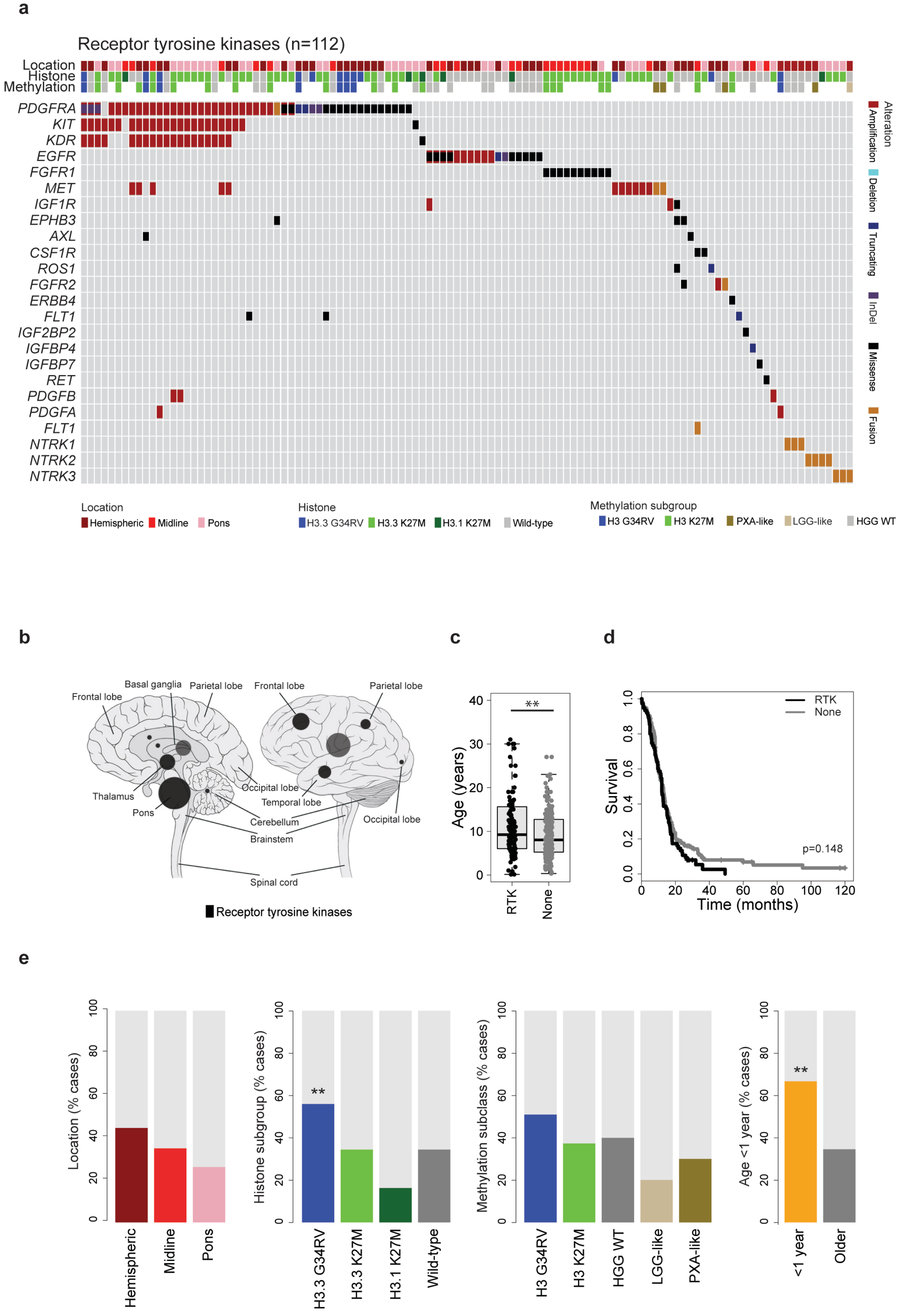

a
Receptor tyrosine kinases (n=112)
Location
Histone
Methylation
PDGFRA
KIT
KDR
EGFR
FGFR1
MET
IGF1R
EPHB3
AXL
CSF1R
ROS1
FGFR2
ERBB4
FLT1
IGF2BP2
IGFBP4
IGFBP7
RET
PDGFB
PDGFA
FLT1
NTRK1
NTRK2
NTRK3
Alteration
Amplification
Deletion
Truncating
InDel
Missense
Fusion
Location
Hemispheric
Midline
Pons
Histone
H3.3 G34RV
H3.3 K27M
H3.1 K27M
Wild-type
Methylation subgroup
H3 G34RV
H3 K27M
PXA-like
LGG-like
HGG WT
b
Frontal lobe
Basal ganglia
Parietal lobe
Frontal lobe
Parietal lobe
Thalamus
Pons
Occipital lobe
Temporal lobe
Cerebellum
Brainstem
Occipital lobe
Spinal cord
Receptor tyrosine kinases
c
Age (years)
**
RTK
None
d
Survival
RTK
None
p=0.148
Time (months)
e
Location (% cases)
Hemispheric
Midline
Pons
Histone subgroup (% cases)
**
H3.3 G34RV
H3.3 K27M
H3.1 K27M
Wild-type
Methylation subclass (% cases)
H3 G34RV
H3 K27M
HGG WT
LGG-like
PXA-like
Age <1 year (% cases)
**
<1 year
Older

E

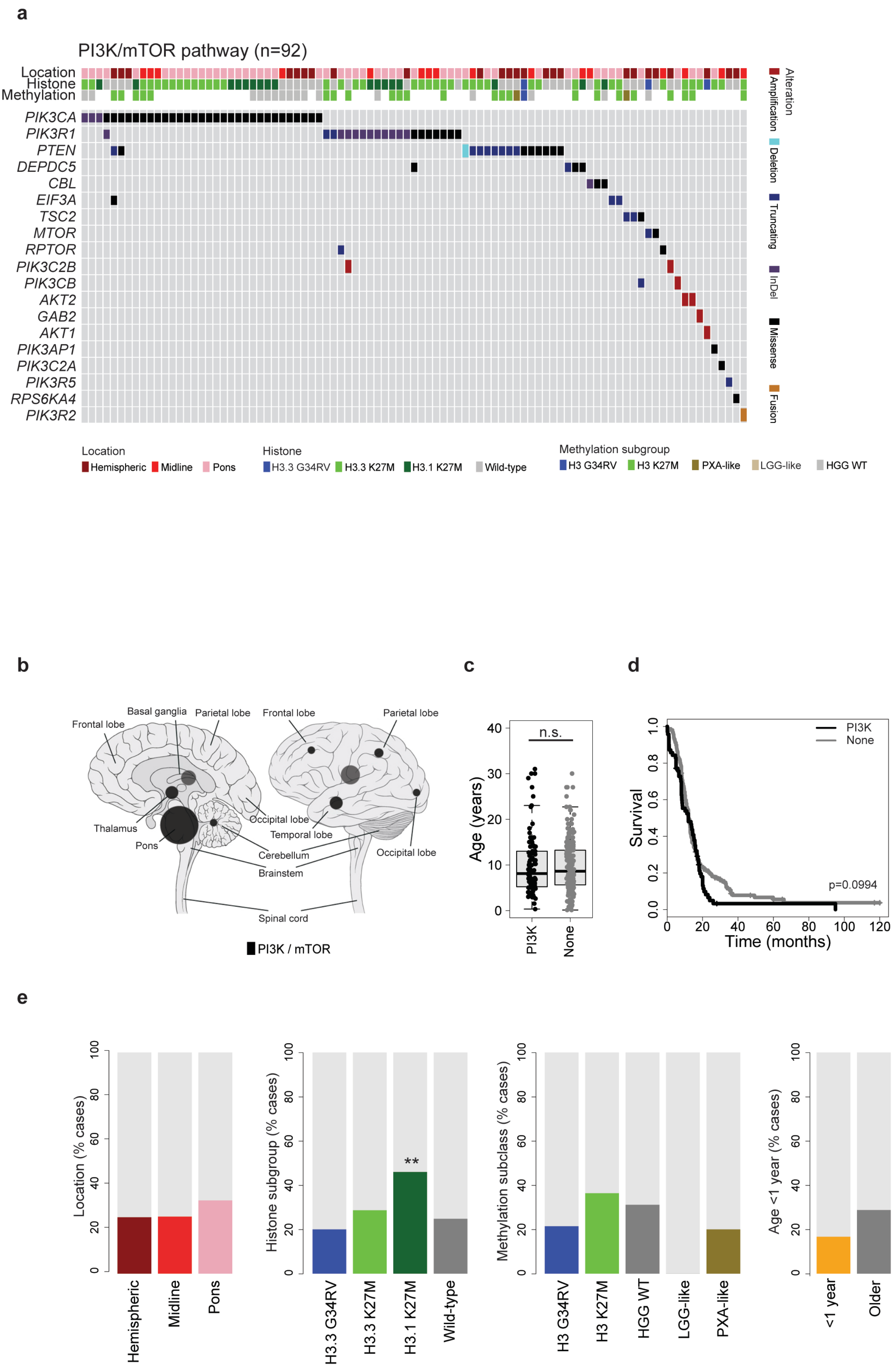
a
PI3K/mTOR pathway (n=92)
Location
Histone
Methylation
PIK3CA
PIK3R1
PTEN
DEPDC5
CBL
EIF3A
TSC2
MTOR
RPTOR
PIK3C2B
PIK3CB
AKT2
GAB2
AKT1
PIK3AP1
PIK3C2A
PIK3R5
RPS6KA4
PIK3R2
Alteration
Amplification
Deletion
Truncating
InDel
Missense
Fusion
Location
Hemispheric
Midline
Pons
Histone
H3.3 G34RV
H3.3 K27M
H3.1 K27M
Wild-type
Methylation subgroup
H3 G34RV
H3 K27M
PXA-like
LGG-like
HGG WT
b
Basal ganglia
Parietal lobe
Frontal lobe
Frontal lobe
Parietal lobe
Thalamus
Pons
Occipital lobe
Temporal lobe
Cerebellum
Occipital lobe
Brainstem
Spinal cord
PI3K / mTOR
c
n.s.
Age (years)
PI3K
None
d
PI3K
None
Survival
p=0.0994
Time (months)
e
Location (% cases)
Hemispheric
Midline
Pons
Histone subgroup (% cases)
**
H3.3 G34RV
H3.3 K27M
H3.1 K27M
Wild-type
Methylation subclass (% cases)
H3 G34RV
H3 K27M
HGG WT
LGG-like
PXA-like
Age <1 year (% cases)
<1 year
Older

F

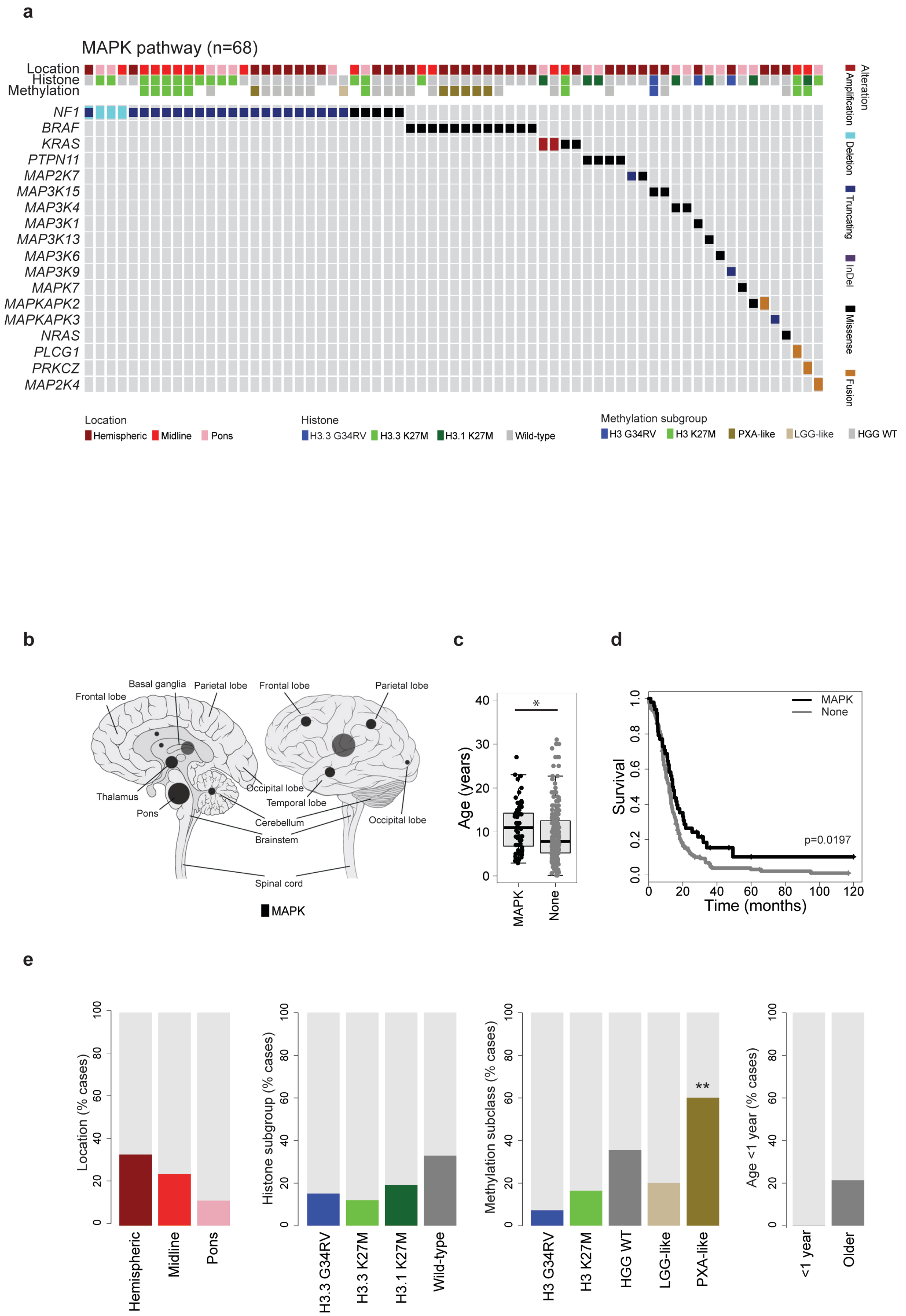
a
MAPK pathway (n=68)
Location
Histone
Methylation
NF1
BRAF
KRAS
PTPN11
MAP2K7
MAP3K15
MAP3K4
MAP3K1
MAP3K13
MAP3K6
MAP3K9
MAPK7
MAPKAPK2
MAPKAPK3
NRAS
PLCG1
PRKCZ
MAP2K4
Alteration
Amplification
Deletion
Truncating
InDel
Missense
Fusion
Location
Hemispheric
Midline
Pons
Histone
H3.3 G34RV
H3.3 K27M
H3.1 K27M
Wild-type
Methylation subgroup
H3 G34RV
H3 K27M
PXA-like
LGG-like
HGG WT
b
Basal ganglia
Parietal lobe
Frontal lobe
Frontal lobe
Parietal lobe
Thalamus
Pons
Occipital lobe
Temporal lobe
Cerebellum
Brainstem
Occipital lobe
Spinal cord
MAPK
c
Age (years)
*
MAPK
None
d
Survival
MAPK
None
p=0.0197
Time (months)
e
Location (% cases)
Hemispheric
Midline
Pons
Histone subgroup (% cases)
H3.3 G34RV
H3.3 K27M
H3.1 K27M
Wild-type
Methylation subclass (% cases)
**
H3 G34RV
H3 K27M
HGG WT
LGG-like
PXA-like
Age <1 year (% cases)
<1 year
Older

G

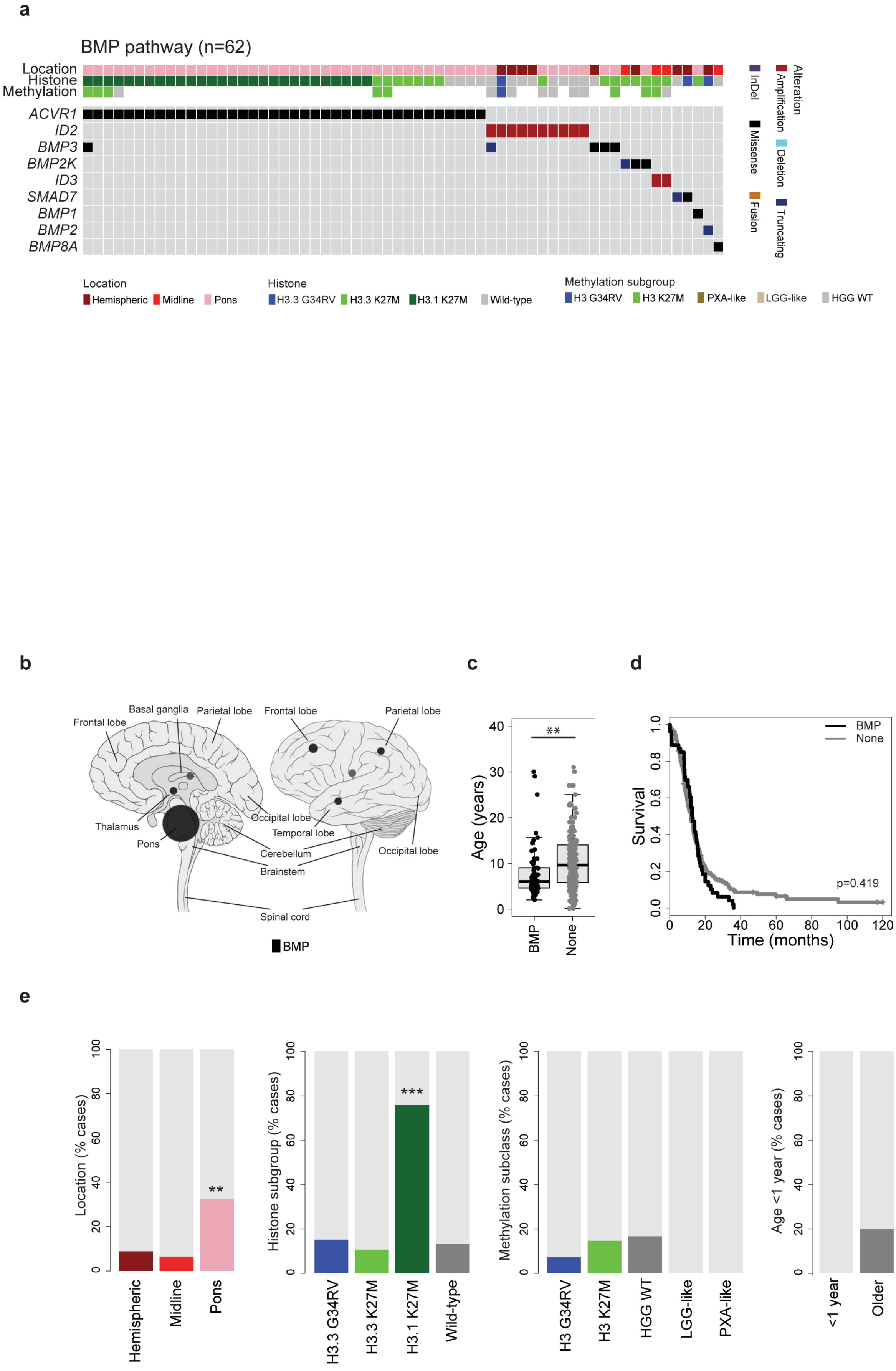

a
BMP pathway (n=62)
Location
Histone
Methylation
ACVR1
ID2
BMP3
BMP2K
ID3
SMAD7
BMP1
BMP2
BMP8A
Alteration
Amplification
Deletion
Truncating
InDel
Missense
Fusion
Location
Hemispheric
Midline
Pons
Histone
H3.3 G34RV
H3.3 K27M
H3.1 K27M
Wild-type
Methylation subgroup
H3 G34RV
H3 K27M
PXA-like
LGG-like
HGG WT
b
Basal ganglia
Parietal lobe
Frontal lobe
Frontal lobe
Parietal lobe
Thalamus
Pons
Occipital lobe
Temporal lobe
Cerebellum
Brainstem
Occipital lobe
Spinal cord
BMP
c
Age (years)
**
BMP
None
d
Survival
Time (months)
BMP
None
p=0.419
e
Location (% cases)
Hemispheric
Midline
Pons
Histone subgroup (% cases)
H3.3 G34RV
H3.3 K27M
H3.1 K27M
Wild-type
***
Methylation subclass (% cases)
H3 G34RV
H3 K27M
HGG WT
LGG-like
PXA-like
Age <1 year (% cases)
<1 year
Older

H

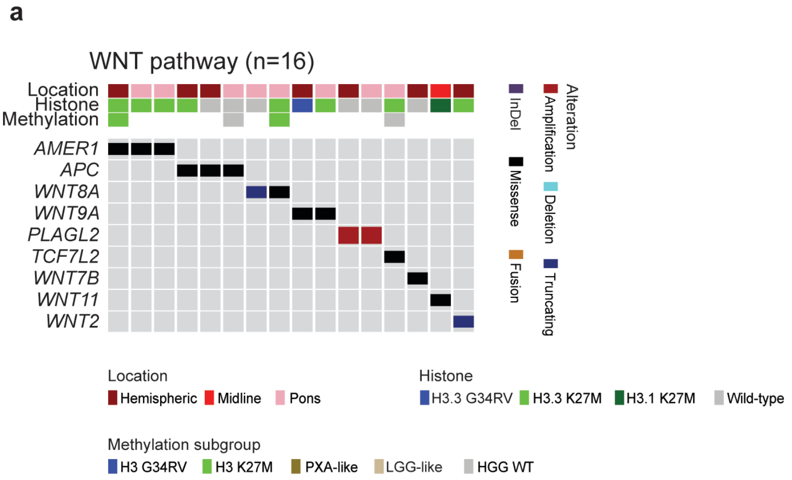
a
WNT pathway (n=16)
Location
Histone
Methylation
AMER1
APC
WNT8A
WNT9A
PLAGL2
TCF7L2
WNT7B
WNT11
WNT2
Alteration
Amplification
Deletion
Truncating
InDel
Missense
Fusion
Location
Hemispheric
Midline
Pons
Histone
H3.3 G34RV
H3.3 K27M
H3.1 K27M
Wild-type
Methylation subgroup
H3 G34RV
H3 K27M
PXA-like
LGG-like
HGG WT

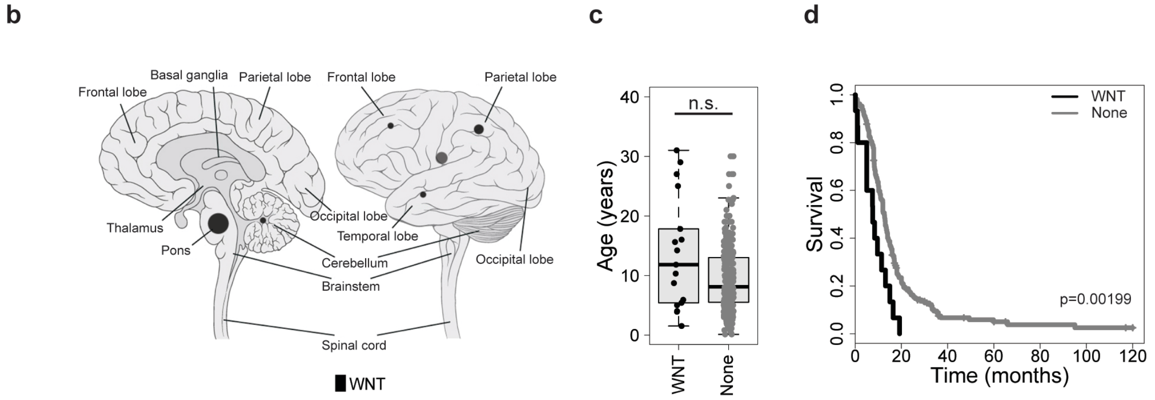
b
Basal ganglia
Parietal lobe
Frontal lobe
Frontal lobe
Parietal lobe
Thalamus
Pons
Occipital lobe
Temporal lobe
Cerebellum
Brainstem
Occipital lobe
Spinal cord
WNT
c
n.s.
Age (years)
WNT
None
d
WNT
None
Survival
p=0.00199
Time (months)

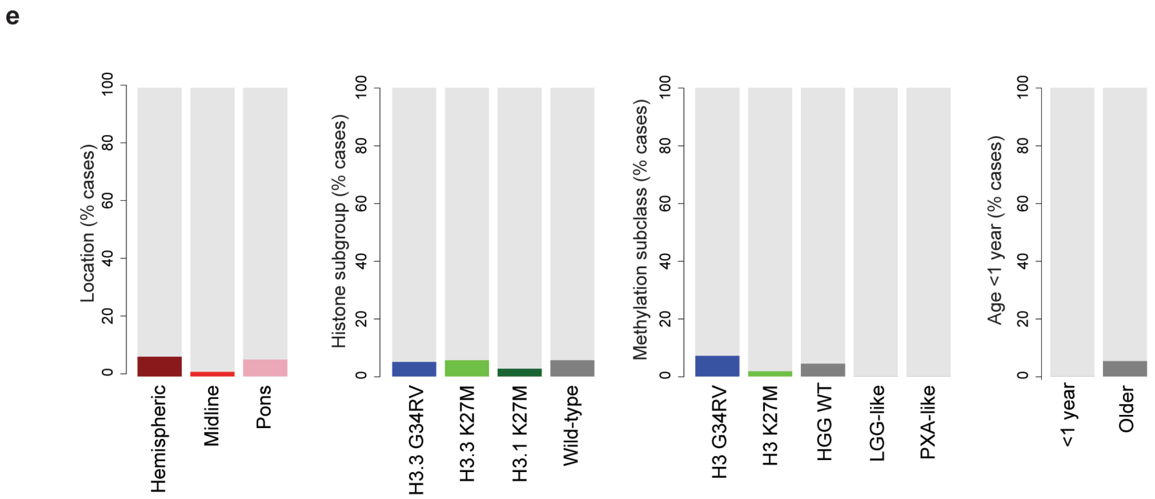
e
Location (% cases)
Hemispheric
Midline
Pons
Histone subgroup (% cases)
H3.3 G34RV
H3.3 K27M
H3.1 K27M
Wild-type
Methylation subclass (% cases)
H3 G34RV
H3 K27M
HGG WT
LGG-like
PXA-like
Age <1 year (% cases)
<1 year
Older

l

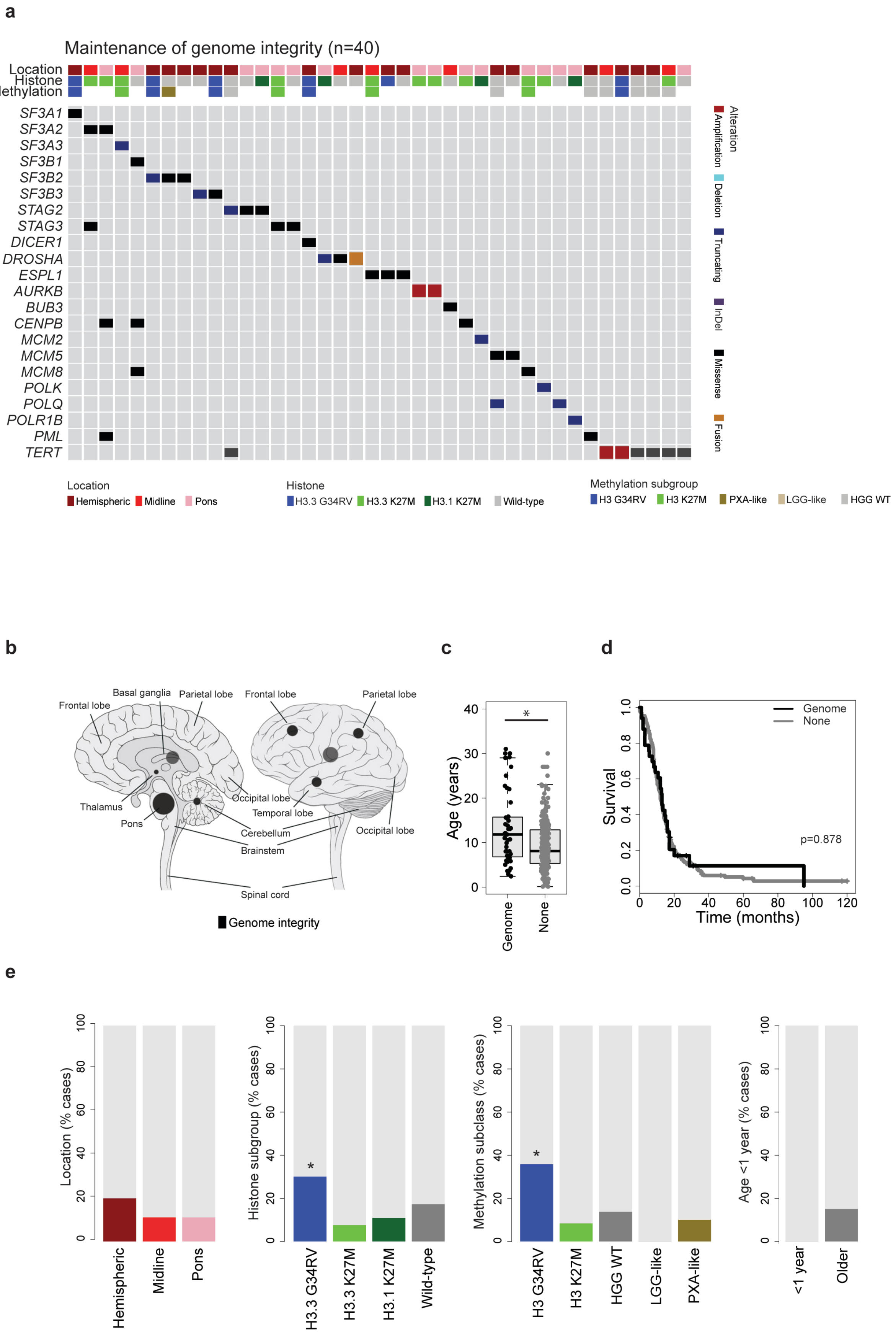
a
Maintenance of genome integrity (n=40)
Location
Histone
Methylation
SF3A1
SF3A2
SF3A3
SF3B1
SF3B2
SF3B3
STAG2
STAG3
DICER1
DROSHA
ESPL1
AURKB
BUB3
CENPB
MCM2
MCM5
MCM8
POLK
POLQ
POLR1B
PML
TERT
Alteration
Amplification
Deletion
Truncating
InDel
Missense
Fusion
Location
Hemispheric
Midline
Pons
Histone
H3.3 G34RV
H3.3 K27M
H3.1 K27M
Wild-type
Methylation subgroup
H3 G34RV
H3 K27M
PXA-like
LGG-like
HGG WT
b
Frontal lobe
Basal ganglia
Parietal lobe
Frontal lobe
Parietal lobe
Thalamus
Pons
Occipital lobe
Temporal lobe
Cerebellum
Brainstem
Occipital lobe
Spinal cord
Genome integrity
c
Age (years)
Genome
None
d
Genome
None
Survival
p=0.878
Time (months)
e
Location (% cases)
Hemispheric
Midline
Pons
Histone subgroup (% cases)
H3.3 G34RV
H3.3 K27M
H3.1 K27M
Wild-type
Methylation subclass (% cases)
H3 G34RV
H3 K27M
HGG WT
LGG-like
PXA-like
Age <1 year (% cases)
<1 year
Older

Figure S7 (related to Figure 7) – Integrated pathway analysis of pHGG/DIPG. (A) TP53 / DNA repair pathway, (B) chromatin readers, writers, remodellers, (C) cell cycle pathway, (D) receptor tyrosine kinases, (E) PI3K/mTOR pathway, (F) MAPK pathway, (G) BMP pathway, (H) WNT pathway and (I) maintenance of genome integrity. (a) Oncoprint representation of an integrated annotation of somatic mutations and DNA copy number changes in cases targeting one or more members of a specific pathway. Clinicopathological and molecular annotations are provided as bars according to the included key. (b) Anatomical location of pathway-targeted cases. Radius of circle is proportional to the number of cases. Left – sagittal section showing internal structures; right – external view highlighting cerebral lobes. Black = given pathway alteration. Lighter shaded circles represent a non-specific designation of hemispheric, midline or brainstem. (c) Boxplot showing age at diagnosis of included cases, separated by pathway-targeted cases. The thick line within the box is the median, the lower and upper limits of the boxes represent the first and third quartiles, and the whiskers 1.5x the interquartile range. (d) Kaplan-Meier plot of overall survival of cases separated by pathway-targeted cases, p value calculated by the log-rank test. (e) Barplots showing distribution of pathway-targeted cases by anatomical location, histone subgroup, methylation subgroup and age. *** $p<0.001$, ** $p<0.01$, *$p<0.05$, n.s., not significant, t-test.

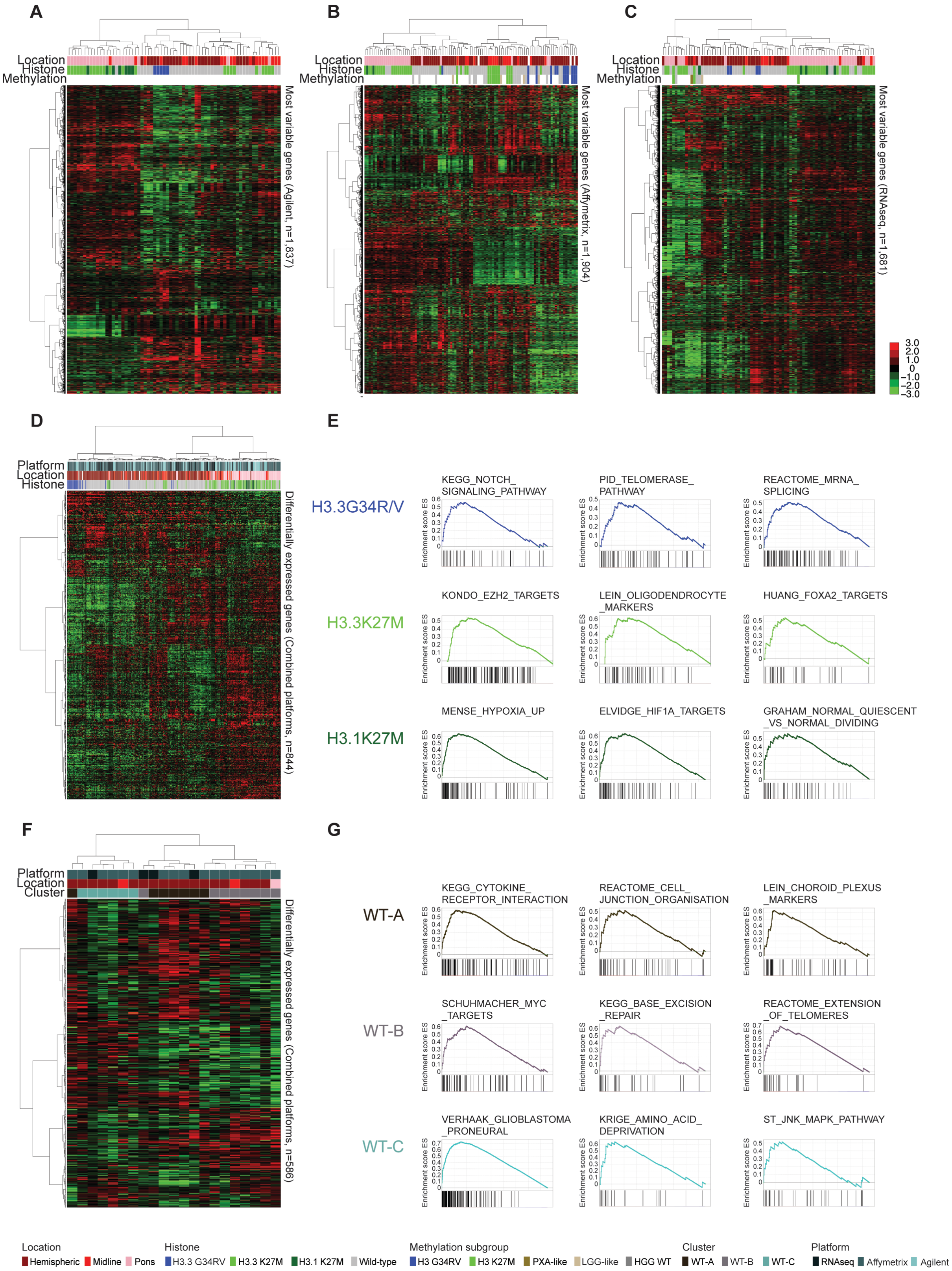
A
B
C
Location
Histone
Methylation
Most variable genes (Agilent, n=1,837)
Most variable genes (Affymetrix, n=1,904)
Most variable genes (RNAseq, n=1,681)
3.0
2.0
1.0
0
−1.0
−2.0
−3.0
D
Platform
Differentially expressed genes (Combined platforms, n=844)
E
H3.3G34R/V
KEGG_NOTCH_SIGNALING_PATHWAY
PID_TELOMERASE_PATHWAY
REACTOME_MRNA_SPLICING
H3.3K27M
KONDO_EZH2_TARGETS
LEIN_OLIGODENDROCYTE_MARKERS
HUANG_FOXA2_TARGETS
H3.1K27M
MENSE_HYPOXIA_UP
ELVIDGE_HIF1A_TARGETS
GRAHAM_NORMAL_QUIESCENT_VS_NORMAL_DIVIDING
Enrichment score ES
F
Cluster
Differentially expressed genes (Combined platforms, n=586)
G
WT-A
KEGG_CYTOKINE_RECEPTOR_INTERACTION
REACTOME_CELL_JUNCTION_ORGANISATION
LEIN_CHOROID_PLEXUS_MARKERS
WT-B
SCHUHMACHER_MYC_TARGETS
KEGG_BASE_EXCISION_REPAIR
REACTOME_EXTENSION_OF_TELOMERES
WT-C
VERHAAK_GLIOBLASTOMA_PRONEURAL
KRIGE_AMINO_ACID_DEPRIVATION
ST_JNK_MAPK_PATHWAY
Location: Hemispheric, Midline, Pons
Histone: H3.3 G34RV, H3.3 K27M, H3.1 K27M, Wild-type
Methylation subgroup: H3 G34RV, H3 K27M, PXA-like, LGG-like, HGG WT
Cluster: WT-A, WT-B, WT-C
Platform: RNAseq, Affymetrix, Agilent

Figure S8 (related to Figure 8) – Integrated analysis of H3/IDH1 wild-type pHGG/DIPG. (A-C) Unsupervised hierarchical clustering of the most variable genes in samples profiled by (A) Agilent arrays (n=67), (B) Affymetrix arrays (n=102) and/or (C) RNA sequencing (n=82). (D) A combined dataset across all platforms (n=220) is subjected to differential expression analysis for H3.3G34R/V, H3.3K27M and H3.1K27M mutations, with significant genes plotted in the heatmap. (E) Gene set enrichment analysis for selected significant gene sets for each comparison. (F) Combined dataset for H3/IDH1 wild-type clusters WT-A, WT-B and WT-C (n=21), with significantly differentially expressed genes plotted in the heatmap. (G) Gene set enrichment analysis for selected significant gene sets for each comparison. Clinicopathological and molecular annotations are provided as bars according to the included key.
